# 5-hydroxymethylcytosine Marks Mammalian Origins Acting as a Barrier to Replication

**DOI:** 10.1038/s41598-019-47528-3

**Published:** 2019-07-30

**Authors:** Terezia Prikrylova, Julia Robertson, Francesca Ferrucci, Dorota Konorska, Håvard Aanes, Adeel Manaf, Beibei Zhang, Cathrine Broberg Vågbø, Anna Kuśnierczyk, Karin M. Gilljam, Caroline Løvkvam-Køster, Marit Otterlei, John Arne Dahl, Jorrit Enserink, Arne Klungland, Adam B. Robertson

**Affiliations:** 1 Institute of Medical Microbiology, Oslo University Hospital, Rikshospitalet, Norway; 2 Department of Molecular Cell Biology, Institute for Cancer Research, Oslo University Hospital, Montebello, 0379 Oslo Norway; 3 Proteomics and Metabolomics Core Facility, Norwegian University of Science and Technology, 7491 Trondheim, Norway; 4 Centre for Cancer Cell Reprogramming, Institute of Clinical Medicine, Faculty of Medicine, Montebello, 0379 Oslo Norway; 5 The Department of Biosciences, Faculty of Mathematics and Natural Sciences, University of Oslo, Oslo, Norway; 6 Institute of Basic Medical Sciences, University of Oslo, PO Box 1018 Blindern, N-0315 Oslo, Norway; 7 Present Address: Hemispherian AS, Gaustadalleen 21, 0349 Oslo, Norway

**Keywords:** Origin firing, DNA

## Abstract

In most mammalian cells, DNA replication occurs once, and only once between cell divisions. Replication initiation is a highly regulated process with redundant mechanisms that prevent errant initiation events. In lower eukaryotes, replication is initiated from a defined consensus sequence, whereas a consensus sequence delineating mammalian origin of replication has not been identified. Here we show that 5-hydroxymethylcytosine (5hmC) is present at mammalian replication origins. Our data support the hypothesis that 5hmC has a role in cell cycle regulation. We show that 5hmC level is inversely proportional to proliferation; indeed, 5hmC negatively influences cell division by increasing the time a cell resides in G1. Our data suggest that 5hmC recruits replication-licensing factors, then is removed prior to or during origin firing. Later we propose that TET2, the enzyme catalyzing 5mC to 5hmC conversion, acts as barrier to rereplication. In a broader context, our results significantly advance the understating of 5hmC involvement in cell proliferation and disease states.

## Introduction

Mammalian DNA content is maintained throughout cell division ensuring that the entire genome is replicated precisely one time prior to each cell division (for comprehensive reviews see Bell and Dutta^1^ and Fragkos *et al*.^2^). In all but a few circumstances, DNA replication in the absence of cell division is harmful to the cell and the organism^3^. Incomplete DNA replication or rereplication of any part of the genome often results in genomic instability^4^. Preventing genomic errors, the cell has evolved complex mechanisms that allow the genome to be rapidly synthesized and eliminates the possibility of harmful resynthesis events^5^. This control over DNA replication is achieved through replication licensing^6,7^.

Replication licensing is well described in lower eukaryotes, including *S. cerevasiae*; mammalian replication licensing is less well understood. Mammalian replication occurs in a two-step process: (i) in G1 and M phases, elements of the pre-replication (pre-RC) complex bind DNA – origin licensing – and (ii) as the cell progresses to S phase, selected pre-RC complexes are activated. These activated complexes are stimulated to initiate origin firing (Reviewed by Masai, H *et al*.^8^). The pre-RC complex comprises: six origin recognition proteins (ORC1-6) which form the origin recognition complex (ORC) at the origin of replication; CDC6; CDT1; and the proposed replicative helicase MCM2-7^8–11^. The pre-RC is phosphorylated on the replicative helicase by cyclin dependent kinases (CDKs) and a Dbf4-dependent kinase (DDK)^12,13^. The pre-RC phosphorylation is combined with the recruitment and phosphorylation of several proteins, including CDC45, MCM10, the GINS proteins, RECQL4, TOPBP1, and Treslin^14–17^. These modifications, together with the release of CDC6 and CDT1 result in the formation of the pre-initiation complex (pre-IC)^18^. The recruitment of RPA, RFC, PCNA, and DNA polymerases α, ε, and δ, to the pre-IC and the release of the MCM2-7 helicase initiate replication^19,20^. Several redundant mechanisms prevent rereplication: (i) Geminin binds and inhibits CDT1 activity^21^, (ii) CDT1 is degraded^22^, and (iii) CDC6 is exported from the nucleus^23^. Inappropriate expression of CDT1, CDC6, and/or Geminin is sufficient to cause genomic rereplication, often resulting in polyploidy^24^. Interestingly, dividing mammalian cells assemble significantly more potential origins than are used during genome duplication. While the cause for this phenomenon is unknown, some groups have speculated that multiple inactive origins are licensed to ensure genome integrity during genetically harmful events.

Replication is initiated at specific locations in the mammalian genome; however, no mammalian replication origin consensus sequence is known. Mammalian origins are over-represented in GC rich regions, including CpG islands^25–28^ and interestingly, mammalian origins of replication also appear to be heritable from one cell division to the next. Based on these and other evidence, many researchers have linked replication origins to epigenetic factors including DNA and chromatin modifications^2,28–31^. Among DNA modifications, 5-methylcytosine (5mC) is present at some, but not all replication origins^32^. Mammalian replication origins positively correlate with regions of active transcription^33–35^ and as a parallel, the newly identified base 5-hydroxymethylcytosine (5hmC) correlates with regions of active transcription^36–38^.

5-hydroxymethylcytosine is a relatively recently discovered DNA base in mammalian cells^36,39^. While the function of 5hmC is unclear, its presence at promoters has been correlated with transcription^40–43^. 5hmC is the product of enzyme-catalyzed oxidation of 5-methylcytosine (5mC). Presently, three enzymes have been described to catalyze this oxidation reaction: Tet1, Tet2, and Tet3^44^. Initial reports suggest that 5hmC is involved in a variety of cellular processes, including stem cell pluripotency, tumorigenesis, cell fate, embryogenesis and differentiation^45–48^. 5hmC has been suggested to affect these processes through transcriptional regulation.

5hmC is widely distributed throughout the mammalian genome and is enriched to the greatest extent within gene bodies and to a relatively lesser degree at promoters^49–52^. In biochemical assays the presence of 5hmC at promoters strongly inhibits transcription, while the presence of 5hmC within gene bodies has almost no effect on transcription^53^. *In vivo* assays that correlate 5hmC with transcription have produced mixed results: high 5hmC levels at a subset of mammalian promoters correlate with increased transcription, at other promoters high 5hmC levels correlate with reduced gene expression^36,38,44,54,55^. As 5hmC is present throughout the genome, its presence at non-promoter regions suggests an alternative function for this highly modified base.

Since (i) 5hmC is found within gene bodies regions, (ii) the presence of 5hmC within gene bodies regions has almost no detectable effect on transcription, (iii), transcription coincides with active origins of replication, (iv) both 5hmC and origins of replication appear to be heritable, we hypothesized that 5hmC could play a role in replication.

In this manuscript, we demonstrate the role of 5hmC in cell cycle regulation. We show that selected replication licensing components bind to 5hmC-modified DNA and we propose that 5hmC marks replication origins. Our data indicate that 5hmC is globally enriched at replication origins and that 5hmC is depleted at recently fired origins. Global 5hmC levels are inversely correlated with proliferation; indeed, cell cycle analysis demonstrates that high 5hmC levels significantly increase the time a cell spends in G1 phase. Taken together, our data provide a better understanding of 5hmC involvement in cell proliferation and disease states.

## Results

### Proteins involved in genome maintenance and cell cycle bind preferentially to 5-hydroxymethylcytosine-modified DNA

**Figure 1:**
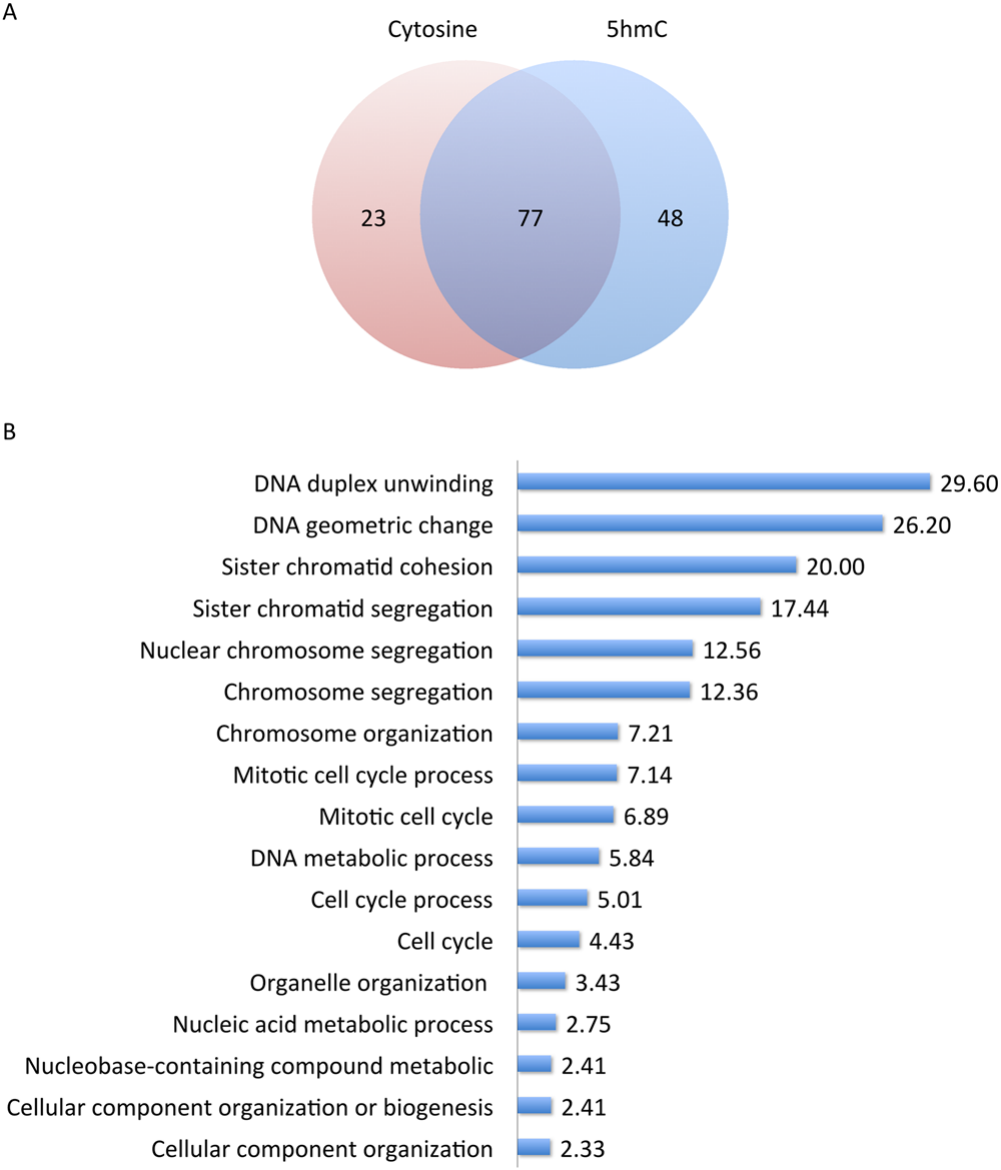
Proteins that exclusively bind 5hmC-modified DNA are involved in cell cycle and chromosome maintenance. (**A**) HeLa nuclear extracts were incubated with unmodified and 5hmC- modified DNA under conditions favoring DNA binding. Proteins that bound to each substrate were resolved using SDS-PAGE and identified by mass spectrometry. A significant fraction of proteins interacted with both unmodified DNA and 5hmC-modified DNA. (**B**) Proteins that interacted exclusively with 5hmC-modified DNA were subjected to a Panther Gene Ontology over-representation test. Results of the over-representation test are displayed as fold above expected for a random protein population.

Beads coated with unmodified or 5hmC-modified DNA substrates were incubated with HeLa nuclear extracts (Supplemental Fig. S1). Substrates were recovered and weak DNA binding proteins were washed from the sample. Notably, the binding and wash buffers contain EDTA, which inhibits a 5hmC-specific nuclease^56^. As equal portions of unmodified DNA and 5hmC-modified DNA were loaded onto the beads (Supplemental Fig. S2A) with similar recovery efficiencies (Supplemental Fig. S2B), the beads did not introduce experimental bias. Proteins bound to unmodified or 5hmC-modified DNA were identified through Electrospray and MALDI Mass Spectrometry (Supplemental Fig. S3; Table S1). Selected window of protein masses allowed us to eliminate degradation products that produced confounding results. We identified 100 distinct proteins that interact with unmodified DNA (Supplemental Fig. S4, and Table S2) and 125 distinct proteins that interact with 5hmC-modified DNA. Proteins that interacted with both the unmodified and 5hmC-modified substrates (Supplemental Fig. S5 and Table S3) were eliminated from the analysis, yielding 48 proteins that specifically interact with 5hmC-modified DNA (Fig. 1A). These proteins were analyzed using a gene ontology over-representation test^57^. Proteins that appeared at the top of our over-representation list included proteins involved in mitosis and chromosome organization (Fig. 1B and Supplemental Table S4). Interestingly, proteins involved in transcription were neither over-represented nor under-represented in this sample. Our results suggest that proteins that interact with 5hmC may be involved in mitosis or maintenance of chromosome integrity.

### The MCM2-7 helicase binds to 5hmC-modified DNA *in vitro* and *in vivo*

**Figure 2:**
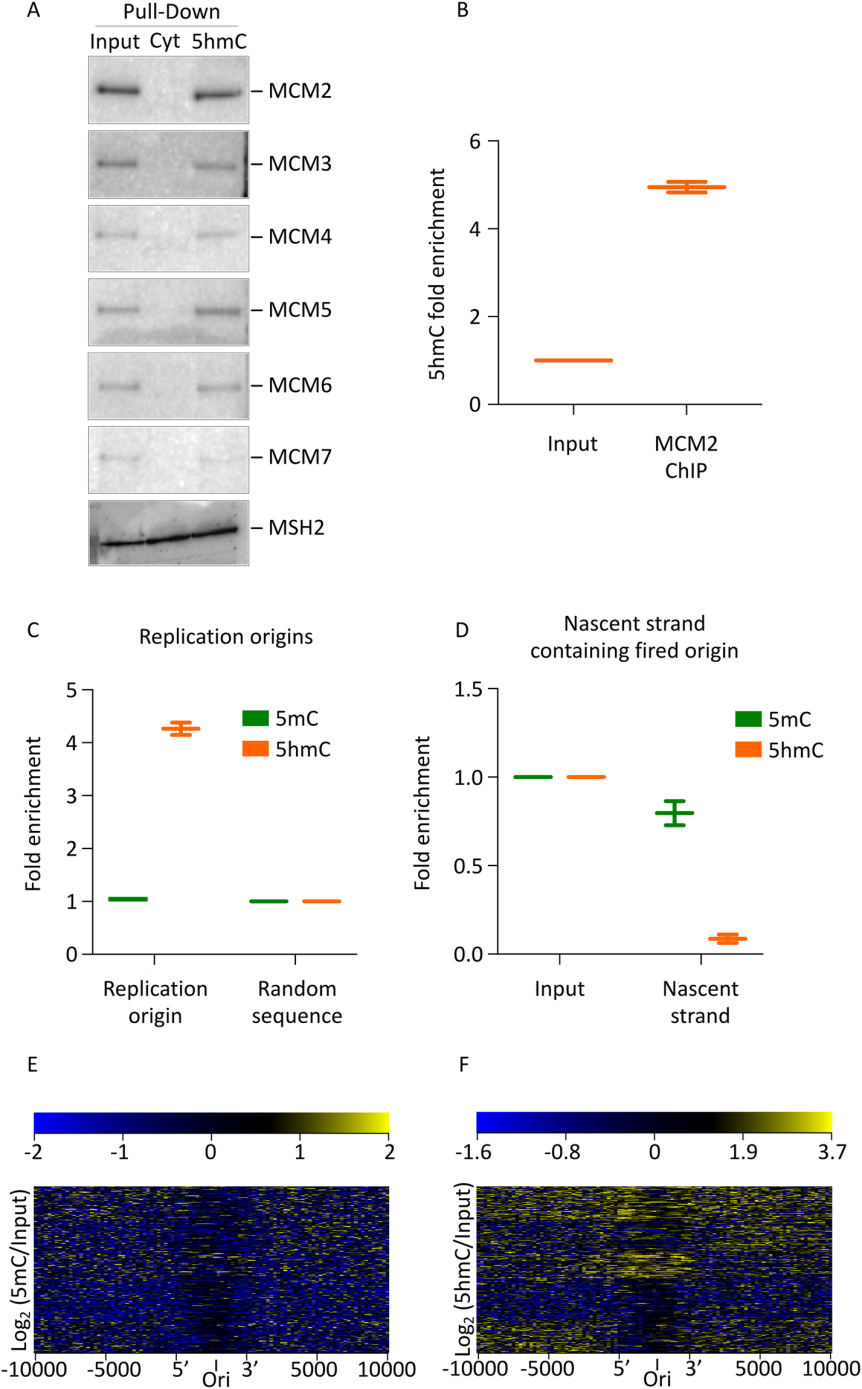
The MCM2-7 replication licensing factor and replicative helicase interacts preferentially with 5hmC-modified DNA; added and 5hmC dynamics at replication origins. (**A**) Unmodified and 5hmC-modified DNA substrates are incubated with HeLa nuclear extracts. The DNA:Bead complexes are recovered, resolved on SDS-PAGE, and probed with antisera raised against each of the MCM2-7 helicase subunits. (**B**) The 5hmC modification is enriched in DNA that is immunoprecipitated with MCM2 antiserum; p-value < 0.001. (**C**) Genomic 5mC and 5hmC content compared to 5mC and 5hmC content at replication origins; 5hmC p-value < 0.005. (**D**) Relative genomic 5hmC content compared with 5hmC content in origins that have fired (nascent strand); 5hmC p-value < 0.001. (**B–D**) Graphs represent median with minimum and maximum value; two biological replicates included. (**E**,**F**) Heatmaps illustrating 5mC (**E**) and 5hmC (**F**) signal around replication origins. The distance (kb) from the origin of replication is indicated on the x-axis. Colors correspond to the log2 value of data points at a given position: in general, occupancy is indicated in yellow and depletion indicated in blue.

Our top hit observed on the 5hmC binding protein gene ontology over-representation test was “DNA duplex unwinding” including the MCM3 protein. Since MCM3 is a subunit of the hexameric MCM2-7 helicase, we speculated that all components of this helicase would interact more strongly with 5hmC-modified DNA. While only MCM3 was observed in our initial screen, we found that all 6 subunits of the replicative helicase, MCM2-7, interacted with 5hmC-modified DNA. We were unable to detect an interaction between any of the MCM2-7 subunits and unmodified DNA (Fig. 2A). In order to validate the pull-down efficiency, a positive control previously identified by mass spectrometry was included. As expected, the DNA mismatch repair protein MSH2, was shown to bind both unmodified and 5hmC-modified DNA (Fig. 2A).

As compared with input, DNA immunoprecipitated from mouse embryonic stem (mES) cells with MCM2 antiserum showed a greater than 5-fold enrichment of 5hmC, suggesting that 5hmC and the MCM2-7 helicase interact *in vivo* (Fig. 2B). In addition, we show that all MCM2-7 subunits co-immunoprecipitate with DNA enriched with 5hmC; however, only MCM2, MCM4 and MCM6 showed a statistically significant increase in 5hmC compared with the input control (Supplemental Fig. S6A). In contrast, DNA that co-immunoprecipitates with MCM4 is also enriched with 5mC (Supplemental Fig. 6B).

### Origins are enriched for 5hmC, while active origins are 5hmC depleted

The MCM2-7 helicase is not only a replicative helicase but it is also the principal component in replication licensing, ensuring that the genome replicates once and only once. MCM2-7 helicase is present not only at active origins but also at inactive origins. Based on the preferential binding of MCM2-7 to 5hmC-modified DNA, we hypothesized that 5hmC was present at both inactive and active origins. As we have shown, 5hmC is enriched at DNA regions occupied by MCM2 (Fig. 2B), demonstrating that 5hmC occupies origins *in vivo*. This result was verified by intersecting 5hmC-enriched genomic regions with origin of replication sequences^25,58^ (data sets are described in materials and methods) (Fig. 2C). Further clarifying this finding, we included heat maps illustrating 5mC and 5hmC signal around replication origins (Fig. 2E,F)^25,58^. Figure 2F supports the hypothesis that 5hmC co-occurs with selected replication origins. Recently fired origins of replication can be identified by the incorporation of BrdU into the nascent strand followed by a BrdU pull-down of size fractionated nascent strand (Supplemental Fig. S7)^25^. Using this method, we observed a strong depletion of 5hmC at nascent strand as measured by mass spectrometry (Fig. 2D). At the same time, 5mC was slightly and non-significantly reduced. Our mass spectrometry results also indicate that the newly replicated DNA, which contains a fired origin of replication, is relatively GC rich (Supplemental Fig. S8A); the absolute 5mC and 5hmC base composition at origins are shown in Supplemental Fig. S8B,C. These results suggest that (i) 5hmC is globally enriched at replication origins and (ii) that 5hmC is depleted at fired origins.

### 5hmC content is proportional to doubling time

**Figure 3:**
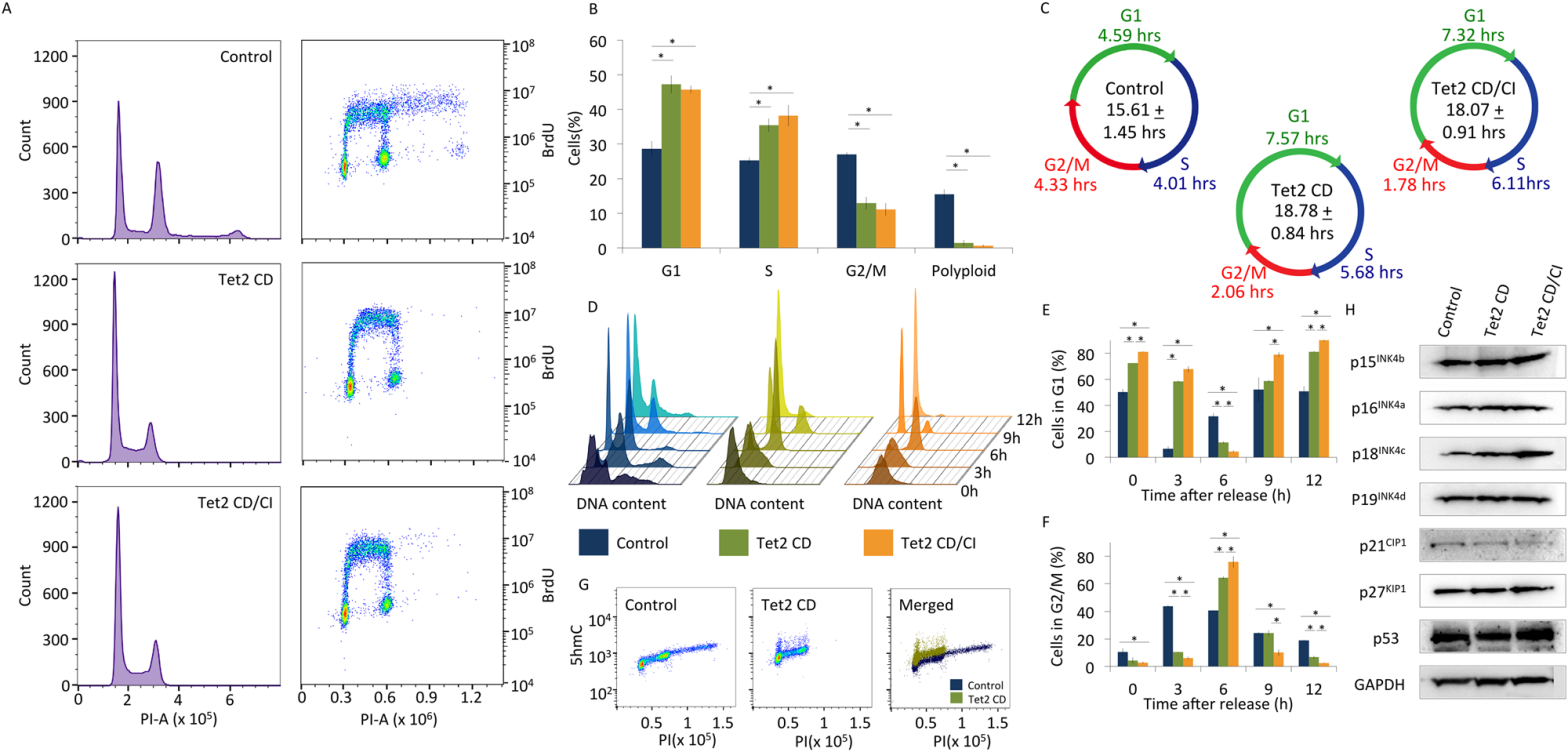
Genomic 5-hydroxymethylcytosine increases cell doubling time by inducing G1 delay via a novel mechanism. (**A**) Stable HeLa cell lines overexpressing a control YFP plasmid (Control), the Tet2 catalytic domain YFP fusion (Tet2 CD), or the inactive mutant of the Tet2 catalytic domain fused to YFP (Tet2 CD/CI) were incubated with BrdU, antibody stained for BrdU and propidium iodide and subjected to flow cytometric analysis. (**B**) Analysis showing proportions of cells in G1, S, or G2/M phases; (**C**) Total cell cycle time of each population of cells and the time spent in G1, S, or G2/M phases. (**D**) Flow cytometer scans of cells released from a double thymidine block at the indicated times. One representative synchronization is shown for each cell populations; (**E**) Quantification of cells remaining in G1 phase at each time point; (**F**) percentage of cells in G2/M phase after release from a double thymidine block;. (**G**) Analysis of 5hmC levels of Control and Tet2 CD HeLa cell lines throughout asynchronous cell cycle. (**H**) Western blots depicting expression of cyclin dependent kinase inhibitors (p15^INK4b^, p16^INK4a^, p18^INK4c^, p19^INK4d^, p21^CIP1^, p27^KIP1^), p53, and GAPDH in Control, Tet2 CD, and Tet2 CD/CI stable HeLa cell lines. Data shown are means of three biological replicates and are represented as standard deviation from the mean.

Since we found that 5hmC interacts with several components of the replication licensing machinery and that 5hmC is present at origins of replication, we hypothesized that 5hmC may have an effect on the cell cycle. We evaluated this hypothesis by creating HeLa cell lines that stably express either YFP (Control), a Tet2 CD:YFP fusion (Tet2 CD), or a Tet2 CD:YFP fusion mutant (Tet2 CD H1295Y/D1297A) that has been previously reported^59,60^ to be catalytically inactive (Tet2 CD/CI). The Tet2 CD and Tet2 CD/CI cell lines express more exogenous Tet2 CD as shown in Supplemental Fig. S9A. As expected the Tet2 CD stable HeLa cell line has a significant increase in 5hmC compared to the control cell line (Supplemental Fig. S9B). Surprisingly, we found that the HeLa cell line expressing the catalytically inactive Tet2 CD mutant also had increased cellular 5hmC content (Supplemental Fig. S9B). This finding leads us to conclude that the Tet2 CD H1295Y/D1297A (Tet2 CD/CI) mutations do not completely abolish Tet activity but rather reduce the catalytic activity of Tet2. All cell lines maintain similar 5mC levels (Supplemental Fig. S9B). We measured the proliferative properties of each of these cell lines in both asynchronous and synchronized cultures. In asynchronous cultures, we found that the doubling time of HeLa cells expressing the Tet2 CD:YFP fusion protein was significantly longer (18.11 ± 1.34 hrs) than the control HeLa cell line (15.61 ± 1.45 hrs; p-value = 0.045). HeLa cell line expressing the Tet2 CD/CI mutant, which we show possesses catalytic activity, also demonstrated a cell cycle delay (18.78 ± 0.84 hrs; p-value = 0.009) (Fig. 3A–D).

As these cells that express either Tet2 CD or Tet2 CD/CI were dividing more slowly, we anticipated a delay in the cell cycle. Using flow cytometric analysis to compare DNA content (propidium iodide – PI) and recently synthesized DNA (BrdU), we were able to determine the fraction of cells in G1, S, or G2/M phases of the cell cycle (Fig. 3A). We found that cells expressing Tet2 CD were delayed in G1 phase (47.25 ± 2.56%) compared to control cell lines (28.67 ± 2.37%; p-value < 0.001) (Fig. 3B). While spending a similar, yet statistically different, amounts of time in S and G2/M phases of the cell cycle, cells expressing the Tet2 CD spent 7.57 ± 0.49 hrs in G1 phase, nearly twice as long as cells that only expressed the YFP control plasmid (4.59 ± 0.38 hrs; p-value < 0.001) (Fig. 3C). As the Tet2 CD/CI mutant expressed a partially functional copy of Tet2, we observed a similar delay in G1 phase (7.32 ± 0.24 hrs; p-value < 0.001) compared to the control. Values are summarized in Supplemental Table S5.

To confirm our observations, we synchronized cells in G1/S phase using a double thymidine block and in G2/M phase using a nocodazole block. Control cells released from a double thymidine block progressed to G2 phase more rapidly than cells that expressed either variant of Tet2 CD (Fig. 3D,E). These cells also showed a delay in reaching G2/M phase of the cell cycle (Fig. 3D,F). After the release from nocodazole block, cell population that expressed Tet2 CD were significantly delayed in G1 phase compared to the control population (Supplemental Fig. S10A,B). Through the cell cycle analysis of synchronized populations, we confirm that Tet2 CD expressing cells are delayed in G1 phase and that these cells have an overall cell cycle delay.

We were also interested in determining if 5hmC levels vary throughout the cell cycle. With this in mind, we evaluated the quantity of 5hmC in G1, S and G2/M phases in HeLa control cells and HeLa cells expressing Tet2 CD. Our results suggest that 5hmC quantity increases throughout the cell cycle (Fig. 3G). However, because the DNA content is also increasing throughout the cell cycle, these results indicate that 5hmC as a fraction of DNA content peaks in G1 phase of the cell cycle and is overall reduced as the cell progresses through S and G2/M phases (Data summarized in Supplemental Fig. S10C). This finding is consistent with the hypothesis that 5hmC is removed prior to or during origin firing.

We hypothesized that 5hmC-induced G1 delay could be mediated by the upregulation of cyclin dependent kinase inhibitors or p53. We found no evidence that p53 nor any cyclin dependent kinase inhibitor (p15^INK4b^, p16^INK4a^, p18^INK4c^, p19^INK4d^, p21^CIP1^, p27^KIP1^) was upregulated in the cell lines that overexpress Tet2 CD or Tet2 CD/CI (Fig. 3H). This result suggests that over-production of 5hmC causes a G1 delay through a mechanism that is unrelated to cyclin dependent kinase activity.

Taken together, these data suggest that 5hmC increases the time required for a cell to divide by retarding the cell cycle; the most pronounced delay from overabundance of Tet2 or 5hmC occurs in G1 phase of the cell cycle.

### MCM2 Chromatin Occupancy mirrors 5hmC levels throughout the cell cycle

The preferential binding of pre-replication complex to 5hmC-modified DNA together with cell cycle analysis let us to speculate that 5hmC directs the assembly of pre-replication complex. We expected that cells with elevated 5hmC levels would have increased chromatin occupancy of MCM2-7 in G1 phase of the cell cycle and at the same time, we would expect MCM2-7 chromatin occupancy to mirror 5hmC levels (Fig. 3G) in various cell cycle phases.

To measure MCM2 chromatin occupancy throughout the cell cycle, cell lines were fixed and permeabilized, followed by extraction of soluble proteins. Chromatin bound MCM2 was antibody-stained. Chromatin bound MCM2 in each phase of the cell cycle was evaluated using flow cytometry. Consistent with our hypothesis, MCM2 chromatin occupancy was increased in G1 phase and was decreased in G2/M phase (Supplemental Fig. S11A–C), and this result mirrors the 5hmC genomic content (Fig. 3G). As expected, MCM2 chromatin occupancy is increased in cell lines with elevated 5hmC levels. These findings are consistent with the hypothesis that 5hmC binds MCM2, prevents its release, and allows us to hypothesize that elevated 5hmC levels would retard the cell cycle by causing a G1 delay or arrest. In line with this hypothesis, an increase in genomic 5hmC levels could lead to their more prominent association with MCM2; this in turn will be reflected in G1 length.

### Elevated levels of 5hmC prevent rereplication

Consistent with previous studies, HeLa cells display high prevalence of polyploid cells^61^. Interestingly, during cell cycle analysis we observed that cells with elevated 5hmC levels have a significant reduction in the population of polyploid cells (Fig. 3A,B). This observation led us to speculate that Tet2 CD and/or 5hmC could be a barrier to genomic rereplication.

**Figure 4:**
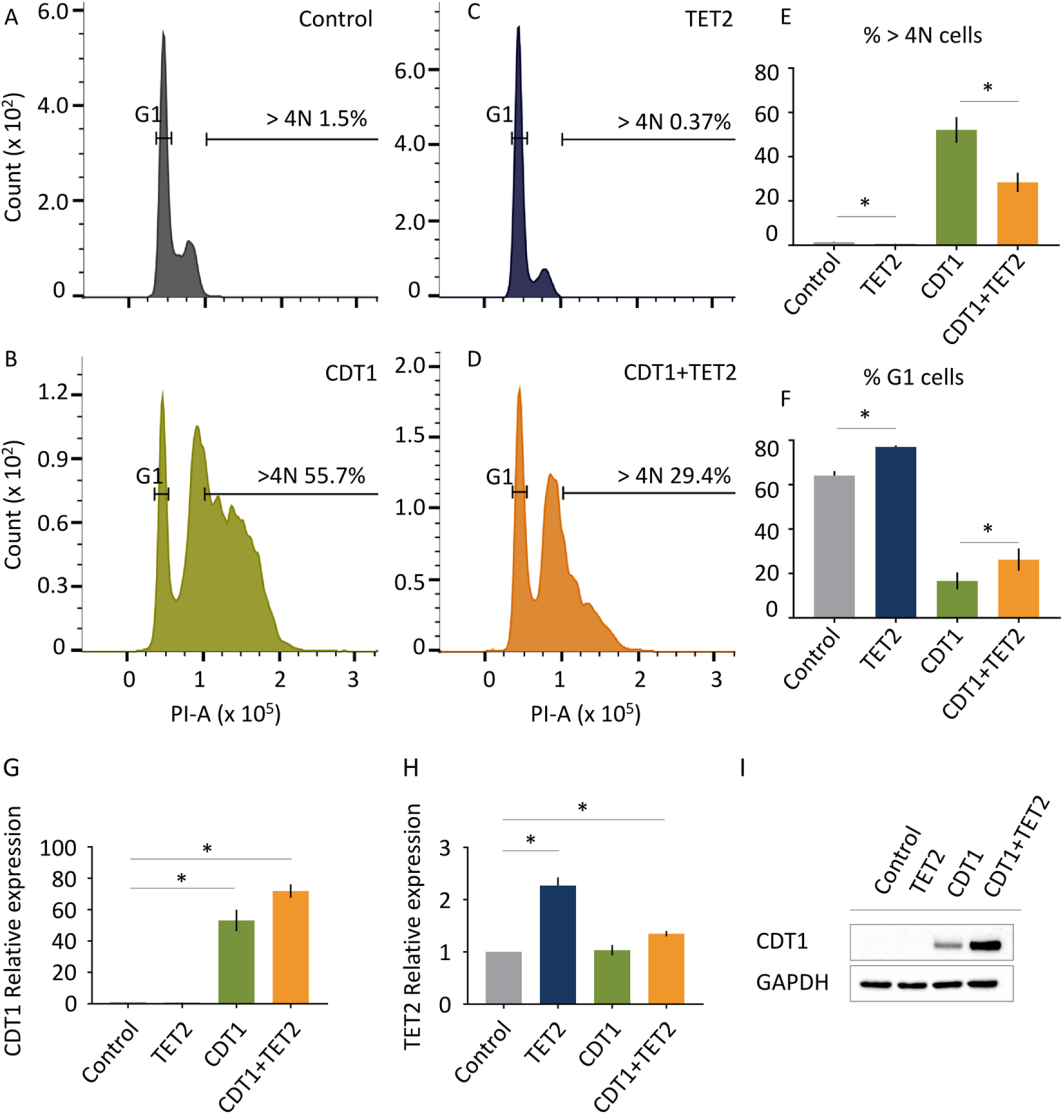
TET2 overexpression partially rescues rereplication phenotype induced by CDT1 overexpression. H1299 cells (**A**) were infected with adenoviruses encoding CDT1 (**B**), TET2 (**C**), and TET2 + CDT1 (**D**). Cells were stained with propidium iodide for DNA content and cell cycle phase identification. Cell cycle profile was analyzed by flow cytometry analysis 48 hours after adenovirus infection. Quantification of >4 N cells (**E**) and a G1 cells (**F**) expressed as percentage is indicated in corresponding panels. Relative mRNA expression: the 2− ΔΔCT method was used to analyze the relative changes in gene expression of CDT1 (**G**) and TET2 (**H**). (**I**) Western blot analysis of CDT1 levels upon infection with adenoviruses. GAPDH used as a loading control. (**A–D**) One representative cell cycle histogram is shown. (**E–H**) Data shown are means of three biological replicates and are represented as standard deviation from the mean. *p-value < 0.05.

It is well known that overexpression of CDT1 in p53-deficient cells results in substantial genomic rereplication^24^. We observed a similar effect in p53-deficient H1299 cells. In our hands, H1299 cells have a > 4n population of 1.5% (Fig. 4A) whereas when CDT1 is exogenously overexpressed 55.7% of cells are polyploid (Fig. 4B). The overexpression of the TET2 CD in H1299 cells resulted in a significant reduction of polyploidy; yielding a 4-fold decrease in polyploid cell population compared to the control cell line (Fig. 4A,C). TET2 CD is able to partially rescue the rereplication phenotype induced by CDT1 overexpression – 55.7% polyploid cells versus 29.4% when TET2 CD is co-expressed with CDT1 (Fig. 4B,D). As expected H1299 cells also experience a G1 delay when TET2 CD is overexpressed, supporting our previous results.

### High levels of 5hmC inversely correlate with organ proliferation rates

**Figure 5:**
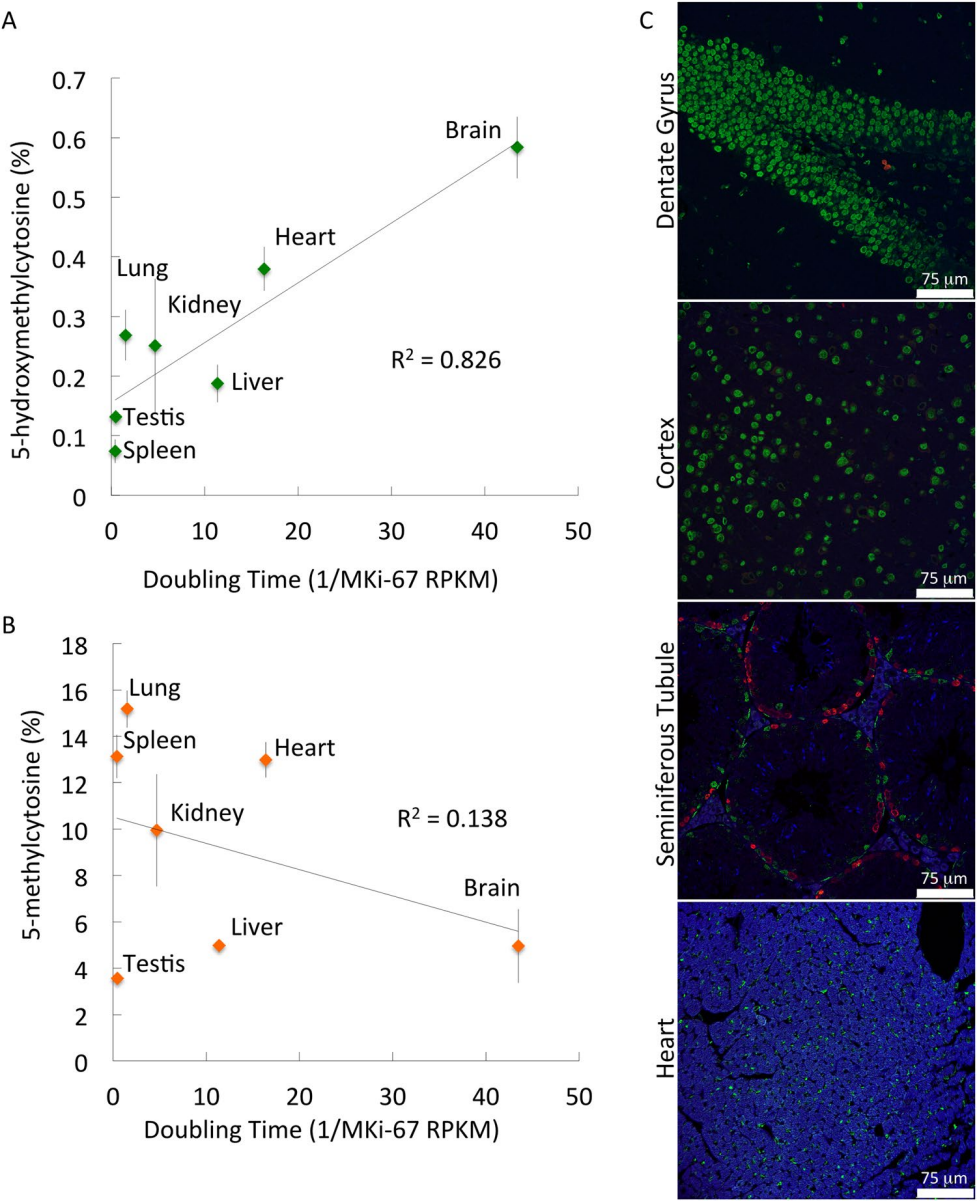
5-hydroxymethylcytosine levels strongly correlate with doubling time. Correlation between relative doubling time (1/MKi-67 RPKM) and (**A**) 5hmC or (**B**) 5mC levels in various organs. (**C**) Immunofluorescence of the dentate gyrus, cortex, testis and heart, comparing 5hmC levels (Green) with proliferation BrdU incorporation (Red). Numerical data consist of at least three technical replicates and at least two biological replicates.

As we found that elevated 5hmC levels delay progression through G1 phase thus reducing cell division, we wanted to see if this phenomenon was apparent also *in vivo*. We analyzed the 5hmC content in DNA isolated from mouse spleen, testis, liver, kidney, lung, heart and brain. We compared 5hmC levels with proliferation rate as measured by MKi-67 expression. We found a compelling linear relationship between doubling time (1/MKi-67 expression) and 5hmC levels (5hmC vs. 1/MKi-67 expression: R^2^ = 0.826; Spearman’s Rank Correlation Coefficient r_s_ = 0.857, p-value = 0.0137), showing that lower levels of 5hmC are indicative of increased proliferation (Fig. 5A). Our data suggest that 5mC content correlates poorly with doubling time (R^2^ = 0.138; Spearman’s Rank Correlation Coefficient, r_s_ = −0.286, p-value = 0.535) (Fig. 5B).

Indeed, the brain, composed of cells that proliferate relatively slowly, had the highest quantities of 5hmC (0.584 ± 0.051% 5hmC). Most cells in the brain are not actively dividing; according to our hypothesis, we expect high 5hmC levels in brain. Interestingly, some brain regions are actively dividing, which we expect to have low 5hmC levels. Consistent with our hypothesis, the neural progenitor cells in the dentate gyrus incorporated relatively high levels of BrdU but were almost devoid of 5hmC, while the non-proliferative neurons did not incorporate BrdU but had high 5hmC levels. Cortex sections composed of a homogenous population of non-dividing neurons showed a uniformly high level of 5hmC (Fig. 5C).

A similar situation was observed in the testis. Overall, testis DNA has low 5hmC levels (0.131 ± 0.007% 5hmC) and proliferates rapidly. Observation of testis sections revealed that slowly or non-dividing Sertoli cells contained high 5hmC levels, while the more rapidly dividing cells within the seminiferous tubules had low 5hmC levels (Fig. 5C).

DNA isolated from the slowly dividing heart tissue showed increased 5hmC levels (0.380 ± 0.037% 5hmC) and relatively low Ki-67 expression. Composed of slowly dividing cells, immunofluorescent images of heart sections showed homogenously high 5hmC levels (Fig. 5C).

These data strongly suggest rapidly dividing cells have low 5hmC content and non-dividing cells have elevated quantities of 5hmC.

## Discussion

Mammalian cells have multiple origins of replication. While each cell has many more origins of replication than required for accurate duplication of the genome, in healthy cells any given origin of replication should not initiate replication more than one time prior to division. Origin of replication firing more frequently would lead to polyploidy or other replication problems. To achieve a single replication event each active origin of replication becomes “licensed” for replication when certain factors are present at origins of replication. Among others, these factors include ORC1-6, CDT1, CDC6, and the MCM2-7 helicase. With some deviations, eukaryotic replication licensing is a highly conserved process. *S. cerevasiae* has a well-defined origin of replication sequence where replication licensing factors assemble. Interestingly, no mammalian origin of replication consensus sequence has been identified. Several groups have speculated that DNA methylation or histone modifications may signal the assembly of the replication licensing machinery^25,33,62–70^. Even if a limited relationship exists between DNA methylation or histone modifications and replication licensing, specific histone modifications or DNA methylation are not definitive of replication origins.

Our results suggest that 5hmC is a common feature of replication origins that may direct pre-RC assembly. We find that the licensing factor and replicative helicase MCM2-7 binds preferentially to 5hmC-modified residues rather than unmodified residues. 5hmC pull-down sequencing data intersected with origin of replication tiling arrays shows that 5hmC is a characteristic component of replication origins; then we suggest that 5hmC is removed prior to or during origin firing. In line with these findings, our data suggest that the presence of 5hmC is involved in cell cycle regulation and origin assembly. Indeed, cells that have the highest 5hmC levels are also proliferating the least. Consistent with this finding, we show that increased 5hmC levels slow the cell’s departure from G1. We demonstrate that 5hmC levels do not significantly vary throughout the cell cycle; origins are enriched for 5hmC and 5hmC is depleted at the fired origin. This led us to speculate that 5hmC must be removed from DNA prior to or during origin firing and is deposited immediately after newly replicated DNA is synthesized. We suggest that 5hmC is deposited rapidly on both mother and daughter strands after the replication fork passes beyond the origin of replication. We find this to be an attractive model since Tet1 was recently shown to interact with PCNA in the replication fork^71,72^. The notion that 5hmC is deposited after replication is consistent with our data and recent reports suggest that cellular 5hmC level related to DNA content (5hmC/total DNA) is slightly decreased^73^ throughout the cell cycle. The most striking evidence linking 5hmC to replication is our finding that TET2 CD overexpression strongly reduces the rereplication phenotype afforded by CDT1 overexpression.

Several groups provided evidence that supports a role for 5hmC in transcriptional regulation. These reports are not only compatible with our findings but also indirectly support our hypothesis, given that active origins of replication strongly correlated with genomic regions harboring active transcription. Moreover, we demonstrate that origins of replication have a genomic distribution overlapping with the one of 5hmC along the genome.

## Conclusions

**Figure 6:**
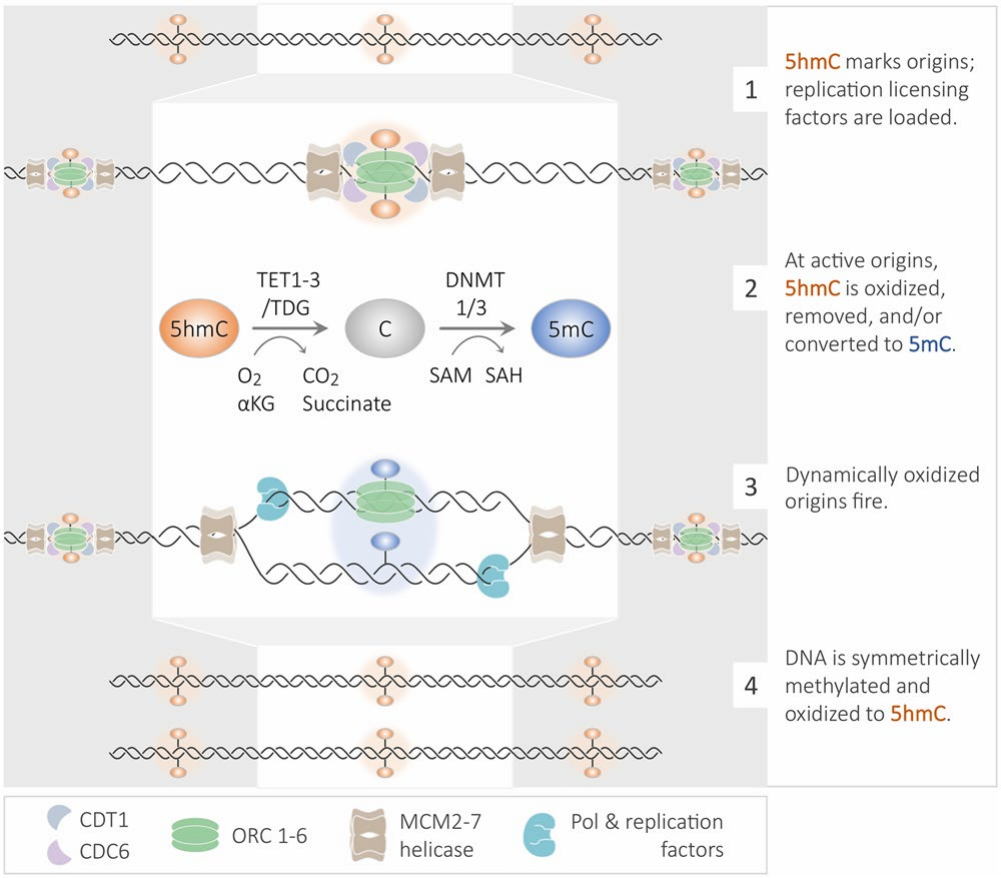
Model depicting 5-hydroxymethylcytsoine dynamics during origin firing. See text for details.

Consistent with our results and previous publications, we propose the following model – (1) 5hmC marks location of the origin of replication; in G1 phase, replication licensing factors are recruited to origins of replication, (2) as the cell progresses through S-phase, 5hmC is oxidized further, removed, or replaced with 5mC at active replication origins, (3) the origin fires, (4) 5hmC is deposited on the nascent and parental DNA strand (Model shown in Fig. 6).

As compared to the tissue of origin, cancer genomes have reduced 5hmC levels^74,75^. Furthermore, within any given cancer type the 5hmC levels have prognostic value^76–78^: cancers with higher 5hmC levels have better outcomes than cancers with low 5hmC levels. As cancer is broadly characterized by unrestricted cell growth and division, our model suggesting that 5hmC maintains cells in G1/G0, has potential utility in cancer diagnostics and prognostics. Our findings and the proposed model describe a general mechanism that explains both 5hmC levels and 5hmC genomic position.

## Methods

### Ethics statement

All animal experiments were approved and conducted in accordance with the national regulatory body – Federation for Laboratory Animal Science Associations (FELASA).

### Antibodies

Rabbit α-MCM2 antiserum (Flow cytometry 1:500; IF – 1:500; WB – 1:1000; ChIP – 1 μg; Abcam, cat. nr. ab4461), rabbit α-MCM3 antiserum (IF – 1:500; WB – 1:1000; Abcam, cat. nr. ab4460), Rabbit α-MCM4 antiserum (IF – 1:500; WB 1:1000; Abcam, cat. nr. ab4459), rabbit α -MCM5 antiserum (IF – 1:500; WB – 1:1000; Abcam, cat. nr. ab17967), Rabbit α -MCM6 antiserum (IF – 1:500; WB – 1:1000; Abcam, cat. nr. ab190948), rabbit α -MCM7 antiserum [EP1974Y] (IF – 1:500; WB – 1:1000; Abcam, cat. nr. ab52489), mouse α-5-hydroxymethylcytosine (5-hmC) antibody [GT513], (IF – 1:1000; Abcam, cat. nr. ab184148), goat α-mouse IgG H&L Alexa Fluor 594 (IF – 1:1000; Tissue IF – 1:1000; Abcam, cat. nr. ab150116), goat α-rabbit IgG H&L Alexa Fluor 488 (IF – 1:1000 Tissue IF – 1:1000; Abcam cat nr ab150077), rat α-BrdU antiserum (Flow cytometry – 1:100, Tissue IF – 1:100; Abcam, cat. nr. ab6326), donkey α-rat IgG Alexa Fluor 647 antiserum (Flow cytometry – 1:200; Abcam, cat. nr. ab150155), α-5hmC antiserum (Tissue IF – 1:100; Active Motiff, cat. nr. 39769), donkey α-rat IgG Alexa Fluor 594, (Tissue IF – 1:500; Thermofisher, cat. nr. A-21209), rabbit α-p15 INK4b (p15) antibody, (WB – 1:500; Abcam, cat. nr. ab53034), rabbit α-CDKN2A/p16INK4a antibody [EPR1473] (p16), (WB – 1:2000; Abcam, cat. nr. ab108349), rabbit α-p18 INK4c antibody [EPR15891] (p18), (WB – 1:1000; Abcam, cat. nr. ab192239), rabbit α-p19 INK4d antibody (p19), (WB – 1:100; Abcam, cat. nr. ab102842), rabbit α-p21 antibody [EPR18021] (p21), (WB – 1:1000; Abcam, cat. nr. ab188224), rabbit α-p27 KIP 1 antibody [Y236] (p27), (WB – 1:1000; Abcam, cat. nr. ab32034), rabbit α-p53 Antibody, (WB – 1:1000; Cell Signaling, cat. nr. 9282S), FLAG-Tag-HRP (WB – 1:2000; Sigma Aldrich, cat. nr. A8592-2MG), rabbit α-GAPDH antibody [GA1R], (WB – 1:1000; Abcam, cat. nr. ab125247), ECL Rabbit IgG HRP Linked whole Ab (WB – 1:10000; GE Healthcare, cat. nr. GENA934-1mL), ECL Mouse IgG HRP-linked whole Ab (WB – 1:10000; GE Healthcare, cat. nr. GENA931-1mL), Anti-phospho-Histone H3 (Ser10) (Flow cytometry – 1:300; Merck cat. nr. 05-806-AF647), goat α-rabbit dylight549 (Flow cytometry – 1:200; Vector Laboratories, USA cat. nr. DI-1549).

### Cell culture

HeLa cells were maintained in a 5% CO_2_, humidified, water-jacketed incubator at 37 °C. HeLa cells were grown in DMEM supplemented with 10% Fetal Bovine Serum, 2 mM glutaMAX, and 100 U/ml penicillin 100 U/ml streptomycin. Cell lines stably expressing YFP, Tet2 CD:YFP, and Tet2 CD/CI:YFP fusion were grown in the identical media supplemented with 1 mg/ml G418 (Constructs shown in Supplemental Table S6). Cells were passed as previously described^79^ between 70 and 90% confluence.

mES cells were maintained in 5% CO_2_, humidified, water-jacketed incubator at 37 °C. mES cells were grown in Knockout™ DMEM (cat. nr. 10829-018) supplemented with 15% Fetal Bovine Serum, 2 mM glutaMAX, and 100 U/ml penicillin 100 U/ml streptomycin, 1x NEAA, 0.1 mM ß-mercaptoethanol and 1500 U/ml LIF. Medium was changed every 24 hours and cells were typically passaged every 48 hr at a 1:20 ratio and seeded on CF-1 Irradiated MEFs (AMS Bio, cat. nr. GSC-6201G). MEFs were plated one day before on a plate coated by 0.1% porcine gelatin (SigmaAldrich, cat. nr. G1890).

Stem cells were grown in DMEM supplemented with 10% Fetal Bovine Serum, 2 mM glutaMAX, and 100 U/ml penicillin 100 U/ml streptomycin, 1x NEAA, 0.1 mM ß-mercaptoethanol and 1000 U/ml Leukemia Inhibitory Factor ((LIF) Merck Millipore, cat. nr. ESG1107).

### DNA substrates

Biotinylated DNA was created by the amplification of pUC19 DNA using the following primers: 5′GGTACCCGGGGATCCTCTAGAGT-3′ and 5′-biotin-ACTCTAGAGGATCCCCGGGTACC-3′. PCR reactions (50 μl) contained 200 μM dATP, 200 μM dGTP, 200 μM dTTP, and 200 μM of either dCTP or d5hmCTP, 50 ng pUC19, 1x Turbo DNA Polymerase Buffer, 1 M Betaine, 1 mM MgSO_4_, and 1 Unit of Turbo DNA Polymerase. Reactions were cycled 40 times in a thermocycler using the following program: 95 °C for 30 sec, 60 °C for 60 sec, 72 °C for 5 min. Substrates were purified by resolution on a 1.4% TBE Agarose Gel, followed by a QIAquick Gel Extraction (Qiagen, cat. nr. 28704). A complete sequence is shown in Supplemental Table S7.

### Pull down reactions

Biotinylated substrates were coupled to M-280 streptavidin coated superparamagnetic beads (KilobaseBINDER Kit, Cat nr. 60101, Life Technologies, Oslo, Norway). Briefly, 50 μg M-280 beads were prepared according to the manufacturer’s instructions. Washed beads and 1 μg of the appropriate DNA substrate were suspended in 20 μl binding solution and incubated with rotation at 25 °C for at least 3 hours. DNA:Bead complexes were washed twice in washing solution and twice in TBSTE (10 mM Tris pH 8.0, 100 mM NaCl, 2 mM EDTA pH 8.0, and 0.5% (v/v) Triton X-100). After washing, beads were incubated with 30 μg HeLa Nuclear Extracts (Promega cat. nr. E3091) in a final volume of 40 μl TBSTE with rotation for 20 min at 25 °C. Beads were washed twice with 200 μl TBSTE and suspended in 20 μl 1x LDS-Page loading dye (Life technologies, cat. nr. NP0007). Samples were boiled and resolved using SDS-PAGE. Samples were either analyzed by mass spectrometry or by Western analysis. Samples to be used in mass spectrometric analyses were resolved on a 4–20% acrylamide gel, while samples for Western analysis were resolved on a 10% acrylamide gel.

### Protein mass spectrometry

The gel resulting from 4–20% SDS-PAGE was fixed in a solution of 10% methanol/7% acetic acid for 30 minutes at 25 °C. The gel was then stained with SyproRuby Protein Stain (ThermoFisher cat. nr. S12000) at 25 °C overnight. The gel was washed in 10% methanol/7% acetic acid and imaged using a UV light source. Two bands resolved on 4–20% SDS-PAGE were excised from each lane. The bands contained proteins that encompassed the molecular weight of between 40 and 120 kDa. Peptides were identified using an electrospray ion trap mass spectrometer and matched to the relevant protein using a Mascot search. This search yielded two lists: (i) proteins that interact with unmodified DNA and (ii) proteins that interact with 5hmC modified DNA (Supplemental Table S1). Proteins that occur in both lists were removed from the 5hmC pull-down yielding proteins that interact 5hmC and not unmodified DNA. These 5hmC interacting proteins were then analyzed using a Panther Gene Ontology over-representation test. Statistically significantly over-represented biological functions are reported.

### Western blotting

Western blots were performed as previously described^80^.

### Chromatin immunoprecipitation

ChIP was performed as previously described^81^. Briefly, 5 × 10^7^ cells were fixed in 1% formaldehyde solution in PBS for 12 min at 25 °C. Glycine was added to a final concentration of 150 mM, followed by incubation for 5 min at 25 °C. Cells were centrifuged at 650 g for 10 min at 4 °C and washed twice in 25 ml of ice cold PBS. 170 µl of lysis buffer (50 mM Tris–HCl, pH 8.0, 10 mM EDTA, 1% (w/v) SDS, protease inhibitor mix (1:100 dilution from stock), 1 mM PMSF) was added to an aliquot of 7 × 10^6^ cells for 30 min on ice. Chromatin was sonicated using Bioruptor® Pico sonicator (Diagenode cat. nr. B01060001), settings: 15 cycles, 30 sec ON, 30 sec OFF. After sonication, samples were diluted with 10 volumes of RIPA buffer (10 mM Tris–HCl, pH 7.5, 140 mM NaCl, 1 mM EDTA, 0.5 mM EGTA, 1% (v/v) Triton X-100, 0.15% (w/v) SDS, 0.1% (w/v) Na-deoxycholate, protease inhibitor mix (1:100 dilution from stock), 1 mM PMSF). 1 ml of chromatin was incubated for 30 hours with Dynabeads protein A (Invitrogen, cat. nr. 100.02D) previously coupled overnight with 5 µg of α-MCM2 antiserum. After immunoprecipitation, beads were washed 3 times in RIPA buffer. Formaldehyde cross-links were reversed by eluting the samples in elution buffer (20 mM Tris–HCl, pH 7.5, 5 mM EDTA, 50 mM NaCl, 20 mM Na-butyrate, 1% (w/v) SDS, RNaseA 200 µg/ml) for 1 hour at 37 °C, adding 140 μg/mL proteinase K and incubating 4 hours at 68 °C. DNA was purified using UltraPure™ Phenol:Chloroform:Isoamyl Alcohol (25:24:1), EtOH precipitated and eluted in 11 µl of 10 mM Tris pH 7.5 (Qiagen, cat# 19086). Concentration was measured using Qubit® dsDNA HS Assay Kit (Thermofisher cat. nr. Q32851).

### 5hmC quantification

Quest 5-hmC DNA Elisa kit (Zymo Research, cat. nr. D5426) was used following the manufacturer’s instructions. Briefly, a 96 well plate was incubated with 1 ng/μl α-5-hydroxymethylcytosine antiserum in coating buffer for 1 hour at 37 °C. Wells were washed three times with 200 µl ELISA buffer, followed by blocking for 30 minutes at 37 °C with 200 µl of ELISA buffer. At the same time, genomic DNA (1 ng/μl) suspended in 100 µl ELISA buffer was heated to 98 °C for 5 min. Samples were immediately placed on the ice. DNA samples were than incubated in antibody-coated wells at 37 °C for 30 minutes. Wells were washed three times with ELISA buffer. α-ssDNA antiserum conjugated to HRP in ELISA Buffer (1:100 dilution) was incubated with each well for 30 min at 37 °C. Wells were washed three times with ELISA buffer and 100 µl of developer solution was added to each well; the color reaction proceeded for 30 min at 25 °C. Wells were analyzed for absorbance at 450 nm using a Wallac Victor^2^ plate reader.

### 5mC quantification

5mC DNA ELISA Kit (Zymo Research, Cat. nr. D5325) was used according to the manufacturer’s instructions to quantify 5mC levels. Briefly, DNA at 1 ng/µl in 100 µl 5mC coating buffer was heated to 98 °C for 5 min. DNA was immediately placed on ice for 10 min. DNA was added to the appropriate ELISA plate and incubated for 1 hour at 37 °C. Each well was washed three times with 5mC ELISA Buffer and blocked with 200 µl of 5mC ELISA Buffer for 30 min at 37 °C. Antibody mix consisting of α-5-Methylcytosine antiserum (1:200) and secondary antibody (1:1000) was prepared in 5mC ELISA Buffer. The antibody mix was added to the wells and incubated for 1 hour at 37 °C. Wells were washed three times with 5mC ELISA buffer. 100 µl of developer solution was added to each well; the color was developed for 30 min. Plate was read on a Wallac Victor^2^ plate reader at 450 nm.

### Quantification of DNA modifications using LC-MS/MS

DNA was hydrolyzed to deoxynucleosides by benzonase from *E. coli* (Santa Cruz Biotech), nuclease P1 from *P. citrinum* (Sigma), and alkaline phosphatase from *E. coli* (Sigma) in 10 mM ammonium acetate pH 6.0 and 1 mM magnesium chloride at 40 °C for 40 min, added 3 volumes of acetonitrile and centrifuged at 16,000 g for 30 min at 4 °C). The supernatants were dried and dissolved in 50 µl water for LC-MS/MS analysis of 5-hm(dC). A portion of each sample was diluted for the analysis of 5-methyl(dC) and unmodified deoxynucleosides. Chromatographic separation was performed using an Agilent 1290 Infinity II UHPLC system with an ZORBAX RRHD Eclipse Plus C18 150 × 2.1 mm ID (1.8 μm) column protected with an ZORBAX RRHD Eclipse Plus C18 5 × 2.1 mm ID (1.8 µm) guard column (Agilent). The mobile phase consisted of water and methanol (both added 0.1% formic acid). The following conditions were employed during analyses: for 5-hm(dC): 0.1 ml/min flow, starting with 5% methanol for 4 min, followed by 1-min gradient of 5–70% methanol, 5 min with 70% methanol, and 5 min re-equilibration with 5% methanol;forfor 5-methyl(dC) 0.25 ml/min flow, 3-min gradient of 5–90% methanol, followed by 4 min re-equilibration with 5% methanol; and for unmodified deoxynucleosides 0.25 ml/min flow, 20% methanol. Mass spectrometric detection was performed using an Agilent 6495 Triple Quadrupole system operating in positive electrospray ionization mode, monitoring the following mass transitions 258.1/142.1 (5-hm(dC)), 261.1/145.1 (D3 5-hm(dC), 242.1/126.1 (5-m(dC)), 252.1/136.1 (dA), 228.1/112.1 (dC), 268.1/152.1 (dG), and 243.1/127.1 (dT).

### Nascent strand isolation and BrdU pulldown

DNA and nascent strand were purified as described previously^25^ with slight modifications. Briefly, 1 × 10^8^ Dividing mES cells were pulsed with 100 µM BrdU for 60 min. Cells were washed with PBS and harvested in 20 ml of DNAzol (Invitrogen, cat. nr. 10503027) for 10 min at 25 °C. Samples were digested with 200 µg/ml Proteinase K (NEB, cat. nr. P8107S) at 37 °C overnight. After centrifugation at 4500 rpm at 4 °C for 15 min, the supernatant was added to a fresh tube and DNA was precipitated with the same volume of 100% EtOH for 1 hour at 25 °C. DNA was washed twice with 70% EtOH for 5 min and air-dried at 25 °C. DNA was suspended in 1 ml of TEN20 (10 mM Tris-Cl pH 7.9, 2 mM EDTA, 20 mM NaCl, 0.1% SDS, 1000 U RNasin) at 70 °C, denatured at 95 °C for 15 min and chilled on ice. Two aliquots composed of 500 µl denatured DNA was loaded onto a 10 ml, 5% to 30% sucrose gradient in TEN30 buffer (10 mM Tris pH 7.9, 2 mM EDTA pH 8.0, 300 mM NaCl) and centrifuged in a Beckman SW40 rotor at 21,600 RPM for 20 hrs at 4 °C. One milliliter fractions were withdrawn from the top of the gradient using a wide-bore pipette tip. Fifty microliters of each fraction was run on a 2% alkaline agarose gel at 30 V, overnight at 4 °C. The gel was neutralized in excess of neutralizing solution (1M Tris pH 7.6, 1.5 M NaCl) for 45 min 4 °C. The gel was stained with Ethidum Bromide (0.5 µg/ml) for 45 min at 25 °C. Prior to imaging, the gel was destained three times in ddH_2_O. Fractions corresponding to 0.5–2.5 kb were pooled and precipitated with 2.5 volumes ethanol and 0.3 M sodium acetate pH 5.5 for 2 hours at −80 °C. Pellets were washed twice with 1 mL of 70% ethanol and suspended in 1 mL of water. BrdU-IP was performed as previously described^82^ with the following adjustments. BrdU-labeled nascent strands were precipitated with 10 µl of monoclonal α-BrdU antibody (BD Biosciences, cat. nr. 555627) in IP buffer (10 mM Sodium Phosphate pH 7.0, 140 mM NaCl, 0.05% Triton X-100 in ddH_2_O) overnight at 4 °C. The following day, 30 µl of Dynabeads Protein G beads (Invitrogen, cat. nr. 10003D) were added to each sample and incubated 4 hours at 4 °C with rotation. Beads were washed twice for 5 min with 1 ml of IP buffer, and suspended in 200 µl of digestion buffer (50 mM Tris-HCl pH 8.0, 10 mM EDTA, 0.5% SDS in ddH2O) and incubated overnight at 37 °C with Proteinase K at a final concentration of 0.25 mg/ml. One hundred microliters digestion buffer were added to the samples and they were incubated for 2 hours at 68 °C. An organic extraction was performed and DNA was ethanol precipitated and dried.

### Bioinformatics analysis

Origin of replication (OoR) data is from a Nimblegen chip and in mm8 coordinates (http://genome.cshlp.org/content/21/9/1438.long) (GSM718735). We downloaded the processed data and the chip info and made a bed file with chromosomal coordinates and log2 ratio values. We then converted the mm8 coordinates to mm9 coordinates using UCSCs lift-over tool. We selected “significant” spots as those with a score > 0.5 (n = 18199).

5-mC and 5-hmC DIP-seq (GSE42250, GSE46111). mm9 coordinates. Supplementary_files_format_and_content: wiggle files (IgG signal subtracted; scores represent tag numbers per 10 million reads). We downloaded wig files and converted them to bed format and intersected these data with the significant OoR data. We also intersected 5-mC and 5-hmC data with random intervals (generated using bedtools shuffle on the significant OoR tiles).

We determined how many of the 5-mC and 5-hmC tile regions were enriched (IgG and depth normalized score > 5) and found no difference between OoR and random regions for 5-mC. In contrast, there was a substantial overrepresentation of tiles with enrichment for 5-hmC. We annotated the OoR regions and regions that were both OoR and enriched in 5-hmC using HOMER (v. v4.8.2) (cmd = annotatePeaks.pl OoR_pos_and_val_mm9.bed mm9).

Enrichment profiles of 5mC and 5hmC around ORI regions (Fig. 2E,F) were generated using ngs.plot v2.61 with the following options: -L 1 -P 32 -RR 10 -GO km. The data was then replotted using ggplot2 in R to view enrichment in only ORI regions. Following datasets were used: 5-mC and 5-hmC DIP-seq (GSE42250, GSE46111) Origin of replication (GSM718735).

### Growth curve

HeLa cells stably expressing YFP, Tet2 CD:YFP, or Tet2 CD/CI:YFP were seeded at 10^5^ cells/well density in a six well dish. At 24, 36, 48, and 60 hours after seeding, cells were harvested and counted using a Countess Cell counter (Life Technologies).

### Cell cycle and 5hmC analysis

HeLa cells stabling expressing YFP, Tet2 CD:YFP, or Tet2 CD/CI:YFP fusion proteins were incubated with 20 μM BrdU for 60 minutes. Cells were harvested, washed in PBS, and fixed in 1 ml of ice-cold methanol overnight at −20 °C on rotator. The following day cells were centrifuged at 1200 rpm for 4 min, the supernatant was removed and the cells were suspended in 1 ml 2 N HCl for 20 min at 25 °C. Cells were centrifuged at 1200 rpm for 4 min, the supernatant was discarded and cells were suspended in 1 ml of 100 mM Glycine in PBS for 20 min at 25 °C. Cells were centrifuged at 1200 rpm for 4 min, the supernatant was discarded and the cells were suspended in 1 ml 0.1% (v/v) Triton X-100 in PBS for 30 min at 25 °C. Cells were centrifuged at 1200 rpm for 4 min, the supernatant was discarded and cells were suspended in washing solution (PBS supplemented with 0.1% (v/v) Tween20 and 1% (v/v) goat serum) for 30 min at 25 °C. Cells were centrifuged at 1200 rpm for 4 min, the supernatant was discarded and cells were suspended in 100 μl PBS containing rat α-BrdU antiserum and incubated at 4 °C overnight with rotation. For 5hmC analysis, cells were incubated with 1:100 rabbit α-5hmC antiserum. The following day cells were washed three times with 1 ml washing solution for 5 min at 25 °C. Cells were suspended in 100 μl washing solution supplemented with goat α-rat IgG conjugated to Alexa Flour 647 for 2 hours at 4 °C with rotation. For 5hmC analysis, cells were incubated with 1:200 goat α-rabbit AlexaFluor 488. Cells were washed three times with 1 ml of washing solution at 25 °C for 5 min. Cells were suspended in 500 μl propidium iodide/RNase A solution and incubated for 30 min at 25 °C. Flow cytometric analysis of BrdU content was performed using BD Accuri C6 benchtop flow cytometer (BD biosciences) while 5hmC analysis during cell cycle using LSR Fortessa (BD Biosciences). Collected data was analyzed using FlowJo software (FlowJo LLC). Gating strategies are shown in Supplemental Fig. S12.

### Rereplication experiment

H1299 cells were maintained in a 5% CO_2_, humidified, water-jacketed incubator at 37 °C, grown in DMEM supplemented with 10% Fetal Bovine Serum and 100 U/ml penicillin 100 U/ml streptomycin. Infection with TET2-HA adenovirus (ABMgood, cat. nr. 138646A) was performed 12 hours after seeding 2 × 10^5^ cells/well in a 6-well plate with 1:1 ratio of medium and amplified virus. Cells were infected for 4 hours, after which the medium was changed for fresh one. 2 hours later, cells were infected with CDT1 Adenovirus (ABMgood, cat. nr. 079590A) for 4 hours with 1:1 ratio of medium and amplified virus. 48 hours later, cells were harvested and fixed with ice cold methanol. Cells were analyzed for cell cycle profile using DNA-stain Propidium Iodide (BioNordika cat. nr. 4087S). Expression of CDT1 was quantified with CDT1 Taqman probe (Thermofisher, cat. nr. Hs00153418_m1) and expression of TET2 with 5′-GCCCAATCCAGTTAGTCCTTATC-3′ and 5′-CAGCTTGAGATGAGGTGGAATAG-3′ primers. mRNA expression was normalized to housekeeping gene GAPDH (Taqman probe Thermofisher, cat. nr. Hs00153418_m1, 5′-GCGCCCAATACGACCAAAT-3′ and 5′-CCACATCGCTCAGACACCAT-3′). Cell cycle distribution was performed using flow cytometry on LSR Fortessa (BD Biosciences) and data were analyzed on FlowJo software. All experiments were performed in triplicate. Gating strategy shown in Supplemental Fig. S13.

### Double thymidine and nocodazole synchronization

HeLa cells were synchronized in G1-early S phase through double treatment with 2 mM thymidine (Sigma) for 14 h followed by releasing in fresh growth medium supplemented with 24 μM deoxycytidine (Sigma) for 9 h. Cells were collected at different time points after the second thymidine treatment and deoxycytidine release. For the synchronization from mitosis, double thymidine block was performed followed by a 2 h deoxycytidine release, then 100 ng/ml of nocodazole (Sigma) were added for 10 h. Mitotic HeLa cells were than shaken-off from the plate, washed, seeded and released in fresh medium and samples were collected at different time points. Cells were analyzed for cell cycle profile (DNA content) using DNA-stain Hoechst 33258 (1.5 μg/ml) and Anti-phospho-Histone H3 (Ser10) as a marker of cell cycle progression. Cell cycle distribution was performed using flow cytometry on LSRII flow cytometer (BD Biosciences) and data were analyzed on FlowJo software. All experiments were performed in duplicate. Gating Strategy of double thymidine block and nocodazole are shown in Supplemental Fig. S14A,B, respectively.

### MCM2 chromatin occupancy

In brief, the proteins that were not chromatin bound were extracted from the cell as previously described^83^. Cells were probed using anti-MCM2 antibody and dylight-549-goat-anti-rabbit secondary antibody (Catalog Number: DI-1549, VECTOR LABORATORIES, USA) and stained with DNA-stain Hoechst 33258 (1.5 μg/ml). The samples were analyzed on LSRII flow cytometer (BD Biosciences) using FlowJo software. Gating strategy is shown in Supplemental Fig. S15.

### *In Vivo* labeling with BrdU

0.3 mg BrdU dissolved in 0.9% ddH_2_O/g of mouse body weight was injected into the intraperitoneal cavity of 2-month old C57BL/6J male mice. 4 hours post-injection, mice were anesthetized by isofluorane followed by transcardial perfusion with 4% paraformaldehyde.

### Organ harvesting and sectioning

Organs fixed for 24 hours in 4% paraformaldehyde, were dehydrated overnight. Dehydrated organs were paraffin embedded and cut into 4 µm sections using an HM 355S Automatic Microtome.

### Immunohistochemistry

Sections were rehydrated using the following procedure at 25 °C: submerge twice in Clear rite 3 (Thermofisher Cat. nr. 6901TS) for 5 min, wash twice in 100% ethanol for 2 min, wash twice in 96% ethanol for 2 min, wash twice in 70% ethanol for 2 min, submerge in ddH_2_O for 5 min. Slides were boiled for 15 min in antigen retrieval buffer (10 mM Sodium Citrate, 0.05% Tween 20, pH 6.0), cooled and washed in PBS supplemented with 0.1% Tween 20 (PBST). Samples were denatured in 2 N HCl for 30 min at 37 °C. Samples were immediately neutralized in 0.1 M sodium borate for 10 min at 25 °C. Samples were washed three times in PBST for 5 min. Samples were blocked for 30 min in blocking buffer B (PBS, 0.1% Tween 20, 5% Bovine serum albumin, 5% goat serum, 0.5% TritonX-100) and incubated overnight with primary antibodies. The following day, slides were washed three times with PBST for 5 min. Slides were incubated with secondary antibodies in PBST for 1 hour at 25 °C. Slides were washed three times in PBST for 5 min and mounted with Vectashield containing DAPI (BioNordika, cat. nr. H-1200). Confocal images were obtained using a Leica SP8 confocal microscope equipped with 40x oil immersion lens and were processed using ImageJ^84^. Images were taken in the 1024 × 1024 pixel format with the zoom factor set at 1.72.

### Quantitative PCR

RNA from tissues was isolated using RNeasyMicro Kit (Qiagen, cat. nr. 74004) according to the manufacturer’s protocol. 500 ng of RNA was reverse transcribed into cDNA using QuantiTect reverse Transcription Kit (Qiagen, cat. nr. 205310) according to the manufacturer’s protocol. qPCR reactions (20 ul) contained Taqman PCR MasterMix (Thermofisher, cat. nr. 4352042), and a Taqman probe (Mouse Ki-67: Thermofisher, cat. Nr. 4331182; Mouse GAPDH: Thermofisher, cat. Nr. 4331182), and 20 ng cDNA. Real-time PCR was performed using StepOnePlus Real-Time PCR System (Applied Biosystems, USA) with the following cycling conditions: 95 °C for 10 min, 40 cycles at 95 °C for 15 sec and 60 °C for 1 min. MKi-67 expression from the GTEx expression database was validated using qPCR expression values (Supplemental Fig. S16). Samples contained at least three technical replicates and two biological replicates.

## Supplementary information

Supplementary Information

## Data Availability

All data generated or analyzed during this study are included in this published article.
