# Supplementary material for: 5-hydroxymethylcytosine Marks Mammalian Origins Acting as a Barrier to Replication
Source: Sci Rep. 2019 Jul 30;9:11065. doi: 10.1038/s41598-019-47528-3 (PMC6667497; doi:10.1038/s41598-019-47528-3)
Supplement: Supplementary file 1 — Supplementary Information [file 41598_2019_47528_MOESM1_ESM.pdf]

Supplemental Information

5-hydroxymethylcytosine Marks Mammalian Origins Acting as a Barrier to Replication

Terezia Prikrylova[1,#], Julia Robertson[1,#], Francesca Ferrucci[1], Dorota Konorska[1], Håvard Aanes[1], Adeel Manaf[1], Beibei Zhang[2], Cathrine Broberg Vågbø[3], Anna Kuśnierczyk[3], Karin M. Gilljam[1,3], Caroline Løvkvam-Køster[1], Marit Otterlei[1,3], John Arne Dahl[1], Jorrit Enserink[4,5,6], Arne Klungland[1,7] and Adam B. Robertson[1,*]

Index

Supplemental Figures (S1-S16)
Supplemental Figure Legends
Supplemental Gels (Full images of cropped gels found in the Figures)
Supplemental Tables (S1-S7)

Supplemental Figure S1

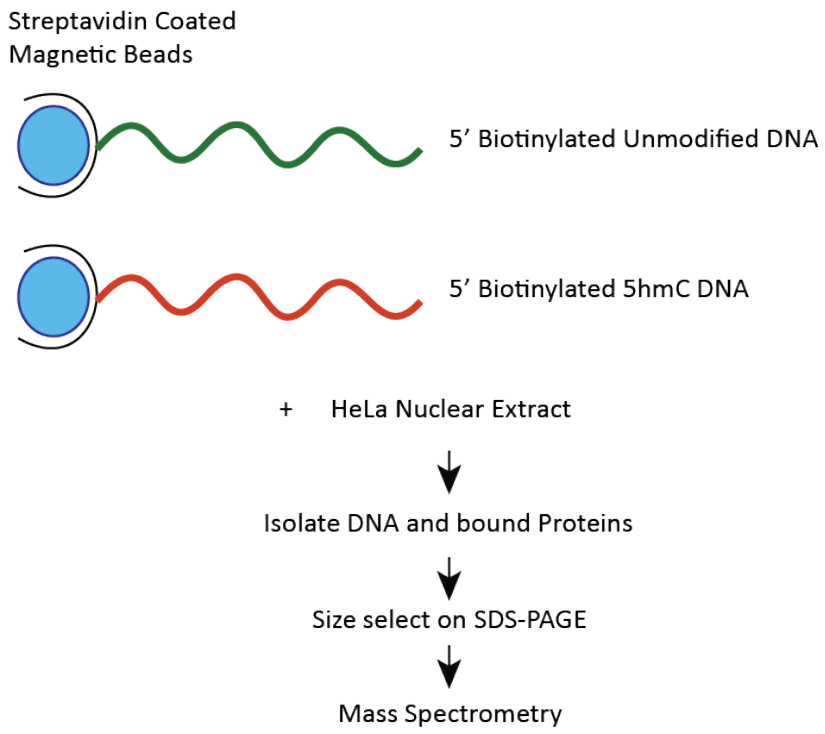

Supplemental Figure S2

A

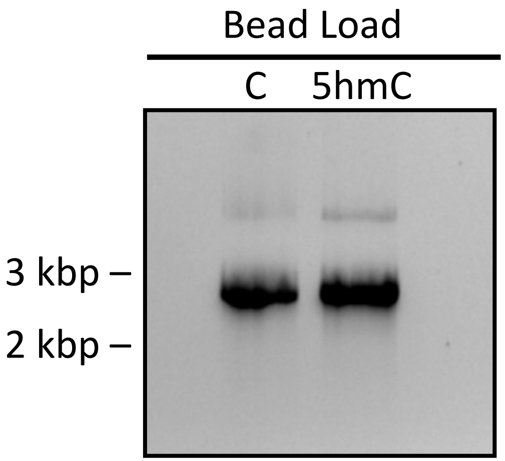

B

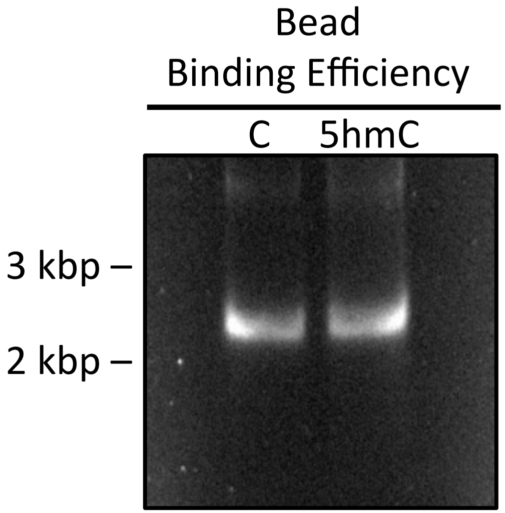

Supplemental Figure S3

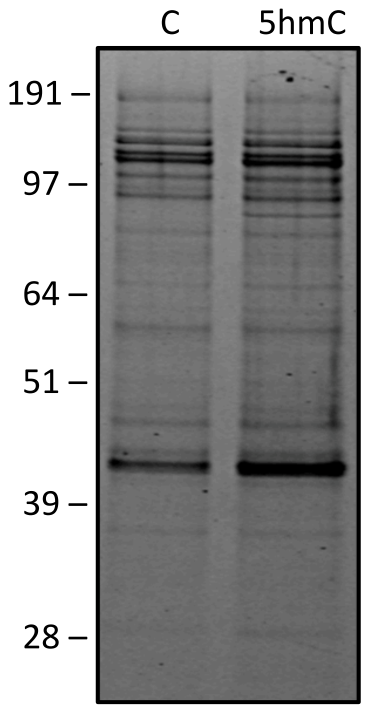

Supplemental Figure S4

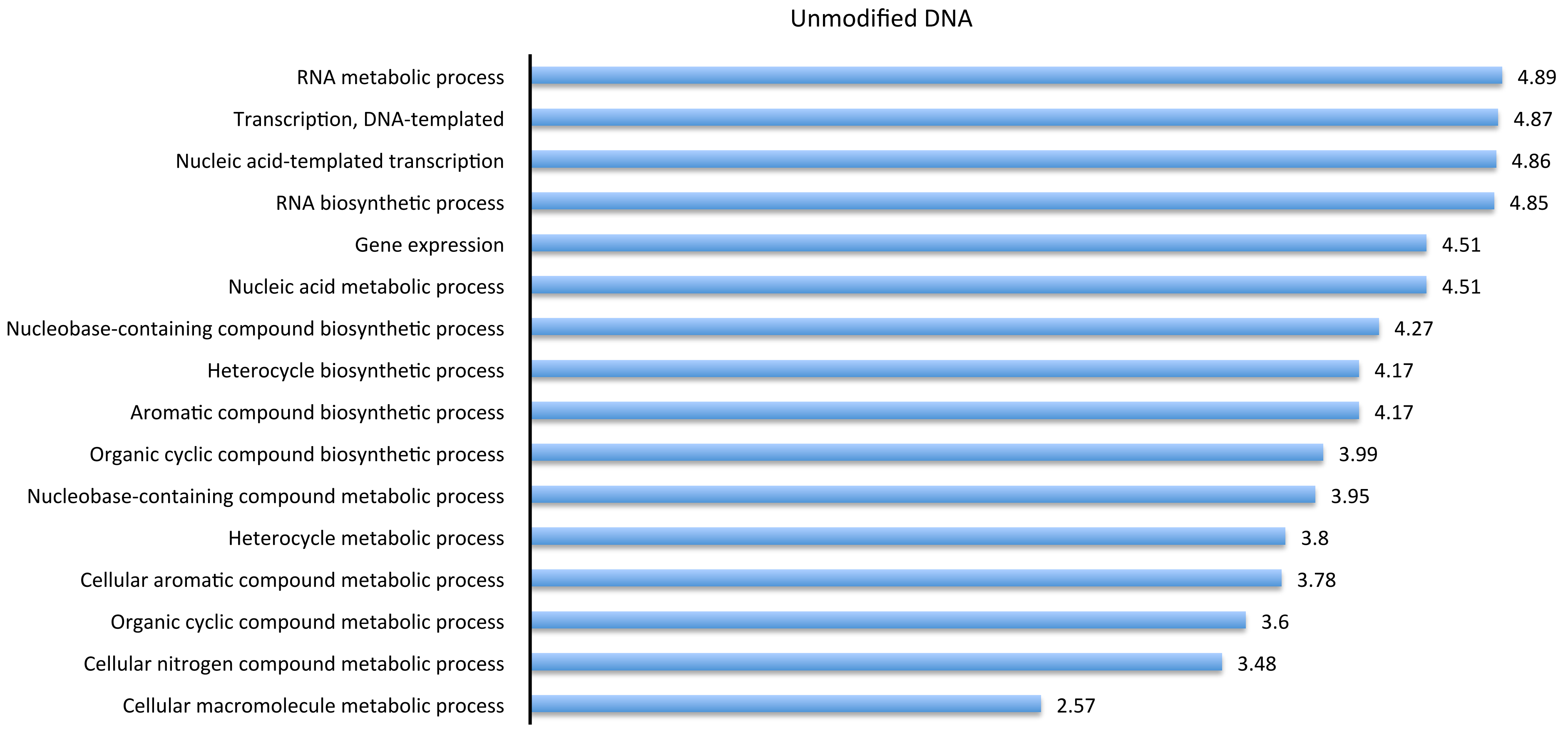

Supplemental Figure S5

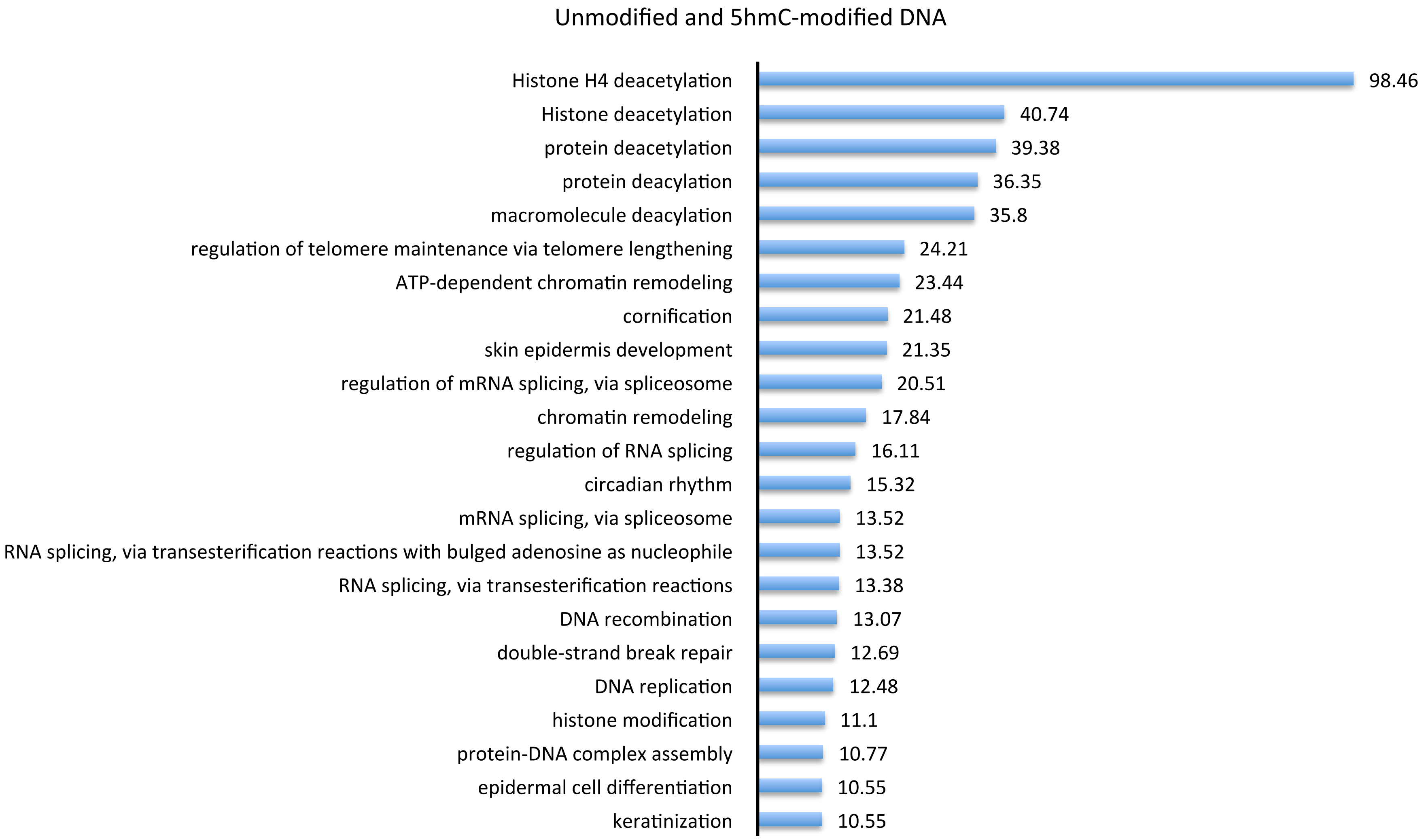

Supplemental Figure S6

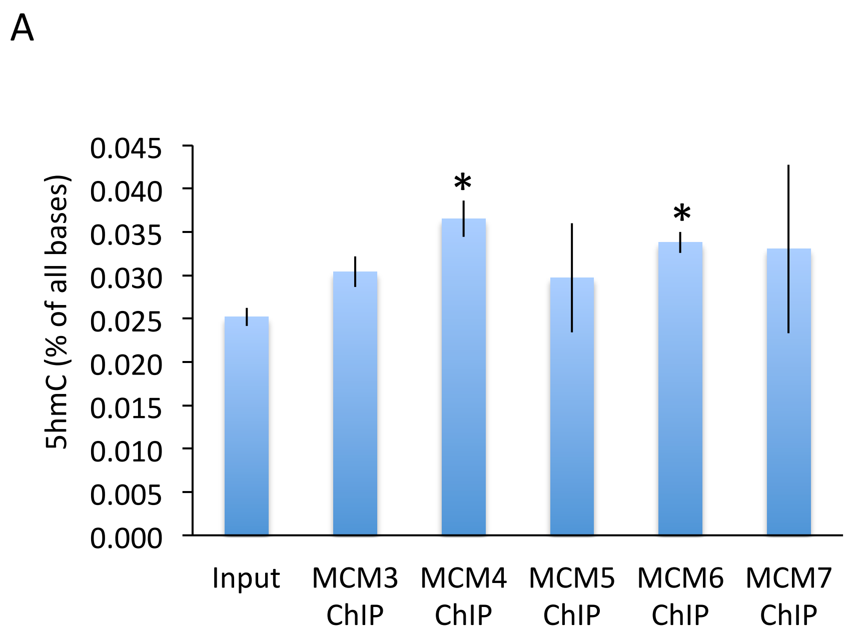

Supplemental Figure S7

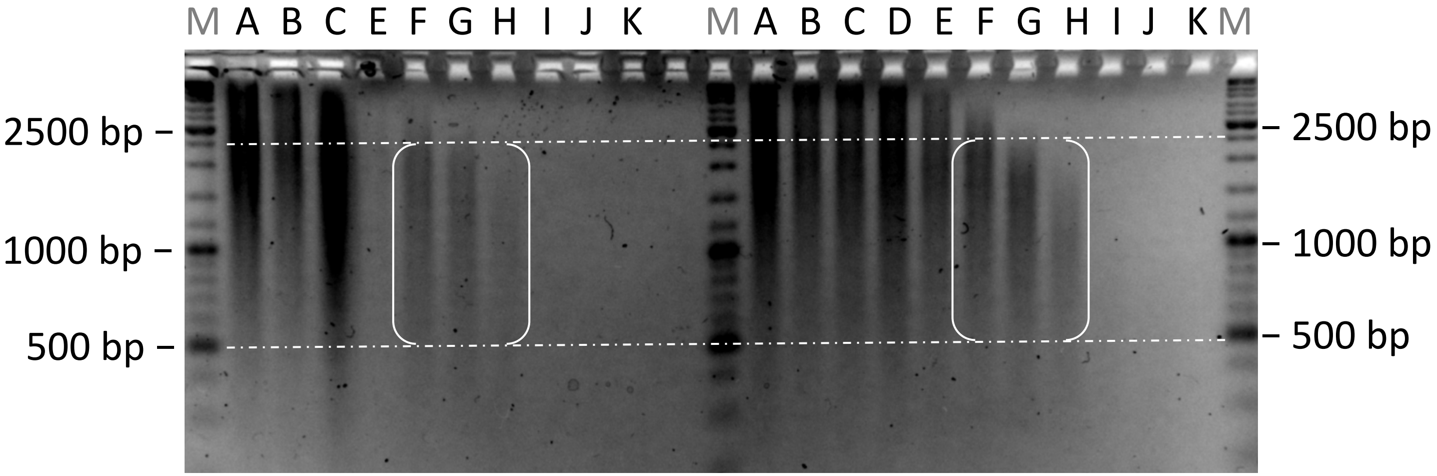

Supplemental Figure S8

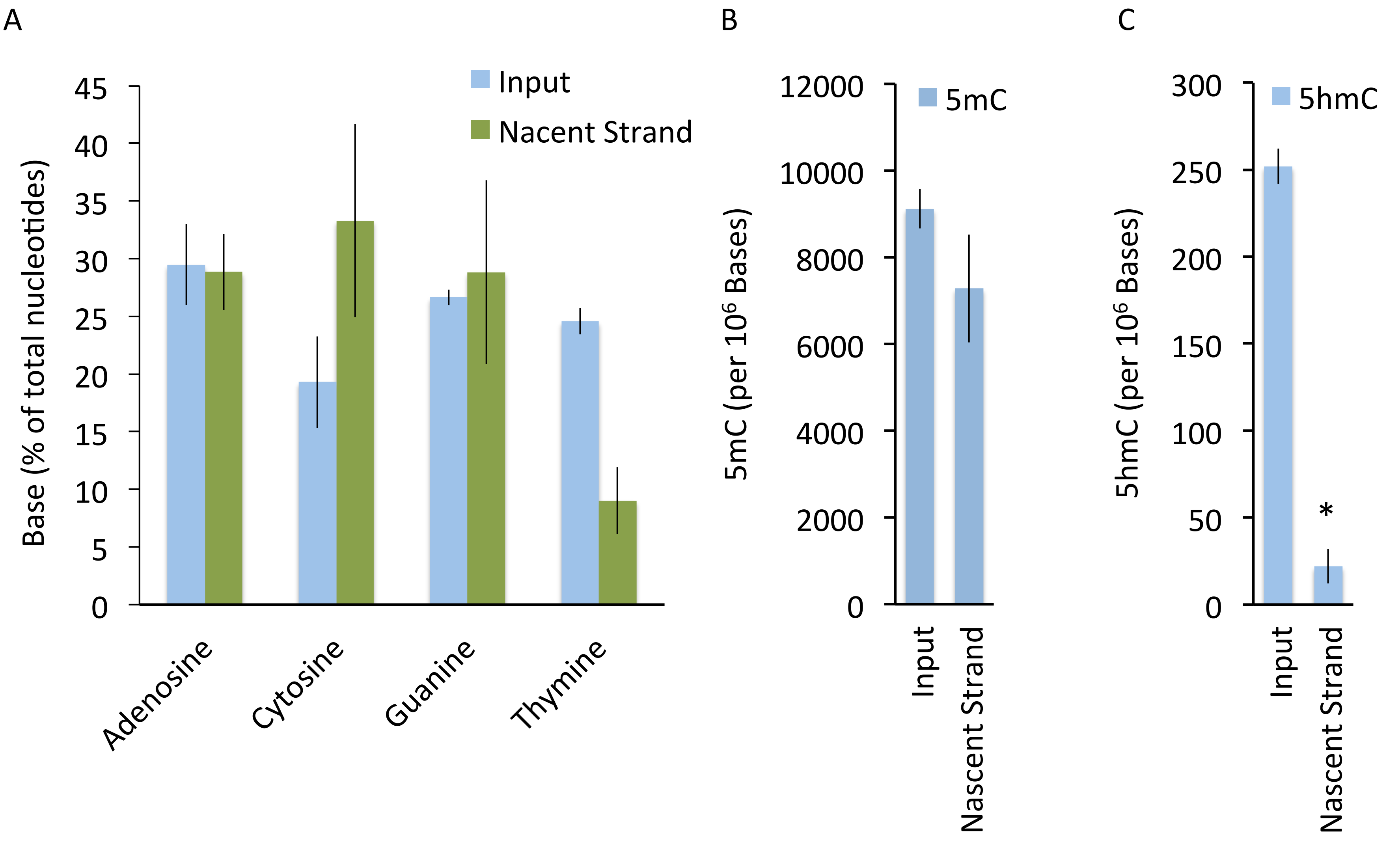

Supplemental Figure S9

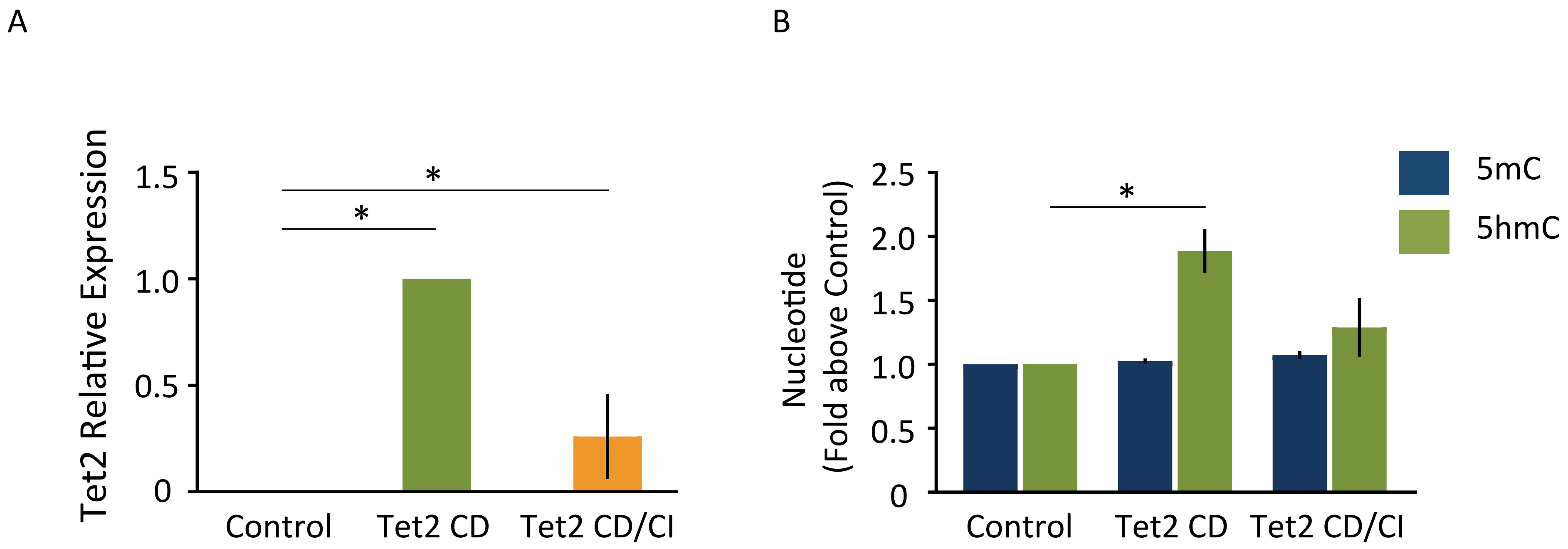

Supplemental Figure S10

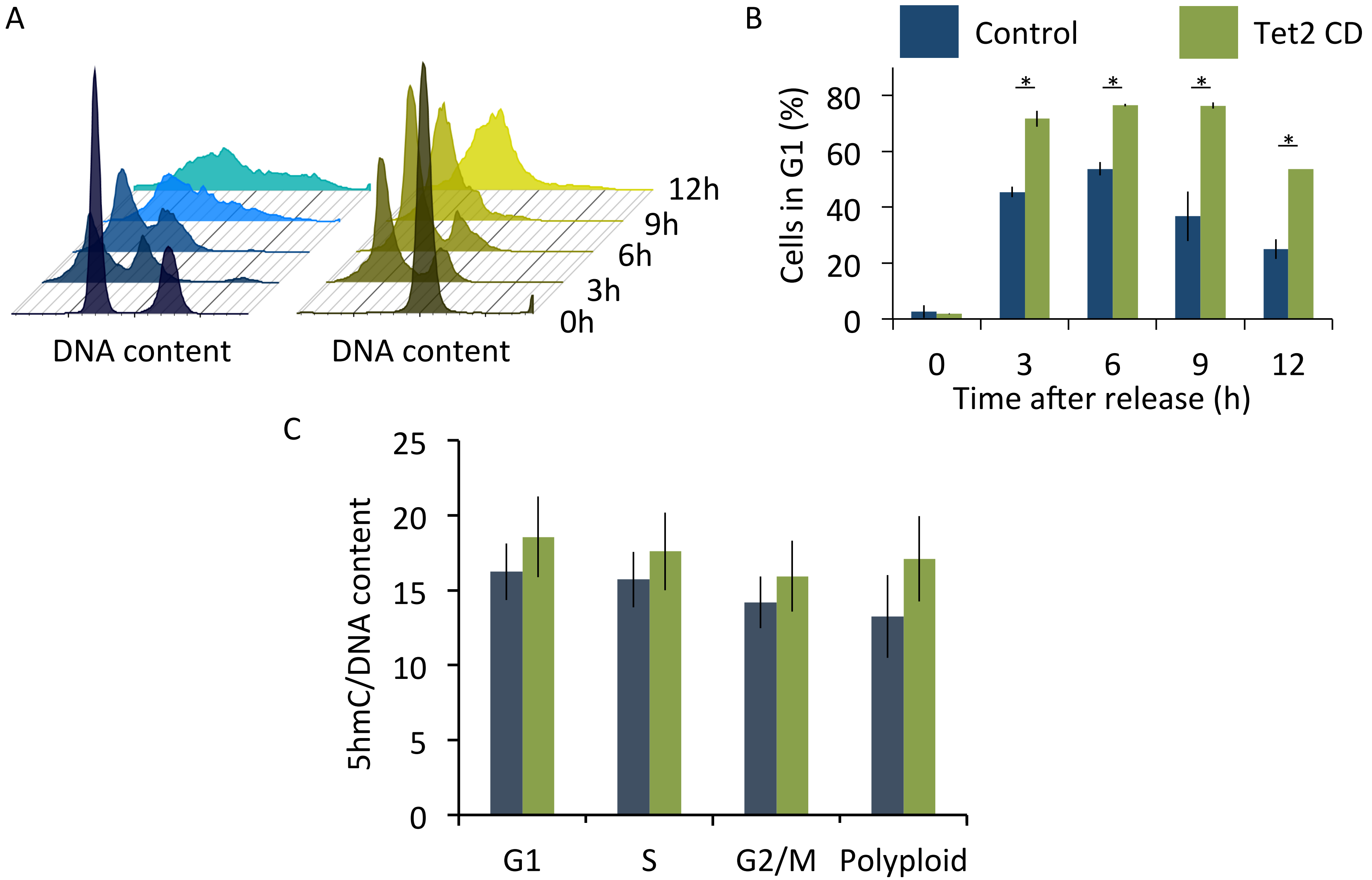

Supplemental Figure S11

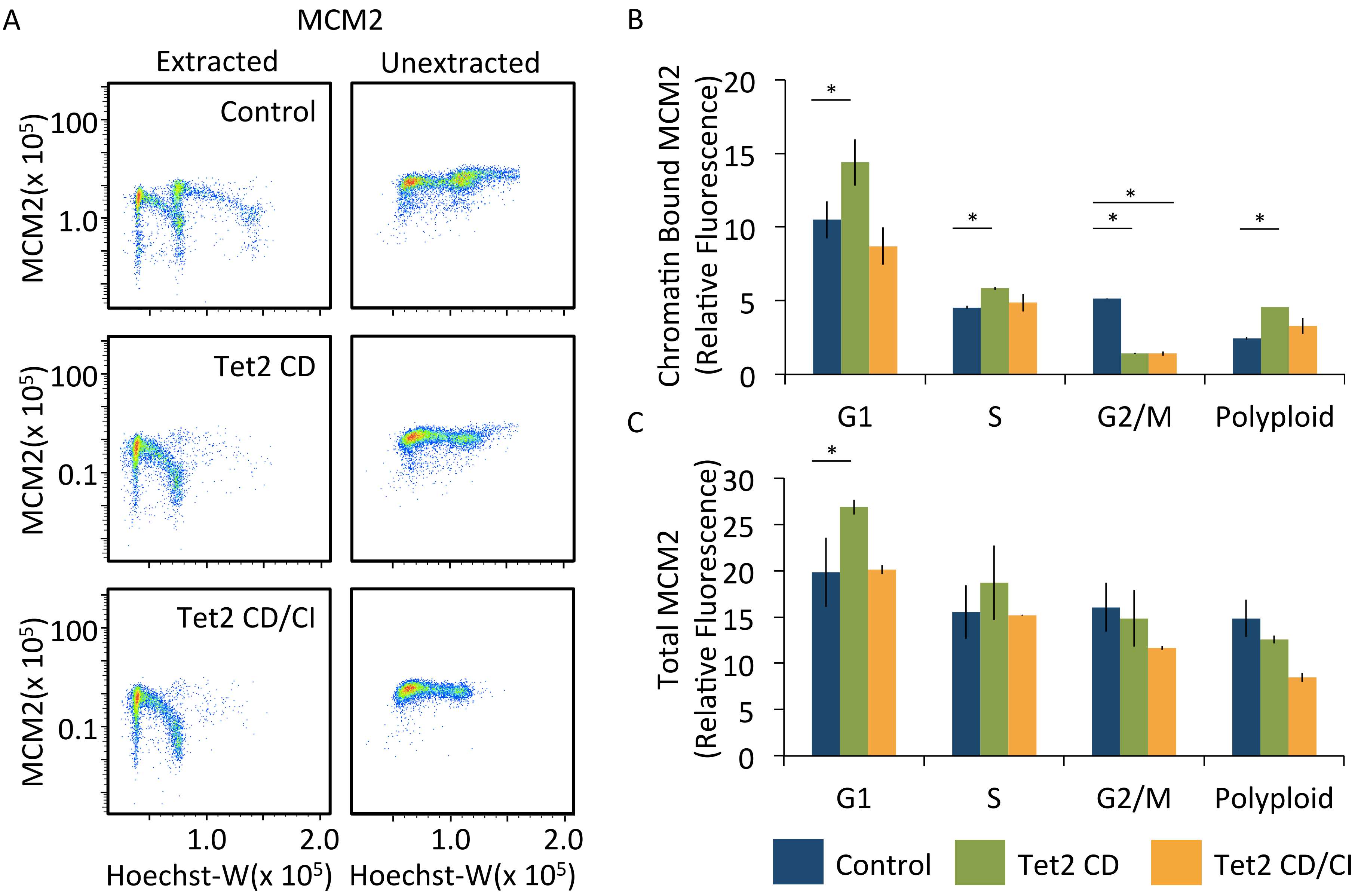

Supplemental Figure S12

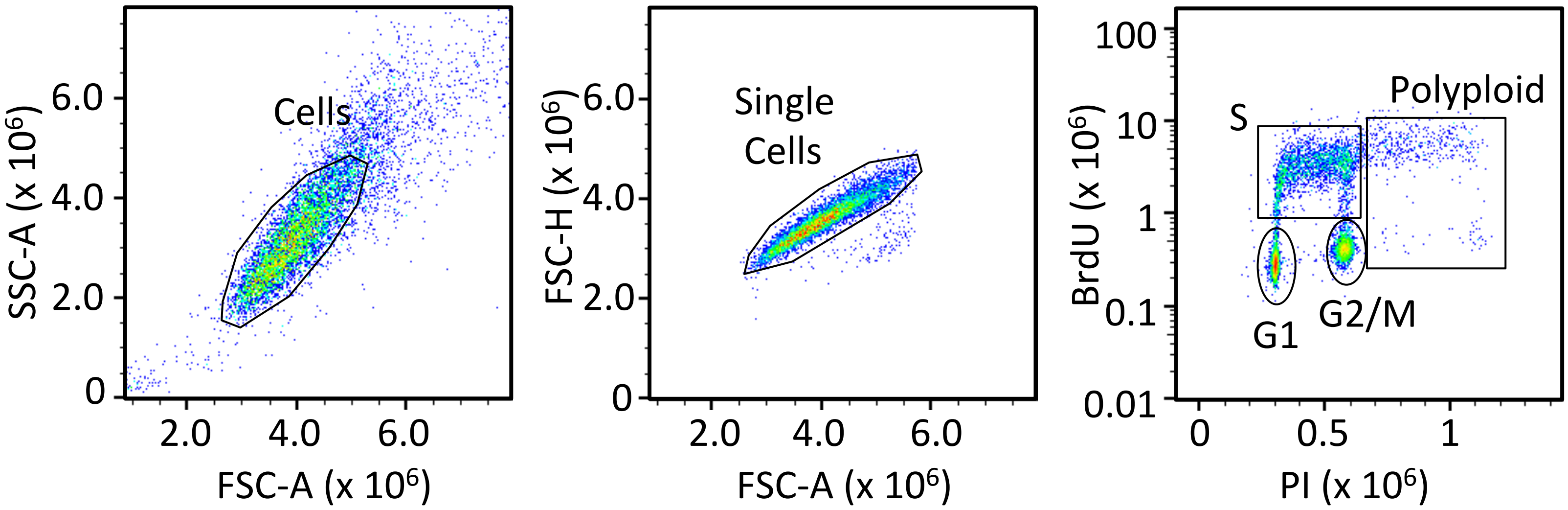

Supplemental Figure S13

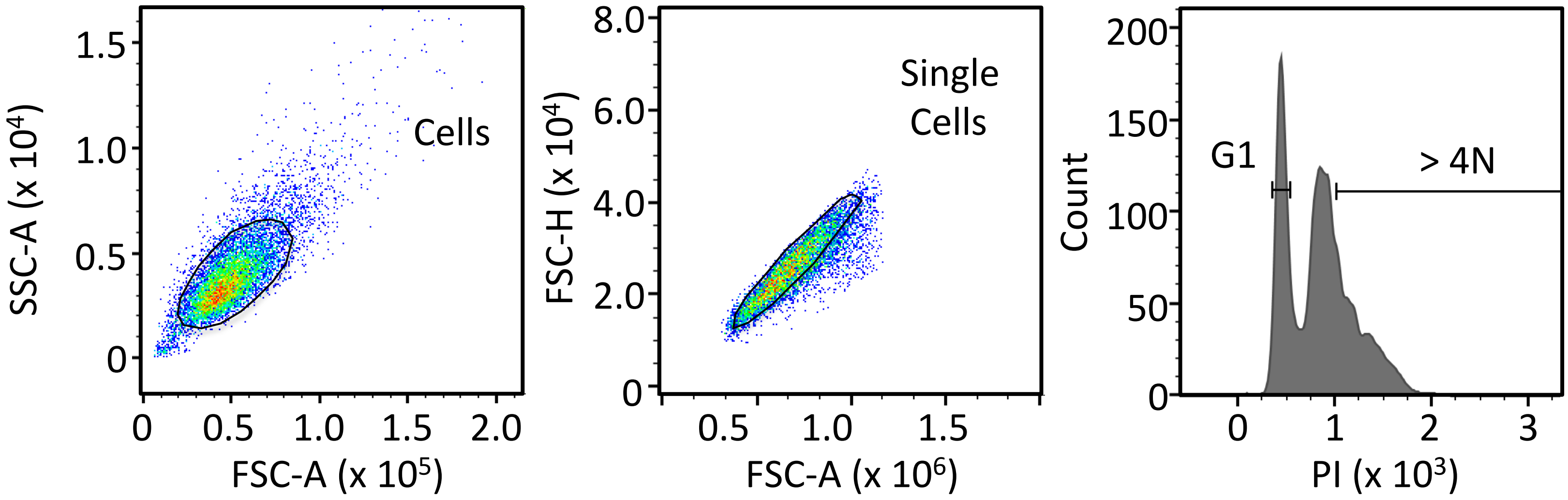

Supplemental Figure S14

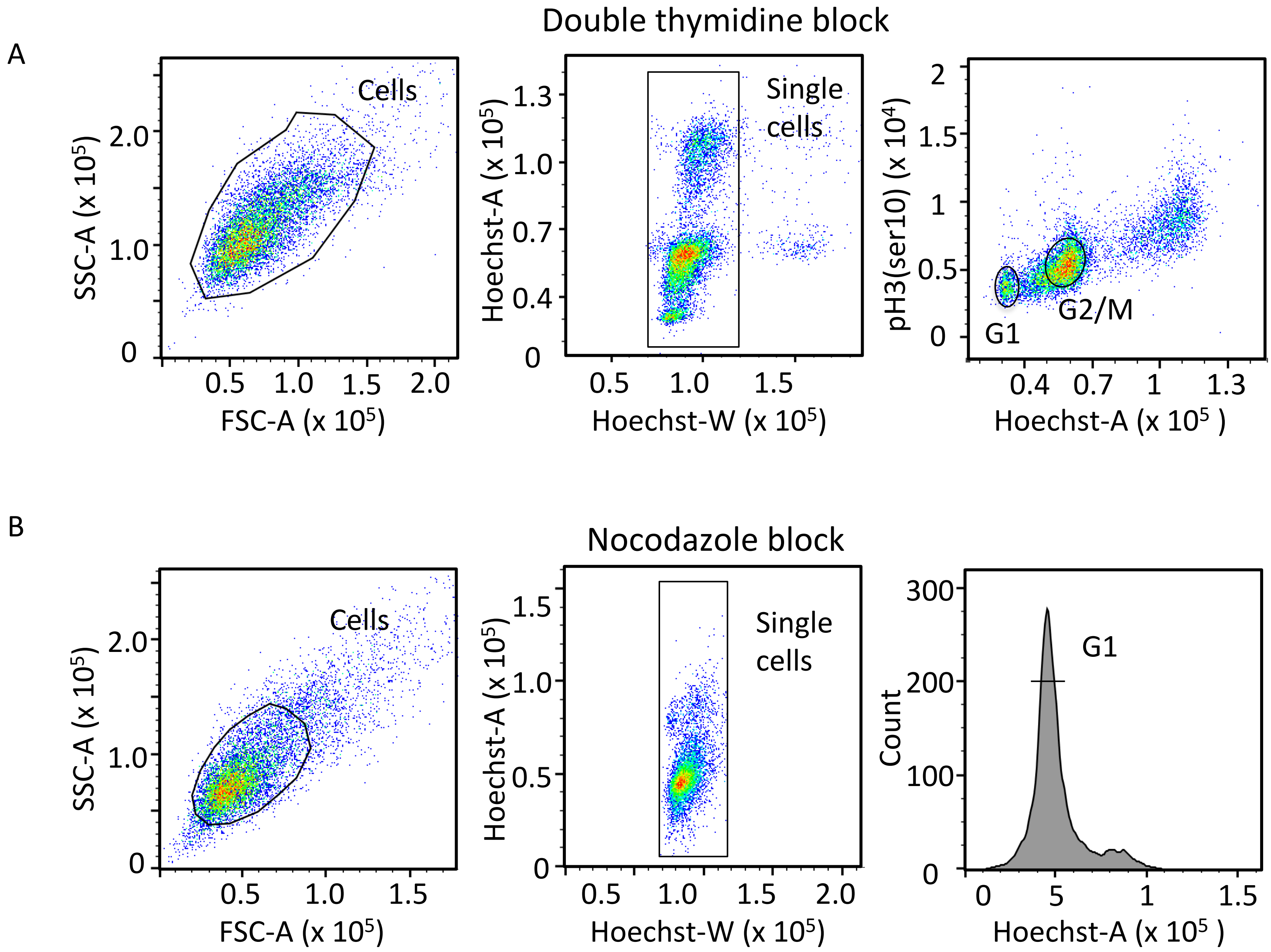

Supplemental Figure S15

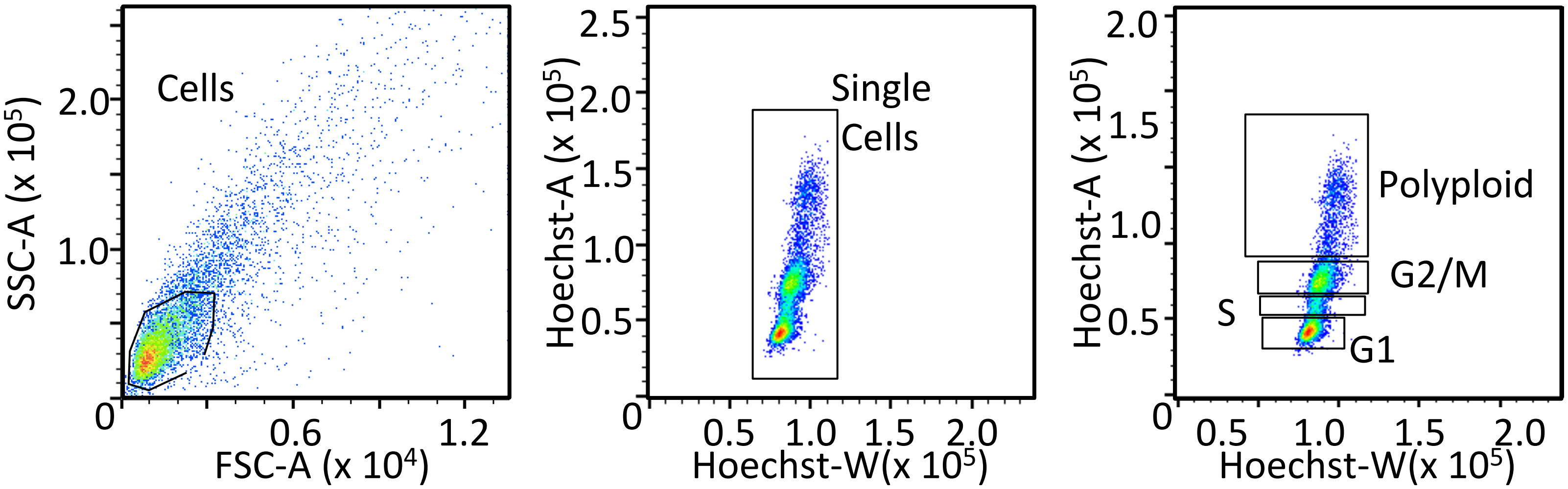

Supplemental Figure S16

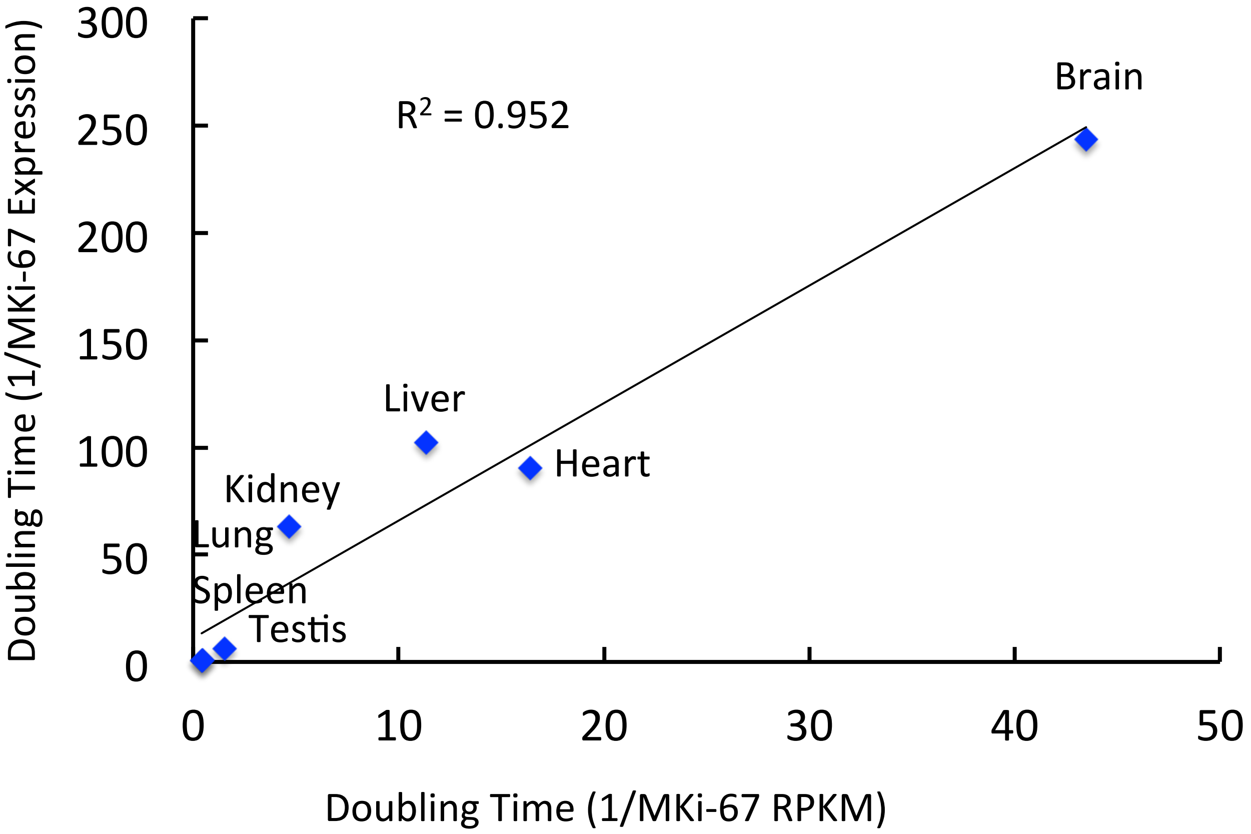

Supplemental Figure Legends

Supplemental Figure S1. 5hmC interaction experimental design. Unmodified and 5hmC-modified, biotinylated substrates were incubated with HeLa nuclear extracts as described in the "Methods" section. Proteins binding each substrate were resolved using SDS-PAGE and identified using Electrospray and MALDI mass spectrometry.

Supplemental Figure S2. Substrate loading and binding efficiencies are equivalent. Unmodified and 5hmC-modified DNA substrates incubated with HeLa nuclear extracts, are (**A**) loaded on magnetic beads in equivalent potions and (**B**) are recovered with similar efficiencies.

Supplemental Figure S3. Protein Identification. Proteins that bind to each substrate were resolved using SDS-PAGE and identified by mass spectrometry.

Supplemental Figure S4. Gene Ontology overrepresentation analysis of proteins that bind uniquely to unmodified DNA. Proteins that interacted exclusively with unmodified DNA were subjected to a Panther Gene Ontology over-representation test. Results of the over-representation test are displayed as fold above expected for a random protein population.

Supplemental Figure S5. Gene Ontology overrepresentation analysis of proteins that bind to both unmodified and 5hmC-modified DNA. Proteins that interacted with both unmodified and 5hmC-modified DNA were subjected to a Panther Gene

Ontology over-representation test. Results of the over-representation test are displayed as fold above expected for a random protein population.

Supplemental Figure S6. 5hmC (A) and 5mC (B) content in MCM immunoprecipitated mESC chromatin. * p-value < 0.05.

Supplemental Figure S7. Alkaline gel of nascent strand isolation. BrdU labeled DNA fractions from mESCs were loaded on 2% alkaline gel and fractions corresponding to 0.5–2.5 kb were pooled and used for BrdU immunoprecipitation.

Supplemental Figure S8. LC-MS/MS quantification of DNA bases (A) and DNA modifications 5mC (B) and 5hmC (C) in Input and Nascent strand of mESCs. * p-value < 0.001.

Supplemental Figure S9. Characterization of Stable HeLa Cell lines. (**A**) Tet2 expression and (**B**) 5mC and 5hmC content in HeLa cell lines stably expressing YFP (Control), Tet2 CD fused to YFP (Tet2 CD), and Tet2 CD inactive mutant (Tet2 CD/CI). * p-value < 0.05.

Supplemental Figure S10. Cell cycle synchronization of HeLa cell lines stably expressing YFP (Control) and Tet2 CD fused to YFP (Tet2 CD). Cells were synchronized in G2/M using double thymidine - nocodazole block and samples were analyzed at different time points after release (A). Percentage of cells in G1 phase

(%) after release (B). * p-value < 0.05. (C) 5hmC levels throughout the cell cycle. 5hmC levels normalized to mean intensity of fluorescence of PI.

Supplemental Figure S11. Flow cytometry analysis of MCM2 occupancy on DNA through cell cycle of HeLa cell lines stably expressing YFP (Control), Tet2 CD fused to YFP (Tet2 CD), and Tet2 CD inactive mutant (Tet2 CD/CI). (A) Flow cytometric analysis of Hela stable cell lines pre-extracted with salt and detergent before fixation (extracted) or fixed without pre-extraction (unextracted). Cells were stained with antibody against MCM2 and DNA-stain Hoechst. Density scatter plots are shown for MCM staining versus DNA content. (B) Relative fluorescence quantification of chromatin bound MCM2 normalized to mean intensity of fluorescence of PI (B) and total MCM2 normalized to mean intensity of fluorescence of PI (C) during cell cycle. * p-value < 0.05.

Supplemental Figure S12. Gating strategy for the flow cytometry analysis of HeLa cell cycle. Living cells were gated on a forward scatter (FSC)/side scatter (SSC) plot. Further, doublets were discriminated by plotting FSC-A versus FSC-H. Identification of individual phases of cell cycle was performed by visualizing DNA content (PI) versus BrdU incorporation.

Supplemental Figure S13. Gating strategy for the flow cytometry analysis of H1299 cell cycle. Living cells were gated on a forward scatter (FSC)/side scatter (SSC) plot. Further, doublets were discriminated by plotting FSC-A versus FSC-H. Identification of individual phases of cell cycle was performed by visualizing DNA content (PI).

Supplemental Figure S14. (A) Gating strategy for the flow cytometry analysis of cell cycle progression after double thymidine block. Living cells were gated on a forward scatter (FSC)/side scatter (SSC) plot. Further, doublets were discriminated by plotting Hoechst-W versus Hoechst-A. Identification of G2/M phase was performed by visualizing DNA content (Hoechst33258) and Anti-phospho-Histone H3 (Ser10).

(B) Gating strategy for the flow cytometry analysis of cell cycle progression after nocodazole block. Living cells were gated on a forward scatter (FSC)/side scatter (SSC) plot. Further, doublets were discriminated by plotting Hoechst-W versus Hoechst-A. Identification of G1 phase was performed by visualizing DNA content (Hoechst33258).

Supplemental Figure S15. Gating strategy for the flow cytometry analysis of MCM2 occupancy on DNA through cell cycle. Living cells were gated on a forward scatter (FSC)/side scatter (SSC) plot. Further, doublets were discriminated by plotting Hoechst-W versus Hoechst-A. Identification of individual phases of cell cycle was performed by visualizing DNA content (Hoechst-A versus Hoechst-W).

Supplemental Figure S16. Validation of MKi-67 expression levels. MKi-67 RPKM were taken from the GTEx Portal. RNA isolated from mouse organs was converted to cDNA and MKi-67 was quantified using qPCR. The MKi-67 RPKM values were compared to the expression values from our mice. These data show that the mice in our facility have similar MKi-67 expression levels as mice used for the GTEx portal.

Supplemental Table Legends

Supplemental Table S1. Proteins that interact with unmodified and 5hmC-modified DNA under our assay conditions. Proteins that interact with unmodified and 5hmC modified DNA substrates as identified by Electrospray and MALDI Mass spectrometry.

Supplemental Table S2. Results of Panther Gene Ontology Over-representation Test for proteins that interact exclusively with unmodified DNA. Proteins that exclusively bind unmodified DNA were ranked according to their gene ontology biological process.

Supplemental Table S3. Results of Panther Gene Ontology Over-representation Test for proteins that interact with both unmodified and 5hmC-modified DNA. Proteins that bind both unmodified and 5hmC-DNA were ranked according to their gene ontology biological process.

Supplemental Table S4. Results of Panther Gene Ontology Over-representation Test for proteins that interact exclusively with 5hmC-modified DNA. Proteins that exclusively bind 5hmC-DNA were ranked according to their gene ontology biological process.

Supplemental Table S5. Cell cycle analysis table of HeLa cell lines stably expressing YFP (Control), Tet2 CD fused to YFP (Tet2 CD), and Tet2 CD inactive mutant (Tet2

CD/CI). BrdU treated cells were stained with antibody against BrdU and PI for DNA content. First panel of the bable describes the percentage mean value of cells in each cell cycle phase ± standard deviation. Hours that cells spend in each cell cycle phase are shown in second panel of the table.

Supplemental Table S6. DNA construct sequences. Sequences of Tet2 CD:YFP and Tet2 CD/CI:YFP fusion protein constructs. These constructs were use to make the relevant stable HeLa cell lines.

Supplemental Table S7. Substrate sequences. Sequence of unmodified and 5hmC-modified substrates used in this study.

Appendix I – Figure 2 Gel Images

MCM2

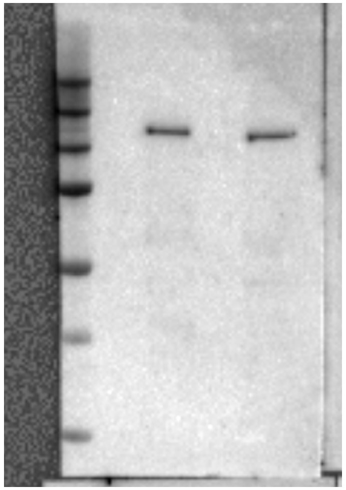

MCM3

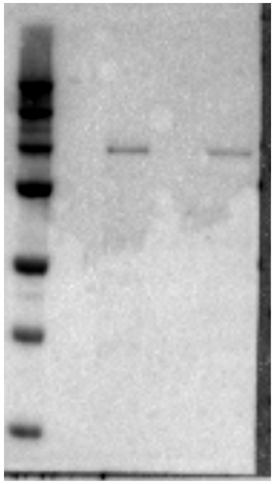

MCM4

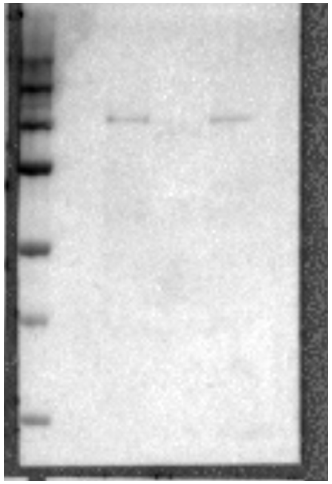

MSH2

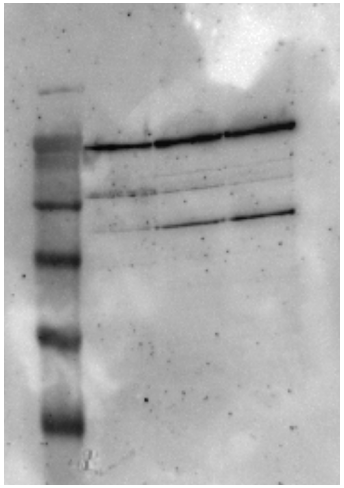

MCM5

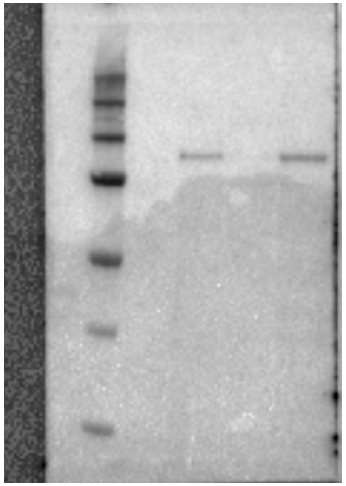

MCM6

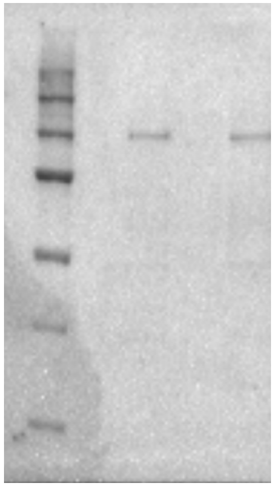

MCM7

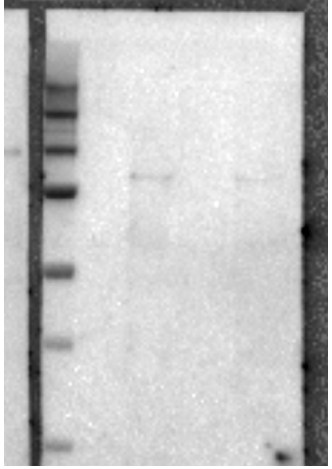

Appendix II – Figure 3 Gel Images

p15
INK4b ab 53034

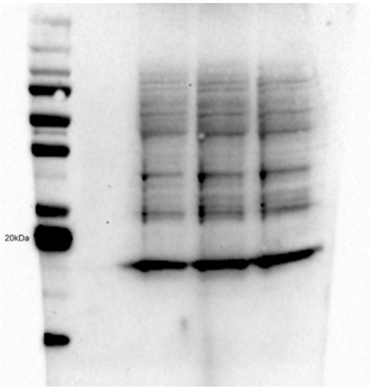

P16
INK4a ab108349

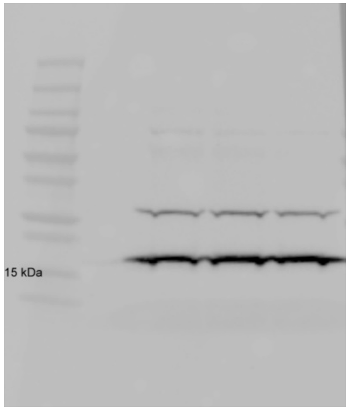

P18
INK4c EPR1591

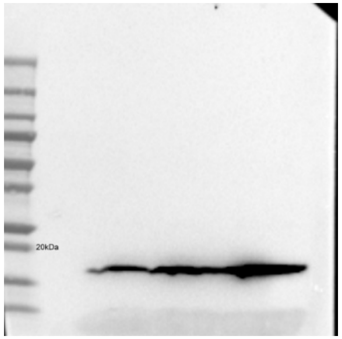

P19
INKd ab102842

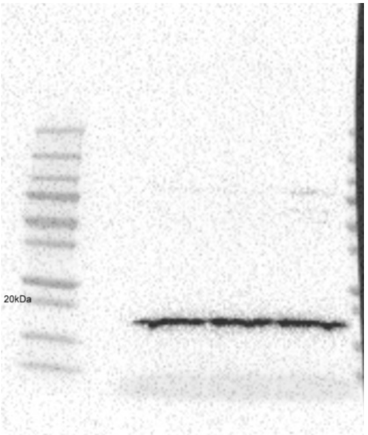

P21
ab188224

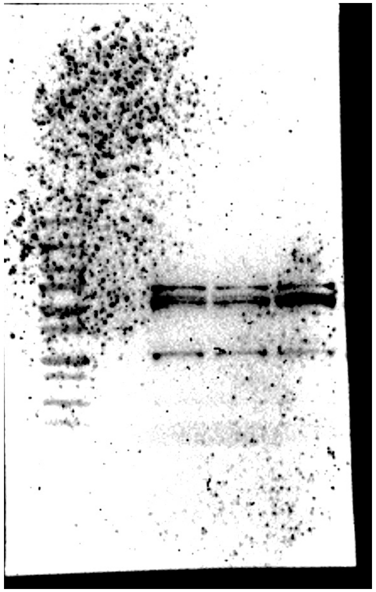

P 27

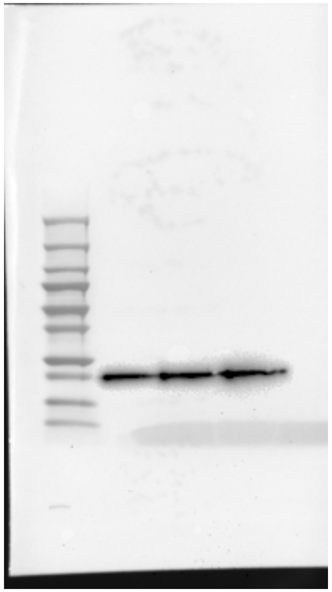

P53
#9282S

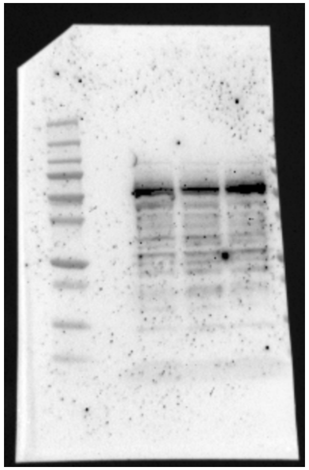

GAPDH

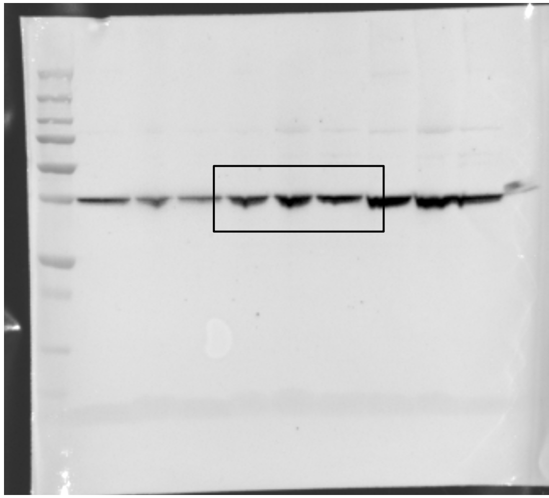

Duplicates Filtered from each List

Appears in Both Lists = RED

Unmodified Binding Proteins	5hmC Binding Proteins	Unmod Only	Unmodified and 5hmC Modified	5hmC Only
SFPQ_HUMAN	SFPQ_HUMAN	ELOA1_HUMAN	SFPQ_HUMAN	TOX4_HUMAN
K2C1_HUMAN	KU86_HUMAN	HNRL2_HUMAN	K2C1_HUMAN	KIF2C_HUMAN
TOP1_HUMAN	IF16_HUMAN	ATF7_HUMAN	TOP1_HUMAN	CUL4A_HUMAN
DYN2_HUMAN	TOP1_HUMAN	NFIC_HUMAN	DYN2_HUMAN	KIF2A_HUMAN
PARP1_HUMAN	PARP1_HUMAN	NR2C2_HUMAN	PARP1_HUMAN	SYQ_HUMAN
IF16_HUMAN	DYN2_HUMAN	ARFG2_HUMAN	IF16_HUMAN	HS90A_HUMAN
K1C10_HUMAN	K2C1_HUMAN	MUTYH_HUMAN	K1C10_HUMAN	FUBP2_HUMAN
HNRPU_HUMAN	UHRF1_HUMAN	NRF1_HUMAN	HNRPU_HUMAN	PF21A_HUMAN
K22E_HUMAN	K22E_HUMAN	TYY1_HUMAN	K22E_HUMAN	FOXK1_HUMAN
UHRF1_HUMAN	K1C10_HUMAN	HMBX1_HUMAN	UHRF1_HUMAN	TSR1_HUMAN
LSD1_HUMAN	TOX4_HUMAN	PLRG1_HUMAN	LSD1_HUMAN	AOF1_HUMAN
K2C5_HUMAN	CDC5L_HUMAN	INT12_HUMAN	K2C5_HUMAN	KU70_HUMAN
SYMC_HUMAN	UBF1_HUMAN	IMA2_HUMAN	SYMC_HUMAN	TERA_HUMAN
UBF1_HUMAN	HNRPU_HUMAN	TE2IP_HUMAN	UBF1_HUMAN	ERCC3_HUMAN
DNLI3_HUMAN	LSD1_HUMAN	AP2A_HUMAN	DNLI3_HUMAN	IMB1_HUMAN
KU86_HUMAN	SYMC_HUMAN	TAD3L_HUMAN	KU86_HUMAN	PO2F1_HUMAN
K1C9_HUMAN	KIF2C_HUMAN	SFRS6_HUMAN	K1C9_HUMAN	GTF2I_HUMAN
ELOA1_HUMAN	CUL4A_HUMAN	RING1_HUMAN	EMAL3_HUMAN	RAD21_HUMAN
EMAL3_HUMAN	DNLI3_HUMAN	VIME_HUMAN	EF1A1_HUMAN	GCNL2_HUMAN
EF1A1_HUMAN	KIF2A_HUMAN	DDX47_HUMAN	PRKDC_HUMAN	XRCC1_HUMAN
PRKDC_HUMAN	SYQ_HUMAN	NSUN5_HUMAN	CDC5L_HUMAN	MCM3_HUMAN
CDC5L_HUMAN	K1C9_HUMAN	RPC4_HUMAN	K1C14_HUMAN	IFNA1_HUMAN
HNRL2_HUMAN	HS90A_HUMAN	DDX49_HUMAN	NEUA_HUMAN	LMBL2_HUMAN
K1C14_HUMAN	EMAL3_HUMAN		ALBU_HUMAN	KIFC1_HUMAN
NEUA_HUMAN	FUBP2_HUMAN		MSH2_HUMAN	MEMO1_HUMAN
ALBU_HUMAN	EF1A1_HUMAN		NONO_HUMAN	WIZ_HUMAN
MSH2_HUMAN	PF21A_HUMAN		PNKP_HUMAN	MRE11_HUMAN
NONO_HUMAN	FOXK1_HUMAN		TERF2_HUMAN	HNRL1_HUMAN
PNKP_HUMAN	TSR1_HUMAN		IF2G_HUMAN	PP1RA_HUMAN
TERF2_HUMAN	AOF1_HUMAN		TFCP2_HUMAN	K2C6A_HUMAN
ATF7_HUMAN	PRKDC_HUMAN		PRP19_HUMAN	NPM3_HUMAN
IF2G_HUMAN	KU70_HUMAN		RCOR1_HUMAN	PIP_HUMAN
TFCP2_HUMAN	TERA_HUMAN		MEF2D_HUMAN	FILA2_HUMAN
PRP19_HUMAN	ERCC3_HUMAN		PTBP1_HUMAN	TCPD_HUMAN
RCOR1_HUMAN	IMB1_HUMAN		NFIA_HUMAN	IGHA1_HUMAN
MEF2D_HUMAN	PO2F1_HUMAN		HDAC1_HUMAN	RCC2_HUMAN
PTBP1_HUMAN	GTF2I_HUMAN		UBIP1_HUMAN	PHKG1_HUMAN
NFIA_HUMAN	RAD21_HUMAN		NFIB_HUMAN	HBA_HUMAN
HDAC1_HUMAN	GCNL2_HUMAN		SYDC_HUMAN	K1C16_HUMAN
UBIP1_HUMAN	XRCC1_HUMAN		DMAP1_HUMAN	HNRPD_HUMAN
NFIB_HUMAN	MSH2_HUMAN		KLF5_HUMAN	DDB2_HUMAN
SYDC_HUMAN	MCM3_HUMAN		HNRPL_HUMAN	ERR1_HUMAN
DMAP1_HUMAN	IFNA1_HUMAN		KHDR1_HUMAN	IF4A3_HUMAN
NFIC_HUMAN	LMBL2_HUMAN		TF3C5_HUMAN	RCC1_HUMAN
KLF5_HUMAN	KIFC1_HUMAN		RUVB2_HUMAN	LDB1_HUMAN
HNRPL_HUMAN	MEMO1_HUMAN		SUH_HUMAN	TBG1_HUMAN
KHDR1_HUMAN	WIZ_HUMAN		RBBP4_HUMAN	K1C18_HUMAN
TF3C5_HUMAN	MRE11_HUMAN		U2AF2_HUMAN	PAX6_HUMAN
RUVB2_HUMAN	HNRL1_HUMAN		NUSAP_HUMAN	
SUH_HUMAN	PP1RA_HUMAN		ST65G_HUMAN	
RBBP4_HUMAN	NONO_HUMAN		DKC1_HUMAN	
NR2C2_HUMAN	SYDC_HUMAN		NFIL3_HUMAN	
U2AF2_HUMAN	HNRPK_HUMAN		RFA1_HUMAN	
NUSAP_HUMAN	IF2G_HUMAN		EXOS9_HUMAN	
ST65G_HUMAN	PNKP_HUMAN		HDAC2_HUMAN	
ARFG2_HUMAN	TERF2_HUMAN		K2C8_HUMAN	
DKC1_HUMAN	PRP19_HUMAN		SYFA_HUMAN	
NFIL3_HUMAN	K2C6A_HUMAN		K2C6B_HUMAN	
RFA1_HUMAN	K2C8_HUMAN		HNRPK_HUMAN	
MUTYH_HUMAN	PTBP1_HUMAN		CAF1B_HUMAN	
NRF1_HUMAN	K1C14_HUMAN		RUVB1_HUMAN	
TYY1_HUMAN	UBIP1_HUMAN		HNRPR_HUMAN	
EXOS9_HUMAN	HDAC2_HUMAN		CPSF7_HUMAN	
HDAC2_HUMAN	RCOR1_HUMAN		DEK_HUMAN	
K2C8_HUMAN	MEF2D_HUMAN		DERPC_HUMAN	
SYFA_HUMAN	K2C5_HUMAN		NOL4_HUMAN	
K2C6B_HUMAN	TFCP2_HUMAN		TBL1X_HUMAN	
HNRPK_HUMAN	NFIB_HUMAN		TBA1A_HUMAN	
CAF1B_HUMAN	SUH_HUMAN		TBL1R_HUMAN	
RUVB1_HUMAN	U2AF2_HUMAN		SF3A3_HUMAN	
HMBX1_HUMAN	CAF1B_HUMAN		HNRH1_HUMAN	
HNRPR_HUMAN	RBBP4_HUMAN		TBB5_HUMAN	
CPSF7_HUMAN	RUVB2_HUMAN		TBB2C_HUMAN	
DEK_HUMAN	NFIA_HUMAN		RBBP7_HUMAN	
PLRG1_HUMAN	TBL1X_HUMAN		PAIRB_HUMAN	
DERPC_HUMAN	ALBU_HUMAN		PSIP1_HUMAN	
INT12_HUMAN	NUSAP_HUMAN		DDX39_HUMAN	
NOL4_HUMAN	SYFA_HUMAN			
TBL1X_HUMAN	KHDR1_HUMAN			
TBA1A_HUMAN	HNRPL_HUMAN			
TBL1R_HUMAN	RFA1_HUMAN			
SF3A3_HUMAN	NOL4_HUMAN			
IMA2_HUMAN	DMAP1_HUMAN			
HNRH1_HUMAN	SF3A3_HUMAN			
TBB5_HUMAN	HDAC1_HUMAN			
TBB2C_HUMAN	NPM3_HUMAN			
RBBP7_HUMAN	PIP_HUMAN			
TE2IP_HUMAN	HNRPR_HUMAN			
AP2A_HUMAN	FILA2_HUMAN			
PAIRB_HUMAN	EXOS9_HUMAN			
TAD3L_HUMAN	KLF5_HUMAN			
SFRS6_HUMAN	ST65G_HUMAN			
RING1_HUMAN	RUVB1_HUMAN			
VIME_HUMAN	TCPD_HUMAN			
DDX47_HUMAN	NFIL3_HUMAN			
NSUN5_HUMAN	IGHA1_HUMAN			
RPC4_HUMAN	TBL1R_HUMAN			
PSIP1_HUMAN	RCC2_HUMAN			
DDX49_HUMAN	PHKG1_HUMAN			
DDX39_HUMAN	TF3C5_HUMAN			
	DKC1_HUMAN			
	K2C6B_HUMAN			
	CPSF7_HUMAN			
	HNRH1_HUMAN			
	DEK_HUMAN			
	DERPC_HUMAN			
	HBA_HUMAN			
	TBB5_HUMAN			
	NEUA_HUMAN			
	K1C16_HUMAN			
	RBBP7_HUMAN			
	TBA1A_HUMAN			
	HNRPD_HUMAN			
	TBB2C_HUMAN			
	DDB2_HUMAN			
	PAIRB_HUMAN			
	ERR1_HUMAN			
	PSIP1_HUMAN			
	DDX39_HUMAN			
	IF4A3_HUMAN			
	RCC1_HUMAN			
	LDB1_HUMAN			
	TBG1_HUMAN			
	K1C18_HUMAN			
	PAX6_HUMAN			

Unmodified DNA Binding Proteins

Analysis Type:	PANTHER Overrepresentation Test (release 20170413)
Annotation Version and Release Date:	GO Ontology database Released 2017-05-25
Analyzed List:	Unmodified (Homo sapiens)
Reference List:	Homo sapiens (all genes in database)
Bonferroni correction:	TRUE
Bonferroni count:	8492

GO biological process complete	Homo sapiens - REFLIST (20972)	Unmodified (20)	Unmodified (expected)	Unmodified (over/under)	Unmodified (fold Enrichment)	Jnmodified (P-value)
RNA metabolic process (GO:0016070)	3434	16	3.27	+	4.89	5.63E-06
transcription, DNA-templated (GO:0006351)	2586	12	2.47	+	4.87	5.04E-03
nucleic acid-templated transcription (GO:0097659)	2587	12	2.47	+	4.86	5.06E-03
RNA biosynthetic process (GO:0032774)	2597	12	2.48	+	4.85	5.28E-03
gene expression (GO:0010467)	3722	16	3.55	+	4.51	1.92E-05
nucleic acid metabolic process (GO:0090304)	3956	17	3.77	+	4.51	2.61E-06
nucleobase-containing compound biosynthetic process (GO:0034654)	2944	12	2.81	+	4.27	2.07E-02
heterocycle biosynthetic process (GO:0018130)	3014	12	2.87	+	4.17	2.67E-02
aromatic compound biosynthetic process (GO:0019438)	3017	12	2.88	+	4.17	2.70E-02
organic cyclic compound biosynthetic process (GO:1901362)	3152	12	3.01	+	3.99	4.32E-02
nucleobase-containing compound metabolic process (GO:0006139)	4512	17	4.30	+	3.95	2.22E-05
heterocycle metabolic process (GO:0046483)	4685	17	4.47	+	3.80	4.09E-05
cellular aromatic compound metabolic process (GO:0006725)	4722	17	4.50	+	3.78	4.64E-05
organic cyclic compound metabolic process (GO:1901360)	4945	17	4.72	+	3.60	9.79E-05
cellular nitrogen compound metabolic process (GO:0034641)	5120	17	4.88	+	3.48	1.72E-04
cellular macromolecule metabolic process (GO:0044260)	6946	17	6.62	+	2.57	2.19E-02

Unmod and 5hmC

Analysis Type:	PANTHER Overrepresentation Test (release 20170413)
Annotation Version and Release Date:	GO Ontology database Released 2017-05-25
Analyzed List:	Unmod and 5hmC (Homo sapiens)
Reference List:	Homo sapiens (all genes in database)
Bonferroni correction:	TRUE
Bonferroni count:	8492

GO biological process complete	Homo sapiens - REFLIST (20972)	Unmod and 5hmC (71)	Unmod and 5hmC (expected)	Unmod and 5hmC (over/under)	Unmod and 5hmC (fold Enrichment)	Unmod and 5hmC (P-value)
histone H4 deacetylation (GO:0070933)	9	3	.03	+	98.46	3.75E-02
histone deacetylation (GO:0016575)	58	8	.20	+	40.74	2.65E-07
protein deacetylation (GO:0006476)	60	8	.20	+	39.38	3.45E-07
protein deacylation (GO:0035601)	65	8	.22	+	36.35	6.47E-07
macromolecule deacylation (GO:0098732)	66	8	.22	+	35.80	7.29E-07
regulation of telomere maintenance via telomere lengthening (GO:1904356)	61	5	.21	+	24.21	1.96E-02
ATP-dependent chromatin remodeling (GO:0043044)	63	5	.21	+	23.44	2.29E-02
cornification (GO:0070268)	110	8	.37	+	21.48	3.86E-05
skin epidermis development (GO:0098773)	83	6	.28	+	21.35	3.75E-03
regulation of mRNA splicing, via spliceosome (GO:0048024)	72	5	.24	+	20.51	4.37E-02
chromatin remodeling (GO:0006338)	149	9	.50	+	17.84	1.96E-05
regulation of RNA splicing (GO:0043484)	110	6	.37	+	16.11	1.89E-02
circadian rhythm (GO:0007623)	135	7	.46	+	15.32	3.61E-03
mRNA splicing, via spliceosome (GO:0000398)	284	13	.96	+	13.52	1.22E-07
RNA splicing, via transesterification reactions with bulged adenosine as nucleophile (GO:0000377)	284	13	.96	+	13.52	1.22E-07
RNA splicing, via transesterification reactions (GO:0000375)	287	13	.97	+	13.38	1.39E-07
DNA recombination (GO:0006310)	226	10	.77	+	13.07	4.55E-05
double-strand break repair (GO:0006302)	163	7	.55	+	12.69	1.25E-02
DNA replication (GO:0006260)	213	9	.72	+	12.48	4.12E-04
histone modification (GO:0016570)	346	13	1.17	+	11.10	1.35E-06
protein-DNA complex assembly (GO:0065004)	192	7	.65	+	10.77	3.65E-02
epidermal cell differentiation (GO:0009913)	308	11	1.04	+	10.55	6.63E-05
keratinization (GO:0031424)	224	8	.76	+	10.55	8.41E-03
keratinocyte differentiation (GO:0030216)	266	9	.90	+	9.99	2.64E-03
RNA splicing (GO:0008380)	393	13	1.33	+	9.77	6.28E-06
DNA conformation change (GO:0071103)	254	8	.86	+	9.30	2.12E-02
rhythmic process (GO:0048511)	288	9	.98	+	9.23	5.10E-03
DNA metabolic process (GO:0006259)	777	24	2.63	+	9.12	3.68E-13
mRNA processing (GO:0006397)	454	14	1.54	+	9.11	3.18E-06
DNA repair (GO:0006281)	489	15	1.66	+	9.06	7.40E-07
covalent chromatin modification (GO:0016569)	465	14	1.57	+	8.89	4.32E-06
skin development (GO:0043588)	372	11	1.26	+	8.73	4.46E-04
epidermis development (GO:0008544)	405	11	1.37	+	8.02	1.04E-03
chromatin organization (GO:0006325)	648	16	2.19	+	7.29	3.72E-06
mRNA metabolic process (GO:0016071)	663	16	2.24	+	7.13	5.17E-06
cellular response to DNA damage stimulus (GO:0006974)	731	17	2.47	+	6.87	2.40E-06
chromosome organization (GO:0051276)	1009	23	3.42	+	6.73	1.15E-09
negative regulation of transcription from RNA polymerase II promoter (GO:0000122)	772	16	2.61	+	6.12	4.48E-05
epithelial cell differentiation (GO:0030855)	653	13	2.21	+	5.88	2.35E-03
negative regulation of nucleobase-containing compound metabolic process (GO:0045934)	1357	25	4.59	+	5.44	8.33E-09
RNA processing (GO:0006396)	870	16	2.95	+	5.43	2.36E-04
negative regulation of cellular macromolecule biosynthetic process (GO:2000113)	1318	23	4.46	+	5.15	2.64E-07
negative regulation of RNA metabolic process (GO:0051253)	1205	21	4.08	+	5.15	2.47E-06
negative regulation of transcription, DNA-templated (GO:0045892)	1117	19	3.78	+	5.02	3.17E-05
positive regulation of transcription from RNA polymerase II promoter (GO:0045944)	1064	18	3.60	+	5.00	9.72E-05
positive regulation of RNA metabolic process (GO:0051254)	1429	24	4.84	+	4.96	1.90E-07
negative regulation of macromolecule biosynthetic process (GO:0010558)	1389	23	4.70	+	4.89	7.48E-07
negative regulation of nucleic acid-templated transcription (GO:1903507)	1160	19	3.93	+	4.84	5.84E-05
negative regulation of RNA biosynthetic process (GO:1902679)	1162	19	3.93	+	4.83	6.00E-05
positive regulation of nucleobase-containing compound metabolic process (GO:0045935)	1684	27	5.70	+	4.74	1.94E-08
positive regulation of gene expression (GO:0010628)	1720	27	5.82	+	4.64	3.18E-08
negative regulation of cellular biosynthetic process (GO:0031327)	1466	23	4.96	+	4.63	2.16E-06
negative regulation of gene expression (GO:0010629)	1597	25	5.41	+	4.62	2.85E-07
negative regulation of biosynthetic process (GO:0009890)	1487	23	5.03	+	4.57	2.86E-06
positive regulation of nucleic acid-templated transcription (GO:1903508)	1378	21	4.67	+	4.50	2.73E-05
positive regulation of transcription, DNA-templated (GO:0045893)	1378	21	4.67	+	4.50	2.73E-05
positive regulation of RNA biosynthetic process (GO:1902680)	1379	21	4.67	+	4.50	2.76E-05
positive regulation of macromolecule biosynthetic process (GO:0010557)	1628	24	5.51	+	4.35	2.74E-06
cellular response to stress (GO:0033554)	1561	23	5.28	+	4.35	7.34E-06
regulation of transcription from RNA polymerase II promoter (GO:0006357)	1908	27	6.46	+	4.18	3.46E-07
transcription, DNA-templated (GO:0006351)	2586	36	8.75	+	4.11	4.08E-11
nucleic acid-templated transcription (GO:0097659)	2587	36	8.76	+	4.11	4.13E-11
RNA biosynthetic process (GO:0032774)	2597	36	8.79	+	4.09	4.66E-11
positive regulation of cellular biosynthetic process (GO:0031328)	1761	24	5.96	+	4.03	1.33E-05
nucleobase-containing compound biosynthetic process (GO:0034654)	2944	40	9.97	+	4.01	8.79E-13
positive regulation of biosynthetic process (GO:0009891)	1791	24	6.06	+	3.96	1.86E-05
nucleic acid metabolic process (GO:0090304)	3956	53	13.39	+	3.96	2.69E-20
RNA metabolic process (GO:0016070)	3434	46	11.63	+	3.96	7.67E-16
heterocycle biosynthetic process (GO:0018130)	3014	40	10.20	+	3.92	2.00E-12
aromatic compound biosynthetic process (GO:0019438)	3017	40	10.21	+	3.92	2.07E-12
organic cyclic compound biosynthetic process (GO:1901362)	3152	40	10.67	+	3.75	9.53E-12
gene expression (GO:0010467)	3722	47	12.60	+	3.73	2.37E-15
cellular nitrogen compound biosynthetic process (GO:0044271)	3455	43	11.70	+	3.68	6.09E-13
cellular macromolecule biosynthetic process (GO:0034645)	3659	45	12.39	+	3.63	8.76E-14
macromolecule biosynthetic process (GO:0009059)	3723	45	12.60	+	3.57	1.74E-13
negative regulation of nitrogen compound metabolic process (GO:0051172)	2249	27	7.61	+	3.55	1.38E-05
negative regulation of macromolecule metabolic process (GO:0010605)	2444	29	8.27	+	3.50	3.31E-06
nucleobase-containing compound metabolic process (GO:0006139)	4512	53	15.28	+	3.47	1.60E-17
regulation of nucleobase-containing compound metabolic process (GO:0019219)	4141	47	14.02	+	3.35	2.01E-13
heterocycle metabolic process (GO:0046483)	4685	53	15.86	+	3.34	9.75E-17
negative regulation of cellular metabolic process (GO:0031324)	2405	27	8.14	+	3.32	5.93E-05
cellular aromatic compound metabolic process (GO:0006725)	4722	53	15.99	+	3.32	1.42E-16
regulation of RNA metabolic process (GO:0051252)	3777	41	12.79	+	3.21	7.46E-10
negative regulation of metabolic process (GO:0009892)	2688	29	9.10	+	3.19	3.07E-05
positive regulation of macromolecule metabolic process (GO:0010604)	2892	31	9.79	+	3.17	7.52E-06
organic cyclic compound metabolic process (GO:1901360)	4945	53	16.74	+	3.17	1.29E-15
organelle organization (GO:0006996)	3097	33	10.48	+	3.15	1.75E-06
cellular nitrogen compound metabolic process (GO:0034641)	5120	54	17.33	+	3.12	7.16E-16
regulation of cellular macromolecule biosynthetic process (GO:2000112)	4063	42	13.76	+	3.05	1.55E-09
regulation of gene expression (GO:0010468)	4544	46	15.38	+	2.99	6.18E-11
regulation of macromolecule biosynthetic process (GO:0010556)	4169	42	14.11	+	2.98	3.84E-09
positive regulation of nitrogen compound metabolic process (GO:0051173)	2814	28	9.53	+	2.94	3.88E-04
regulation of transcription, DNA-templated (GO:0006355)	3627	36	12.28	+	2.93	1.11E-06
positive regulation of metabolic process (GO:0009893)	3129	31	10.59	+	2.93	5.22E-05
regulation of nucleic acid-templated transcription (GO:1903506)	3651	36	12.36	+	2.91	1.35E-06
regulation of RNA biosynthetic process (GO:2001141)	3659	36	12.39	+	2.91	1.44E-06
cellular component assembly (GO:0022607)	2254	22	7.63	+	2.88	2.62E-02
organic substance biosynthetic process (GO:1901576)	4742	46	16.05	+	2.87	3.28E-10
cellular biosynthetic process (GO:0044249)	4655	45	15.76	+	2.86	9.98E-10
regulation of cellular biosynthetic process (GO:0031326)	4362	42	14.77	+	2.84	1.86E-08
positive regulation of cellular metabolic process (GO:0031325)	2917	28	9.88	+	2.84	8.42E-04
cellular component biogenesis (GO:0044085)	2508	24	8.49	+	2.83	1.10E-02
biosynthetic process (GO:0009058)	4809	46	16.28	+	2.83	5.66E-10
regulation of biosynthetic process (GO:0009889)	4432	42	15.00	+	2.80	3.22E-08
response to stress (GO:0006950)	3263	29	11.05	+	2.63	2.37E-03
cellular component organization (GO:0016043)	5242	45	17.75	+	2.54	8.33E-08
cellular macromolecule metabolic process (GO:0044260)	6946	59	23.52	+	2.51	4.68E-14
cellular component organization or biogenesis (GO:0071840)	5457	46	18.47	+	2.49	7.05E-08
regulation of macromolecule metabolic process (GO:0060255)	6010	49	20.35	+	2.41	1.93E-08
regulation of nitrogen compound metabolic process (GO:0051171)	5811	47	19.67	+	2.39	1.46E-07
regulation of primary metabolic process (GO:0080090)	5976	47	20.23	+	2.32	4.23E-07
regulation of cellular metabolic process (GO:0031323)	6029	47	20.41	+	2.30	5.90E-07
macromolecule metabolic process (GO:0043170)	7685	59	26.02	+	2.27	9.70E-12
regulation of metabolic process (GO:0019222)	6533	49	22.12	+	2.22	5.40E-07
negative regulation of cellular process (GO:0048523)	4291	32	14.53	+	2.20	2.26E-02
negative regulation of biological process (GO:0048519)	4778	34	16.18	+	2.10	2.58E-02
nitrogen compound metabolic process (GO:0006807)	8534	58	28.89	+	2.01	1.46E-08
cellular metabolic process (GO:0044237)	8936	60	30.25	+	1.98	3.28E-09
primary metabolic process (GO:0044238)	9069	58	30.70	+	1.89	2.86E-07
organic substance metabolic process (GO:0071704)	9416	60	31.88	+	1.88	4.91E-08
metabolic process (GO:0008152)	9878	60	33.44	+	1.79	5.64E-07
cellular process (GO:0009987)	14882	68	50.38	+	1.35	9.80E-04
Unclassified (UNCLASSIFIED)	3700	2	12.53	-	< 0.2	0.00E00

5hmC Binding Proteins

Analysis Type:	PANTHER Overrepresentation Test (release 20160715)
Annotation Version and Release Date:	GO Ontology database Released 2017-02-28
Analyzed List:	5hmC (Homo sapiens)
Reference List:	Homo sapiens (all genes in database)
Bonferroni correction:	TRUE
Bonferroni count:	8204

GO biological process complete	Homo sapiens - REFLIST (20972)	5hmC (46)	5hmC (expected)	5hmC (over/under)	5hmC (fold Enrichment)	5hmC (P-value)
DNA duplex unwinding (GO:0032508)	77	5	.17	+	29.60	6.62E-03
DNA geometric change (GO:0032392)	87	5	.19	+	26.20	1.20E-02
sister chromatid cohesion (GO:0007062)	114	5	.25	+	20.00	4.43E-02
sister chromatid segregation (GO:0000819)	183	7	.40	+	17.44	1.26E-03
nuclear chromosome segregation (GO:0098813)	254	7	.56	+	12.56	1.11E-02
chromosome segregation (GO:0007059)	295	8	.65	+	12.36	2.04E-03
chromosome organization (GO:0051276)	1012	16	2.22	+	7.21	1.75E-06
mitotic cell cycle process (GO:1903047)	766	12	1.68	+	7.14	5.63E-04
mitotic cell cycle (GO:0000278)	794	12	1.74	+	6.89	8.30E-04
DNA metabolic process (GO:0006259)	781	10	1.71	+	5.84	5.00E-02
cell cycle process (GO:0022402)	1091	12	2.39	+	5.01	2.37E-02
cell cycle (GO:0007049)	1337	13	2.93	+	4.43	3.24E-02
organelle organization (GO:0006996)	3186	24	6.99	+	3.43	4.66E-05
nucleic acid metabolic process (GO:0090304)	3978	24	8.73	+	2.75	3.73E-03
nucleobase-containing compound metabolic process (GO:0006139)	4531	24	9.94	+	2.41	4.29E-02
cellular component organization or biogenesis (GO:0071840)	5493	29	12.05	+	2.41	1.37E-03
cellular component organization (GO:0016043)	5278	27	11.58	+	2.33	1.18E-02
Unclassified (UNCLASSIFIED)	3732	3	8.19	-	.37	0.00E00

	G1 (%)	S (%)	G2/M (%)	Polyploid (%)	G1 (hrs)	S (hrs)	G2/M (hrs)	Polyploid (hrs)
Control	28.67 ± 2.37	25.24 ± 1.49	27.04 ± 0.91	15.46 ± 2.83	4.59 ± 0.38	4.1 ± 0.24	4.49 ± 0.15	2.51 ± 0.45
Tet2 CD	47.25 ± 2.56	35.48 ± 1.91	12.90 ± 1.77	1.44 ± 0.84	7.57 ± 0.49	5.68 ± 0.37	2.06 ± 0.34	0.23 ± 0.16
Tet2 CD/CI	45.78 ± 2.37	38.17 ± 1.76	11.14 ±0.91	0.61 ± 2.83	7.32 ± 0.24	6.11 ± 0.91	1.78 ± 0.10	0.10 ± 0.07
	Two Tailed Students' t-tests				Two Tailed Students' t-tests			
Control vs Tet2 CD	< 0.001	0.002	< 0.001	0.001	< 0.001	< 0.001	< 0.001	0.001
Control vs Tet2 CD/CI	< 0.001	0.017	0.243	0.001	< 0.001	< 0.001	0.291	< 0.001
Tet2 CD vs Tet2 CD/CI	0.290	0.596	0.416	0.219	0.097	0.876	0.267	0.207

Supplemental Table S6

Tet2 cDNA constructs used to create stable cell lines

```
>pEYFP-Tet2 CD
     TAGTTATTAA TAGTAATCAA TTACGGGGTC ATTAGTTCAT AGCCCATATA TGGAGTTCCG        60
     CGTTACATAA CTTACGGTAA ATGGCCCGCC TGGCTGACCG CCCAACGACC CCCGCCCATT       120
     GACGTCAATA ATGACGTATG TTCCCATAGT AACGCCAATA GGGACTTTCC ATTGACGTCA       180
     ATGGGTGGAG TATTTACGGT AAACTGCCCA CTTGGCAGTA CATCAAGTGT ATCATATGCC       240
     AAGTACGCCC CCTATTGACG TCAATGACGG TAAATGGCCC GCCTGGCATT ATGCCCAGTA       300
     CATGACCTTA TGGGACTTTC CTACTTGGCA GTACATCTAC GTATTAGTCA TCGCTATTAC       360
     CATGGTGATG CGGTTTTGGC AGTACATCAA TGGGCGTGGA TAGCGGTTTG ACTCACGGGG       420
     ATTTCCAAGT CTCCACCCCA TTGACGTCAA TGGGAGTTTG TTTTGGCACC AAAATCAACG       480
     GGACTTTCCA AAATGTCGTA ACAACTCCGC CCCATTGACG CAAATGGGCG GTAGGCGTGT       540
     ACGGTGGGAG GTCTATATAA GCAGAGCTGG TTTAGTGAAC CGTCAGATCC GCTAGCGCTA       600
     CCGGTCGCCA CCATGGTGAG CAAGGGCGAG GAGCTGTTCA CCGGGGTGGT GCCCATCCTG       660
     GTCGAGCTGG ACGGCGACGT AAACGGCCAC AAGTTCAGCG TGTCCGGCGA GGGCGAGGGC       720
     GATGCCACCT ACGGCAAGCT GACCCTGAAG TTCATCTGCA CCACCGGCAA GCTGCCCGTG       780
     CCCTGGCCCA CCCTCGTGAC CACCTTCGGC TACGGCCTGC AGTGCTTCGC CCGCTACCCC       840
     GACCACATGA AGCAGCACGA CTTCTTCAAG TCCGCCATGC CCGAAGGCTA CGTCCAGGAG       900
     CGCACCATCT TCTTCAAGGA CGACGGCAAC TACAAGACCC GCGCCGAGGT GAAGTTCGAG       960
     GGCGACACCC TGGTGAACCG CATCGAGCTG AAGGGCATCG ACTTCAAGGA GGACGGCAAC      1020
     ATCCTGGGGC ACAAGCTGGA GTACAACTAC AACAGCCACA ACGTCTATAT CATGGCCGAC      1080
     AAGCAGAAGA ACGGCATCAA GGTGAACTTC AAGATCCGCC ACAACATCGA GGACGGCAGC      1140
     GTGCAGCTCG CCGACCACTA CCAGCAGAAC ACCCCCATCG GCGACGGCCC CGTGCTGCTG      1200
     CCCGACAACC ACTACCTGAG CTACCAGTCC GCCCTGAGCA AAGACCCCAA CGAGAAGCGC      1260
     GATCACATGG TCCTGCTGGA GTTCGTGACC GCCGCCGGGA TCACTCTCGG CATGGACGAG      1320
     CTGTACAAGT CCGGACTCAG ATCTCGAGCT CAAGCTTCGA ATTCTGCAGT CGACGGTACC      1380
     GCGGGCCCGG CGCTTATGGA CTACAAAGAC GATGACGACA AGGGATCCAC TAGTAACGGC      1440
     CGCCAGTGTG CTGGAATTCG CCCTTTACAA AGTCAGAATG GCAAATGTGA AGGATGCAAT      1500
     CCAGACAAAG ATGAAGCTCC TTATTATACC CATCTGGGAG CTGGTCCTGA TGTGGCAGCT      1560
     ATTAGAACAC TCATGGAAGA AAGGTATGGA GAGAAGGGTA AAGCTATTAG GATTGAAAAA      1620
     GTCATATATA CTGGTAAAGA AGGCAAGAGC TCTCAGGGAT GTCCTATTGC TAAATGGGTA      1680
     TATCGGAGAT CGAGTGAGGA GGAGAAACTA CTGTGTTTGG TACGAGTGCG ACCTAATCAC      1740
     ACATGTGAGA CGGCGGTGAT GGTAATTGCC ATCATGTTGT GGGACGGAAT CCCAAAGCTA      1800
     CTCGCATCAG AACTCTACTC AGAACTTACA GATATCTTGG GCAAGTGTGG CATATGCACC      1860
     AACCGTCGCT GTTCTCAGAA TGAAACTAGA AACTGTTGTT GTCAGGGTGA GAATCCAGAG      1920
     ACCTGTGGTG CCTCCTTTTC TTTTGGTTGT TCTTGGAGCA TGTACTATAA TGGATGTAAG      1980
     TTTGCCAGAA GCAAGAAACC AAGGAAATTT AGGCTACATG GAGCTGAGCC AAAAGAGGAA      2040
     GAGAGACTAG GTTCTCATTT GCAAAACCTG GCTACTGTCA TTGCTCCAAT ATACAAGAAG      2100
     CTTGCACCCG ATGCATACAA TAATCAGGTT GAATTTGAAC ACCAAGCCCC AGACTGCTGT      2160
     TTGGGTCTGA AGGAAGGCCG GCCATTCTCA GGAGTCACTG CATGTTTGGA CTTCTCTGCT      2220
     CATTCCCACA GAGACCAGCA GAACATGCCA AATGGCAGTA CAGTGGTGGT CACCCTCAAT      2280
     AGAGAAGACA ATCGAGAAGT CGGAGCTAAG CCTGAGGATG AGCAGTTCCA CGTGCTGCCT      2340
     ATGTACATCA TCGCCCCTGA GGATGAGTTT GGGAGTACGG AAGGCCAGGA GAAGAAGATA      2400
     CGGATGGGGT CCATTGAGGT TCTGCAGTCA TTTCGGAGGA GAAGGGTCAT AAGGATAGGA      2460
     GAGCTGCCCA AGAGTTGCAA GAAGAAAGCG GAGCCCAAGA AAGCCAAGAC CAAGAAAGCA      2520
     GCTCGAAAGC GTTCCTCTCT GGAGAACTGC TCCAGTAGGA CTGAGAAGGG AAAGTCTTCC      2580
     TCACATACAA AGCTGATGGA AAATGCAAGC CATATGAAAC AAATGACAGC ACAACCGCAG      2640
     CTTTCGGGCC CGGTCATCCG GCAGCCACCA ACACTCCAGA GGCACCTTCA GCAAGGGCAG      2700
     AGGCCACAGC AGCCGCAGCC ACCTCAGCCG CAGCCGCAGA CGACACCTCA GCCACAGCCA      2760
     CAGCCACAGC ATATCATGCC CGGTAACTCT CAGTCTGTTG GTTCTCATTG TTCTGGATCC      2820
     ACCAGTGTCT ACACGAGACA GCCTACTCCT CACAGTCCTT ATCCCAGCTC AGCACACACC      2880
     TCAGATATTT ATGGAGATAC CAACCATGTG AACTTTTACC CCACTTCATC TCATGCCTCG      2940
     GGTTCATATT TGAATCCTTC TAATTACATG AACCCCTACC TTGGGCTTTT GAATCAGAAT      3000
     AACCAATATG CACCTTTTCC ATACAATGGG AGTGTGCCAG TGGACAATGG TTCCCCTTTC      3060
     TTAGGTTCTT ATTCCCCCCA GGCTCAGTCC AGGGATCTAC ATAGATATCC AAACCAGGAC      3120
     CATCTCACCA ATCAGAACTT ACCACCCATC CACACCCTTC ACCAACAGAC GTTTGGGGAC      3180
     AGTCCCTCTA AGTACTTAAG TTATGGAAAC CAAAATATGC AGAGAGATGC CTTCACTACT      3240
     AACTCCACCC TAAAACCAAA TGTACACCAC CTAGCAACGT TTTCTCCTTA CCCCACCCCC      3300
     AAGATGGATA GTCATTTCAT GGGAGCTGCC TACAGATCAC CATACAGCCA CCCACACACT      3360
     GACTACAAAA CCAGTGAGCA TCATCTACCC TCTCACACGG TCTACAGCTA CACGGCAGCA      3420
     GCTTCGGGGA GCAGTTCCAG CCACGCCTTC CACAACAAGG AGAATGACAA CATAGCCAAT      3480
     GGGCTCTCAA GAGTGCTTCC AGGGTTTAAT CATGATAGAA CTGCTTCTGC CCAAGAACTA      3540
     TTATACAGTC TGACTGGCAG CAGTCAGGAG AAGCAGCCTG AGGTGTCAGG CCAGGATGCA      3600
     GCTGCTGTGC AGGAAATTGA GTATTGGTCA GATAGTGAGC ACAACTTTCA GGATCCTTGC      3660
     ATTGGAGGGG TGGCTATAGC CCCAACTCAT GGGTCAATTC TTATTGAGTG TGCAAAGTGT      3720
     GAGGTTCATG CCACAACCAA AGTAAACGAT CCCGACCGGA ATCACCCCAC CAGGATCTCA      3780
     CTTGTACTGT ATAGGCATAA GAATTTGTTT CTACCAAAAC ATTGTTTGGC TCTCTGGGAA      3840
     GCCAAAATGG CTGAAAAGGC CCGGAAAGAG GAAGAGTGCG GAAAGAATGG ATCAGACCAC      3900
     GTGTCTCAGA AAAATCATGG CAAACAGGAA AAGCGTGAGC CCACAGGGCC ACAGGAACCC      3960
     AGTTACCTGC GTTTCATCCA GTCTCTTGCT GAGAACACAG GGTCTGTGAC TACGGATTCT      4020
     ACCGTGACTA CATCACCATA TGCTTTCACT CAGGTCACAG GGCCTTACAA CACATTTGTT      4080
     AATAACTGAT CATAATCAGC CATACCACAT TTGTAGAGGT TTTACTTGCT TTAAAAAACC      4140
     TCCCACACCT CCCCCTGAAC CTGAAACATA AAATGAATGC AATTGTTGTT GTTAACTTGT      4200
     TTATTGCAGC TTATAATGGT TACAAATAAA GCAATAGCAT CACAAATTTC ACAAATAAAG      4260
     CATTTTTTTC ACTGCATTCT AGTTGTGGTT TGTCCAAACT CATCAATGTA TCTTAAGGCG      4320
```

TAAATTGTAA GCGTTAATAT TTTGTTAAAA TTCGCGTTAA ATTTTTGTTA AATCAGCTCA 4380
TTTTTTAACC AATAGGCCGA AATCGGCAAA ATCCCTTATA AATCAAAAGA ATAGACCGAG 4440
ATAGGGTTGA GTGTTGTTCC AGTTTGGAAC AAGAGTCCAC TATTAAAGAA CGTGGACTCC 4500
AACGTCAAAG GGCGAAAAAC CGTCTATCAG GGCGATGGCC CACTACGTGA ACCATCACCC 4560
TAATCAAGTT TTTTGGGGTC GAGGTGCCGT AAAGCACTAA ATCGGAACCC TAAAGGGAGC 4620
CCCCGATTTA GAGCTTGACG GGGAAAGCCG GCGAACGTGG CGAGAAAGGA AGGGAAGAAA 4680
GCGAAAGGAG CGGGCGCTAG GGCGCTGGCA AGTGTAGCGG TCACGCTGCG CGTAACCACC 4740
ACACCCGCCG CGCTTAATGC GCCGCTACAG GGCGCGTCAG GTGGCACTTT TCGGGGAAAT 4800
GTGCGCGGAA CCCCTATTTG TTTATTTTTC TAAATACATT CAAATATGTA TCCGCTCATG 4860
AGACAATAAC CCTGATAAAT GCTTCAATAA TATTGAAAAA GGAAGAGTCC TGAGGCGGAA 4920
AGAACCAGCT GTGGAATGTG TGTCAGTTAG GGTGTGGAAA GTCCCCAGGC TCCCCAGCAG 4980
GCAGAAGTAT GCAAAGCATG CATCTCAATT AGTCAGCAAC CAGGTGTGGA AAGTCCCCAG 5040
GCTCCCCAGC AGGCAGAAGT ATGCAAAGCA TGCATCTCAA TTAGTCAGCA ACCATAGTCC 5100
CGCCCCTAAC TCCGCCCATC CCGCCCCTAA CTCCGCCCAG TTCCGCCCAT TCTCCGCCCC 5160
ATGGCTGACT AATTTTTTTT ATTTATGCAG AGGCCGAGGC CGCCTCGGCC TCTGAGCTAT 5220
TCCAGAAGTA GTGAGGAGGC TTTTTTGGAG GCCTAGGCTT TTGCAAAGAT CGATCAAGAG 5280
ACAGGATGAG GATCGTTTCG CATGATTGAA CAAGATGGAT TGCACGCAGG TTCTCCGGCC 5340
GCTTGGGTGG AGAGGCTATT CGGCTATGAC TGGGCACAAC AGACAATCGG CTGCTCTGAT 5400
GCCGCCGTGT TCCGGCTGTC AGCGCAGGGG CGCCCGGTTC TTTTTGTCAA GACCGACCTG 5460
TCCGGTGCCC TGAATGAACT GCAAGACGAG GCAGCGCGGC TATCGTGGCT GGCCACGACG 5520
GGCGTTCCTT GCGCAGCTGT GCTCGACGTT GTCACTGAAG CGGGAAGGGA CTGGCTGCTA 5580
TTGGGCGAAG TGCCGGGGCA GGATCTCCTG TCATCTCACC TTGCTCCTGC CGAGAAAGTA 5640
TCCATCATGG CTGATGCAAT GCGGCGGCTG CATACGCTTG ATCCGGCTAC CTGCCCATTC 5700
GACCACCAAG CGAAACATCG CATCGAGCGA GCACGTACTC GGATGGAAGC CGGTCTTGTC 5760
GATCAGGATG ATCTGGACGA AGAGCATCAG GGGCTCGCGC CAGCCGAACT GTTCGCCAGG 5820
CTCAAGGCGA GCATGCCCGA CGGCGAGGAT CTCGTCGTGA CCCATGGCGA TGCCTGCTTG 5880
CCGAATATCA TGGTGGAAAA TGGCCGCTTT TCTGGATTCA TCGACTGTGG CCGGCTGGGT 5940
GTGGCGGACC GCTATCAGGA CATAGCGTTG GCTACCCGTG ATATTGCTGA AGAGCTTGGC 6000
GGCGAATGGG CTGACCGCTT CCTCGTGCTT TACGGTATCG CCGCTCCCGA TTCGCAGCGC 6060
ATCGCCTTCT ATCGCCTTCT TGACGAGTTC TTCTGAGCGG GACTCTGGGG TTCGAAATGA 6120
CCGACCAAGC GACGCCCAAC CTGCCATCAC GAGATTTCGA TTCCACCGCC GCCTTCTATG 6180
AAAGGTTGGG CTTCGGAATC GTTTTCCGGG ACGCCGGCTG GATGATCCTC CAGCGCGGGG 6240
ATCTCATGCT GGAGTTCTTC GCCCACCCTA GGGGGAGGCT AACTGAAACA CGGAAGGAGA 6300
CAATACCGGA AGGAACCCGC GCTATGACGG CAATAAAAAG ACAGAATAAA ACGCACGGTG 6360
TTGGGTCGTT TGTTCATAAA CGCGGGGTTC GGTCCCAGGG CTGGCACTCT GTCGATACCC 6420
CACCGAGACC CCATTGGGGC CAATACGCCC GCGTTTCTTC CTTTTCCCCA CCCCACCCCC 6480
CAAGTTCGGG TGAAGGCCCA GGGCTCGCAG CCAACGTCGG GGCGGCAGGC CCTGCCATAG 6540
CCTCAGGTTA CTCATATATA CTTTAGATTG ATTTAAAACT TCATTTTTAA TTTAAAAGGA 6600
TCTAGGTGAA GATCCTTTTT GATAATCTCA TGACCAAAAT CCCTTAACGT GAGTTTTCGT 6660
TCCACTGAGC GTCAGACCCC GTAGAAAAGA TCAAAGGATC TTCTTGAGAT CCTTTTTTTC 6720
TGCGCGTAAT CTGCTGCTTG CAAACAAAAA AACCACCGCT ACCAGCGGTG GTTTGTTTGC 6780
CGGATCAAGA GCTACCAACT CTTTTTCCGA AGGTAACTGG CTTCAGCAGA GCGCAGATAC 6840
CAAATACTGT CCTTCTAGTG TAGCCGTAGT TAGGCCACCA CTTCAAGAAC TCTGTAGCAC 6900
CGCCTACATA CCTCGCTCTG CTAATCCTGT TACCAGTGGC TGCTGCCAGT GGCGATAAGT 6960
CGTGTCTTAC CGGGTTGGAC TCAAGACGAT AGTTACCGGA TAAGGCGCAG CGGTCGGGCT 7020
GAACGGGGGG TTCGTGCACA CAGCCCAGCT TGGAGCGAAC GACCTACACC GAACTGAGAT 7080
ACCTACAGCG TGAGCTATGA GAAAGCGCCA CGCTTCCCGA AGGGAGAAAG GCGGACAGGT 7140
ATCCGGTAAG CGGCAGGGTC GGAACAGGAG AGCGCACGAG GGAGCTTCCA GGGGGAAACG 7200
CCTGGTATCT TTATAGTCCT GTCGGGTTTC GCCACCTCTG ACTTGAGCGT CGATTTTTGT 7260
GATGCTCGTC AGGGGGGCGG AGCCTATGGA AAAACGCCAG CAACGCGGCC TTTTTACGGT 7320
TCCTGGCCTT TTGCTGGCCT TTTGCTCACA TGTTCTTTCC TGCGTTATCC CCTGATTCTG 7380
TGGATAACCG TATTACCGCC ATGCAT 7406

>pEYFP-Tet2 CD/CI (H1295Y/D1297A)

```
TAGTTATTAA TAGTAATCAA TTACGGGGTC ATTAGTTCAT AGCCCATATA TGGAGTTCCG     60
CGTTACATAA CTTACGGTAA ATGGCCCGCC TGGCTGACCG CCCAACGACC CCCGCCCATT    120
GACGTCAATA ATGACGTATG TTCCCATAGT AACGCCAATA GGGACTTTCC ATTGACGTCA    180
ATGGGTGGAG TATTTACGGT AAACTGCCCA CTTGGCAGTA CATCAAGTGT ATCATATGCC    240
AAGTACGCCC CCTATTGACG TCAATGACGG TAAATGGCCC GCCTGGCATT ATGCCCAGTA    300
CATGACCTTA TGGGACTTTC CTACTTGGCA GTACATCTAC GTATTAGTCA TCGCTATTAC    360
CATGGTGATG CGGTTTTGGC AGTACATCAA TGGGCGTGGA TAGCGGTTTG ACTCACGGGG    420
ATTTCCAAGT CTCCACCCCA TTGACGTCAA TGGGAGTTTG TTTTGGCACC AAAATCAACG    480
GGACTTTCCA AAATGTCGTA ACAACTCCGC CCCATTGACG CAAATGGGCG GTAGGCGTGT    540
ACGGTGGGAG GTCTATATAA GCAGAGCTGG TTTAGTGAAC CGTCAGATCC GCTAGCGCTA    600
CCGGTCGCCA CCATGGTGAG CAAGGGCGAG GAGCTGTTCA CCGGGGTGGT GCCCATCCTG    660
GTCGAGCTGG ACGGCGACGT AAACGGCCAC AAGTTCAGCG TGTCCGGCGA GGGCGAGGGC    720
GATGCCACCT ACGGCAAGCT GACCCTGAAG TTCATCTGCA CCACCGGCAA GCTGCCCGTG    780
CCCTGGCCCA CCCTCGTGAC CACCTTCGGC TACGGCCTGC AGTGCTTCGC CCGCTACCCC    840
GACCACATGA AGCAGCACGA CTTCTTCAAG TCCGCCATGC CCGAAGGCTA CGTCCAGGAG    900
CGCACCATCT TCTTCAAGGA CGACGGCAAC TACAAGACCC GCGCCGAGGT GAAGTTCGAG    960
GGCGACACCC TGGTGAACCG CATCGAGCTG AAGGGCATCG ACTTCAAGGA GGACGGCAAC   1020
ATCCTGGGGC ACAAGCTGGA GTACAACTAC AACAGCCACA ACGTCTATAT CATGGCCGAC   1080
AAGCAGAAGA ACGGCATCAA GGTGAACTTC AAGATCCGCC ACAACATCGA GGACGGCAGC   1140
GTGCAGCTCG CCGACCACTA CCAGCAGAAC ACCCCCATCG GCGACGGCCC CGTGCTGCTG   1200
CCCGACAACC ACTACCTGAG CTACCAGTCC GCCCTGAGCA AAGACCCCAA CGAGAAGCGC   1260
GATCACATGG TCCTGCTGGA GTTCGTGACC GCCGCCGGGA TCACTCTCGG CATGGACGAG   1320
CTGTACAAGT CCGGACTCAG ATCTCGAGCT CAAGCTTCGA ATTCTGCAGT CGACGGTACC   1380
GCGGGCCCGG CGCTTATGGA CTACAAAGAC GATGACGACA AGGGATCCAC TAGTAACGGC   1440
CGCCAGTGTG CTGGAATTCG CCCTTTACAA AGTCAGAATG GCAAATGTGA AGGATGCAAT   1500
CCAGACAAAG ATGAAGCTCC TTATTATACC CATCTGGGAG CTGGTCCTGA TGTGGCAGCT   1560
ATTAGAACAC TCATGGAAGA AAGGTATGGA GAGAAGGGTA AAGCTATTAG GATTGAAAAA   1620
GTCATATATA CTGGTAAAGA AGGCAAGAGC TCTCAGGGAT GTCCTATTGC TAAATGGGTA   1680
TATCGGAGAT CGAGTGAGGA GGAGAAACTA CTGTGTTTGG TACGAGTGCG ACCTAATCAC   1740
ACATGTGAGA CGGCGGTGAT GGTAATTGCC ATCATGTTGT GGGACGGAAT CCCAAAGCTA   1800
CTCGCATCAG AACTCTACTC AGAACTTACA GATATCTTGG GCAAGTGTGG CATATGCACC   1860
AACCGTCGCT GTTCTCAGAA TGAAACTAGA AACTGTTGTT GTCAGGGTGA GAATCCAGAG   1920
ACCTGTGGTG CCTCCTTTTC TTTTGGTTGT TCTTGGAGCA TGTACTATAA TGGATGTAAG   1980
TTTGCCAGAA GCAAGAAACC AAGGAAATTT AGGCTACATG GAGCTGAGCC AAAAGAGGAA   2040
GAGAGACTAG GTTCTCATTT GCAAAACCTG GCTACTGTCA TTGCTCCAAT ATACAAGAAG   2100
CTTGCACCCG ATGCATACAA TAATCAGGTT GAATTTGAAC ACCAAGCCCC AGACTGCTGT   2160
TTGGGTCTGA AGGAAGGCCG GCCATTCTCA GGAGTCACTG CATGTTTGGA CTTCTCTGCT   2220
CATTCCtACA GAGcCCAGCA GAACATGCCA AATGGCAGTA CAGTGGTGGT CACCCTCAAT   2280
AGAGAAGACA ATCGAGAAGT CGGAGCTAAG CCTGAGGATG AGCAGTTCCA CGTGCTGCCT   2340
ATGTACATCA TCGCCCCTGA GGATGAGTTT GGGAGTACGG AAGGCCAGGA GAAGAAGATA   2400
CGGATGGGGT CCATTGAGGT TCTGCAGTCA TTTCGGAGGA GAAGGGTCAT AAGGATAGGA   2460
GAGCTGCCCA AGAGTTGCAA GAAGAAAGCG GAGCCCAAGA AAGCCAAGAC CAAGAAAGCA   2520
GCTCGAAAGC GTTCCTCTCT GGAGAACTGC TCCAGTAGGA CTGAGAAGGG AAAGTCTTCC   2580
TCACATACAA AGCTGATGGA AAATGCAAGC CATATGAAAC AAATGACAGC ACAACCGCAG   2640
CTTTCGGGCC CGGTCATCCG GCAGCCACCA ACACTCCAGA GGCACCTTCA GCAAGGGCAG   2700
AGGCCACAGC AGCCGCAGCC ACCTCAGCCG CAGCCGCAGA CGACACCTCA GCCACAGCCA   2760
CAGCCACAGC ATATCATGCC CGGTAACTCT CAGTCTGTTG GTTCTCATTG TTCTGGATCC   2820
ACCAGTGTCT ACACGAGACA GCCTACTCCT CACAGTCCTT ATCCCAGCTC AGCACACACC   2880
TCAGATATTT ATGGAGATAC CAACCATGTG AACTTTTACC CCACTTCATC TCATGCCTCG   2940
GGTTCATATT TGAATCCTTC TAATTACATG AACCCCTACC TTGGGCTTTT GAATCAGAAT   3000
AACCAATATG CACCTTTTCC ATACAATGGG AGTGTGCCAG TGGACAATGG TTCCCCTTTC   3060
TTAGGTTCTT ATTCCCCCCA GGCTCAGTCC AGGGATCTAC ATAGATATCC AAACCAGGAC   3120
CATCTCACCA ATCAGAACTT ACCACCCATC CACACCCTTC ACCAACAGAC GTTTGGGGAC   3180
AGTCCCTCTA AGTACTTAAG TTATGGAAAC CAAAATATGC AGAGAGATGC CTTCACTACT   3240
AACTCCACCC TAAAACCAAA TGTACACCAC CTAGCAACGT TTTCTCCTTA CCCCACCCCC   3300
AAGATGGATA GTCATTTCAT GGGAGCTGCC TACAGATCAC CATACAGCCA CCCACACACT   3360
GACTACAAAA CCAGTGAGCA TCATCTACCC TCTCACACGG TCTACAGCTA CACGGCAGCA   3420
GCTTCGGGGA GCAGTTCCAG CCACGCCTTC CACAACAAGG AGAATGACAA CATAGCCAAT   3480
GGGCTCTCAA GAGTGCTTCC AGGGTTTAAT CATGATAGAA CTGCTTCTGC CCAAGAACTA   3540
TTATACAGTC TGACTGGCAG CAGTCAGGAG AAGCAGCCTG AGGTGTCAGG CCAGGATGCA   3600
GCTGCTGTGC AGGAAATTGA GTATTGGTCA GATAGTGAGC ACAACTTTCA GGATCCTTGC   3660
ATTGGAGGGG TGGCTATAGC CCCAACTCAT GGGTCAATTC TTATTGAGTG TGCAAAGTGT   3720
GAGGTTCATG CCACAACCAA AGTAAACGAT CCCGACCGGA ATCACCCCAC CAGGATCTCA   3780
CTTGTACTGT ATAGGCATAA GAATTTGTTT CTACCAAAAC ATTGTTTGGC TCTCTGGGAA   3840
GCCAAAATGG CTGAAAAGGC CCGGAAAGAG GAAGAGTGCG GAAAGAATGG ATCAGACCAC   3900
GTGTCTCAGA AAAATCATGG CAAACAGGAA AAGCGTGAGC CCACAGGGCC ACAGGAACCC   3960
AGTTACCTGC GTTTCATCCA GTCTCTTGCT GAGAACACAG GGTCTGTGAC TACGGATTCT   4020
ACCGTGACTA CATCACCATA TGCTTTCACT CAGGTCACAG GGCCTTACAA CACATTTGTT   4080
AATAACTGAT CATAATCAGC CATACCACAT TTGTAGAGGT TTTACTTGCT TTAAAAAACC   4140
TCCCACACCT CCCCCTGAAC CTGAAACATA AAATGAATGC AATTGTTGTT GTTAACTTGT   4200
TTATTGCAGC TTATAATGGT TACAAATAAA GCAATAGCAT CACAAATTTC ACAAATAAAG   4260
CATTTTTTTC ACTGCATTCT AGTTGTGGTT TGTCCAAACT CATCAATGTA TCTTAAGGCG   4320
TAAATTGTAA GCGTTAATAT TTTGTTAAAA TTCGCGTTAA ATTTTTGTTA AATCAGCTCA   4380
TTTTTTAACC AATAGGCCGA AATCGGCAAA ATCCCTTATA AATCAAAAGA ATAGACCGAG   4440
ATAGGGTTGA GTGTTGTTCC AGTTTGGAAC AAGAGTCCAC TATTAAAGAA CGTGGACTCC   4500
AACGTCAAAG GGCGAAAAAC CGTCTATCAG GGCGATGGCC CACTACGTGA ACCATCACCC   4560
TAATCAAGTT TTTTGGGGTC GAGGTGCCGT AAAGCACTAA ATCGGAACCC TAAAGGGAGC   4620
```

CCCCGATTTA GAGCTTGACG GGGAAAGCCG GCGAACGTGG CGAGAAAGGA AGGGAAGAAA 4680
GCGAAAGGAG CGGGCGCTAG GGCGCTGGCA AGTGTAGCGG TCACGCTGCG CGTAACCACC 4740
ACACCCGCCG CGCTTAATGC GCCGCTACAG GGCGCGTCAG GTGGCACTTT TCGGGGAAAT 4800
GTGCGCGGAA CCCCTATTTG TTTATTTTTC TAAATACATT CAAATATGTA TCCGCTCATG 4860
AGACAATAAC CCTGATAAAT GCTTCAATAA TATTGAAAAA GGAAGAGTCC TGAGGCGGAA 4920
AGAACCAGCT GTGGAATGTG TGTCAGTTAG GGTGTGGAAA GTCCCCAGGC TCCCCAGCAG 4980
GCAGAAGTAT GCAAAGCATG CATCTCAATT AGTCAGCAAC CAGGTGTGGA AAGTCCCCAG 5040
GCTCCCCAGC AGGCAGAAGT ATGCAAAGCA TGCATCTCAA TTAGTCAGCA ACCATAGTCC 5100
CGCCCCTAAC TCCGCCCATC CCGCCCCTAA CTCCGCCCAG TTCCGCCCAT TCTCCGCCCC 5160
ATGGCTGACT AATTTTTTTT ATTTATGCAG AGGCCGAGGC CGCCTCGGCC TCTGAGCTAT 5220
TCCAGAAGTA GTGAGGAGGC TTTTTTGGAG GCCTAGGCTT TTGCAAAGAT CGATCAAGAG 5280
ACAGGATGAG GATCGTTTCG CATGATTGAA CAAGATGGAT TGCACGCAGG TTCTCCGGCC 5340
GCTTGGGTGG AGAGGCTATT CGGCTATGAC TGGGCACAAC AGACAATCGG CTGCTCTGAT 5400
GCCGCCGTGT TCCGGCTGTC AGCGCAGGGG CGCCCGGTTC TTTTTGTCAA GACCGACCTG 5460
TCCGGTGCCC TGAATGAACT GCAAGACGAG GCAGCGCGGC TATCGTGGCT GGCCACGACG 5520
GGCGTTCCTT GCGCAGCTGT GCTCGACGTT GTCACTGAAG CGGGAAGGGA CTGGCTGCTA 5580
TTGGGCGAAG TGCCGGGGCA GGATCTCCTG TCATCTCACC TTGCTCCTGC CGAGAAAGTA 5640
TCCATCATGG CTGATGCAAT GCGGCGGCTG CATACGCTTG ATCCGGCTAC CTGCCCATTC 5700
GACCACCAAG CGAAACATCG CATCGAGCGA GCACGTACTC GGATGGAAGC CGGTCTTGTC 5760
GATCAGGATG ATCTGGACGA AGAGCATCAG GGGCTCGCGC CAGCCGAACT GTTCGCCAGG 5820
CTCAAGGCGA GCATGCCCGA CGGCGAGGAT CTCGTCGTGA CCCATGGCGA TGCCTGCTTG 5880
CCGAATATCA TGGTGGAAAA TGGCCGCTTT TCTGGATTCA TCGACTGTGG CCGGCTGGGT 5940
GTGGCGGACC GCTATCAGGA CATAGCGTTG GCTACCCGTG ATATTGCTGA AGAGCTTGGC 6000
GGCGAATGGG CTGACCGCTT CCTCGTGCTT TACGGTATCG CCGCTCCCGA TTCGCAGCGC 6060
ATCGCCTTCT ATCGCCTTCT TGACGAGTTC TTCTGAGCGG GACTCTGGGG TTCGAAATGA 6120
CCGACCAAGC GACGCCCAAC CTGCCATCAC GAGATTTCGA TTCCACCGCC GCCTTCTATG 6180
AAAGGTTGGG CTTCGGAATC GTTTTCCGGG ACGCCGGCTG GATGATCCTC CAGCGCGGGG 6240
ATCTCATGCT GGAGTTCTTC GCCCACCCTA GGGGGAGGCT AACTGAAACA CGGAAGGAGA 6300
CAATACCGGA AGGAACCCGC GCTATGACGG CAATAAAAAG ACAGAATAAA ACGCACGGTG 6360
TTGGGTCGTT TGTTCATAAA CGCGGGGTTC GGTCCCAGGG CTGGCACTCT GTCGATACCC 6420
CACCGAGACC CCATTGGGGC CAATACGCCC GCGTTTCTTC CTTTTCCCCA CCCCACCCCC 6480
CAAGTTCGGG TGAAGGCCCA GGGCTCGCAG CCAACGTCGG GGCGGCAGGC CCTGCCATAG 6540
CCTCAGGTTA CTCATATATA CTTTAGATTG ATTTAAAACT TCATTTTTAA TTTAAAAGGA 6600
TCTAGGTGAA GATCCTTTTT GATAATCTCA TGACCAAAAT CCCTTAACGT GAGTTTTCGT 6660
TCCACTGAGC GTCAGACCCC GTAGAAAAGA TCAAAGGATC TTCTTGAGAT CCTTTTTTTC 6720
TGCGCGTAAT CTGCTGCTTG CAAACAAAAA AACCACCGCT ACCAGCGGTG GTTTGTTTGC 6780
CGGATCAAGA GCTACCAACT CTTTTTCCGA AGGTAACTGG CTTCAGCAGA GCGCAGATAC 6840
CAAATACTGT CCTTCTAGTG TAGCCGTAGT TAGGCCACCA CTTCAAGAAC TCTGTAGCAC 6900
CGCCTACATA CCTCGCTCTG CTAATCCTGT TACCAGTGGC TGCTGCCAGT GGCGATAAGT 6960
CGTGTCTTAC CGGGTTGGAC TCAAGACGAT AGTTACCGGA TAAGGCGCAG CGGTCGGGCT 7020
GAACGGGGGG TTCGTGCACA CAGCCCAGCT TGGAGCGAAC GACCTACACC GAACTGAGAT 7080
ACCTACAGCG TGAGCTATGA GAAAGCGCCA CGCTTCCCGA AGGGAGAAAG GCGGACAGGT 7140
ATCCGGTAAG CGGCAGGGTC GGAACAGGAG AGCGCACGAG GGAGCTTCCA GGGGGAAACG 7200
CCTGGTATCT TTATAGTCCT GTCGGGTTTC GCCACCTCTG ACTTGAGCGT CGATTTTTGT 7260
GATGCTCGTC AGGGGGGCGG AGCCTATGGA AAAACGCCAG CAACGCGGCC TTTTTACGGT 7320
TCCTGGCCTT TTGCTGGCCT TTTGCTCACA TGTTCTTTCC TGCGTTATCC CCTGATTCTG 7380
TGGATAACCG TATTACCGCC ATGCAT 7406

Supplemental Table S7

Substrate used in Figures 1A and 2A

```
GGTACCCGGG GATCCTCTAG AGTCGACCTG CAGGCATGCA AGCTTGGCGT AATCATGGTC      60
ATAGCTGTTT CCTGTGTGAA ATTGTTATCC GCTCACAATT CCACACAACA TACGAGCCGG     120
AAGCATAAAG TGTAAAGCCT GGGGTGCCTA ATGAGTGAGC TAACTCACAT TAATTGCGTT     180
GCGCTCACTG CCCGCTTTCC AGTCGGGAAA CCTGTCGTGC CAGCTGCATT AATGAATCGG     240
CCAACGCGCG GGGAGAGGCG GTTTGCGTAT TGGGCGCTCT TCCGCTTCCT CGCTCACTGA     300
CTCGCTGCGC TCGGTCGTTC GGCTGCGGCG AGCGGTATCA GCTCACTCAA AGGCGGTAAT     360
ACGGTTATCC ACAGAATCAG GGGATAACGC AGGAAAGAAC ATGTGAGCAA AAGGCCAGCA     420
AAAGGCCAGG AACCGTAAAA AGGCCGCGTT GCTGGCGTTT TTCCATAGGC TCCGCCCCCC     480
TGACGAGCAT CACAAAAATC GACGCTCAAG TCAGAGGTGG CGAAACCCGA CAGGACTATA     540
AAGATACCAG GGTTTCCCCC TGGAAGCTCC CTCGTGCGCT CTCCTGTTCC GACCCTGCCG     600
CTTACCGGAT ACCTGTCCGC CTTTCTCCCT TCGGGAAGCG TGGCGCTTTC TCATAGCTCA     660
CGCTGTAGGT ATCTCAGTTC GGTGTAGGTC GTTCGCTCCA AGCTGGGCTG TGTGCACGAA     720
CCCCCCGTTC AGCCCGACCG CTGCGCCTTA TCCGGTAACT ATCGTCTTGA GTCCAACCCG     780
GTAAGACACG ACTTATCGCC ACTGGCAGCA GCCACTGGTA ACAGGATTAG CAGAGCGAGG     840
TATGTAGGCG GTGCTACAGA GTTCTTGAAG TGGTGGCCTA ACTACGGCTA CACTAGAAGA     900
ACAGTATTTG GTATCTGCGC TCTGCTGAAG CCAGTTACCT TCGGAAAAAG AGTTGGTAGC     960
TCTTGATCCG GCAAACAAAC CACCGCTGGT AGCGGTGGTT TTTTTGTTTG CAAGCAGCAG    1020
ATTACGCGCA GAAAAAAAGG ATCTCAAGAA GATCCTTTGA TCTTTTCTAC GGGGTCTGAC    1080
GCTCAGTGGA ACGAAAACTC ACGTTAAGGG ATTTTGGTCA TGAGATTATC AAAAAGGATC    1140
TTCACCTAGA TCCTTTTAAA TTAAAAATGA AGTTTTAAAT CAATCTAAAG TATATATGAG    1200
TAAACTTGGT CTGACAGTTA CCAATGCTTA ATCAGTGAGG CACCTATCTC AGCGATCTGT    1260
CTATTTCGTT CATCCATAGT TGCCTGACTC CCCGTCGTGT AGATAACTAC GATACGGGAG    1320
GGCTTACCAT CTGGCCCCAG TGCTGCAATG ATACCGCGAG ACCCACGCTC ACCGGCTCCA    1380
GATTTATCAG CAATAAACCA GCCAGCCGGA AGGGCCGAGC GCAGAAGTGG TCCTGCAACT    1440
TTATCCGCCT CCATCCAGTC TATTAATTGT TGCCGGGAAG CTAGAGTAAG TAGTTCGCCA    1500
GTTAATAGTT TGCGCAACGT TGTTGCCATT GCTACAGGCA TCGTGGTGTC ACGCTCGTCG    1560
TTTGGTATGG CTTCATTCAG CTCCGGTTCC CAACGATCAA GGCGAGTTAC ATGATCCCCC    1620
ATGTTGTGCA AAAAAGCGGT TAGCTCCTTC GGTCCTCCGA TCGTTGTCAG AAGTAAGTTG    1680
GCCGCAGTGT TATCACTCAT GGTTATGGCA GCACTGCATA ATTCTCTTAC TGTCATGCCA    1740
TCCGTAAGAT GCTTTTCTGT GACTGGTGAG TACTCAACCA AGTCATTCTG AGAATAGTGT    1800
ATGCGGCGAC CGAGTTGCTC TTGCCCGGCG TCAATACGGG ATAATACCGC GCCACATAGC    1860
AGAACTTTAA AAGTGCTCAT CATTGGAAAA CGTTCTTCGG GGCGAAAACT CTCAAGGATC    1920
TTACCGCTGT TGAGATCCAG TTCGATGTAA CCCACTCGTG CACCCAACTG ATCTTCAGCA    1980
TCTTTTACTT TCACCAGCGT TTCTGGGTGA GCAAAAACAG GAAGGCAAAA TGCCGCAAAA    2040
AAGGGAATAA GGGCGACACG GAAATGTTGA ATACTCATAC TCTTCCTTTT TCAATATTAT    2100
TGAAGCATTT ATCAGGGTTA TTGTCTCATG AGCGGATACA TATTTGAATG TATTTAGAAA    2160
AATAAACAAA TAGGGGTTCC GCGCACATTT CCCCGAAAAG TGCCACCTGA CGTCTAAGAA    2220
ACCATTATTA TCATGACATT AACCTATAAA AATAGGCGTA TCACGAGGCC CTTTCGTCTC    2280
GCGCGTTTCG GTGATGACGG TGAAAACCTC TGACACATGC AGCTCCCGGA GACGGTCACA    2340
GCTTGTCTGT AAGCGGATGC CGGGAGCAGA CAAGCCCGTC AGGGCGCGTC AGCGGGTGTT    2400
GGCGGGTGTC GGGGCTGGCT TAACTATGCG GCATCAGAGC AGATTGTACT GAGAGTGCAC    2460
CATATGCGGT GTGAAATACC GCACAGATGC GTAAGGAGAA AATACCGCAT CAGGCGCCAT    2520
TCGCCATTCA GGCTGCGCAA CTGTTGGGAA GGGCGATCGG TGCGGGCCTC TTCGCTATTA    2580
CGCCAGCTGG CGAAAGGGGG ATGTGCTGCA AGGCGATTAA GTTGGGTAAC GCCAGGGTTT    2640
TCCCAGTCAC GACGTTGTAA AACGACGGCC AGTGAATTCG AGCTCGGTAC CCGGGGATCC    2700
TCTAGAGT -- Biotin                                                  2708
```
